# Self-assembly of iron porphyrin complexes bearing carboxyl and carboxylate groups for a highly active CO_2_ reduction framework catalyst

**DOI:** 10.1039/d5sc10220k

**Published:** 2026-07-16

**Authors:** Xianjun Li, Kento Kosugi, Maho Imai, Ritsu Nagai, Zi Lang Goo, Chihoko Fukakusa, Teppei Yamada, Masaki Uchida, Hidehiro Uekusa, Shinpei Kusaka, Ryotaro Matsuda, Yutaka Saga, Tetsuya Kambe, Shigeyuki Masaoka, Mio Kondo

**Affiliations:** a Department of Chemistry, School of Science, Institute of Science Tokyo 2-12-1 Ookayama, Meguro Tokyo 152-8550 Japan mio@chem.sci.isct.ac.jp; b Department of Chemistry, Graduate School of Science, The University of Osaka 1-1 Machikaneyama, Toyonaka Osaka 560-0043 Japan; c Department of Chemistry, Graduate School of Science, The University of Tokyo 7-3-1 Hongo, Bunkyo Tokyo 113-0033 Japan; d Department of Chemistry and Biotechnology, School of Engineering, and Department of Material Chemistry, Graduate School of Engineering, Nagoya University Furo-cho, Chikusa-ku Nagoya 464-8603 Aichi Japan; e Division of Applied Chemistry, Graduate School of Engineering, The University of Osaka 2-1 Yamadaoka, Suita Osaka 565-0871 Japan masaoka@chem.eng.osaka-u.ac.jp; f Innovate Catalysis Science Division, Institute for Open and Transdisciplinary Research Initiative (ICS-OTRI), The University of Osaka 2-1 Yamadaoka, Suita Osaka 565-0871 Japan

## Abstract

Developing efficient catalysts for CO_2_ reduction is a promising approach to address energy and environmental challenges. Molecule-based heterogeneous catalysts are attractive because they combine the advantages of homogeneous and heterogeneous catalysts. Achieving an efficient CO_2_ reduction catalyst requires the integration of the functions that facilitate the following three key processes; (i) chemical conversion, (ii) substrate accumulation, and (iii) proton transport. However, such function-integrated systems for CO_2_ reduction have not been reported thus far. In this study, we integrated a porous structure and a hydrogen-bonding network *via* the self-assembly of a molecular catalyst containing an iron porphyrin complex, which is a well-known catalytic center for CO_2_ reduction. The resulting material exhibited the highest CO_2_ reduction activity under photochemical conditions (1.8 × 10^6^ µmol g^−1^ h^−1^ for CO production, selectivity > 99%) among the related systems owing to its function-integrated crystalline structure. This study provides a versatile strategy for designing highly active molecule-based heterogeneous catalysts for efficient CO_2_ reduction.

## Abstract 2

This study provides a new design principle for molecule-based heterogeneous catalysts, in which proton transport, CO_2_ accumulation, and molecular catalysis are integrated within a single crystalline framework.

## Graphical abstract

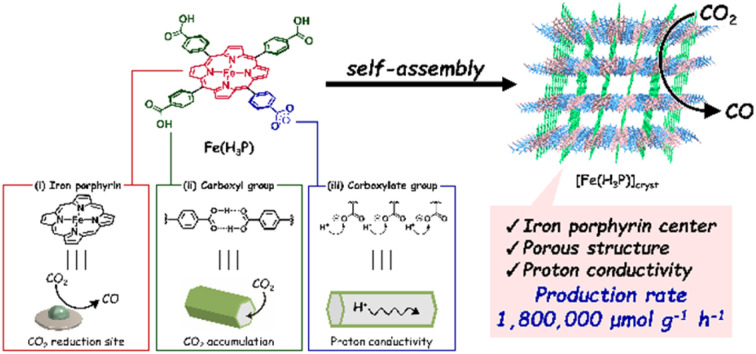

## Introduction

The catalytic reduction of CO_2_ (*e.g.*, CO_2_ + 2H^+^ + 2e^−^ → CO + H_2_O) offers a promising approach that addresses energy and environmental problems because this technology can potentially reduce atmospheric CO_2_ concentration and produce renewable fuels.^1^ Extensive research has focused on developing efficient catalysts for CO_2_ reduction,^2–55^ broadly classified as homogeneous and heterogeneous catalysts. Homogeneous catalysts, particularly metal-complex-based catalysts, offer design flexibility owing to their molecular nature, enabling highly active and selective CO_2_ conversion.^2–5^ In contrast, heterogeneous catalysts exhibit superior durability and recyclability owing to their solid nature.^6–16^ Therefore, developing molecule-based heterogeneous catalysts that integrate the advantages of both systems is a compelling approach.

**Scheme 1:**
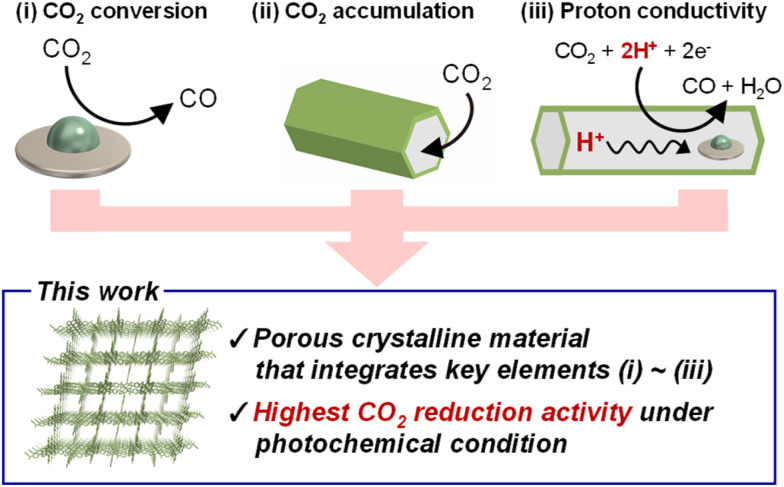
Key elements to fabricate efficient molecule-based heterogeneous catalysts for CO_2_ reduction (top) and a summary of this study (bottom).

However, the direct aggregation of homogeneous materials often results in densely packed crystalline structures, which prevents the mass transport of substrates required for the catalysis, CO_2_ and protons, to the catalytic centers. To overcome this limitation, molecule-based porous crystalline materials, such as metal–organic frameworks (MOFs),^19–44,56–59^ covalent-organic frameworks (COFs)^45–54^ and hydrogen-bonding frameworks (HOFs),^55^ are fascinating candidates. Actually, these materials, including MOF systems incorporating iron porphyrin as a catalytic site,^60,61^ have been widely studied as CO_2_ reduction catalysts.^17–54^ The first important feature of this class of materials is the metal-complex-based catalytic center for CO_2_ reduction ((i) in Scheme 1).^19–44^ Second, their porous structure promotes the accumulation of CO_2_ ((ii) in Scheme 1).^62–69^ Furthermore, it is also independently known that appropriate design of a porous structure such as hydrogen bonding networks enables high proton conductivity ((iii) in Scheme 1).^70–79^ Therefore, the construction of porous crystalline solids with catalytically active and proton conductive sites is expected to yield highly active CO_2_ reduction catalysts that incorporate the advantages of homogeneous and heterogeneous systems. However, materials with all the abovementioned essential elements for CO_2_ reduction, including (i) metal-complex-based catalytic sites for CO_2_ conversion, (ii) porous structures for CO_2_ accumulation, and (iii) hydrogen-bonding networks for proton conduction, have not been reported thus far because integrating catalytic sites and proton-conductive pathways within a rigid crystalline lattice remains fundamentally challenging.

In this study, we report the first example of a molecule-based porous crystalline material that meets all essential requirements for efficient CO_2_ reduction. Through the self-assembly of iron-complex-based catalyst modules, we constructed a porous framework structure with catalytically active and proton-conductive sites. This material demonstrated the highest catalytic activity for CO_2_ to CO conversion among comparable systems, along with high stability during catalysis. We also performed a series of experiments to investigate the material's physical properties and validate our design strategy.

## Results & discussion

**Scheme 2:**
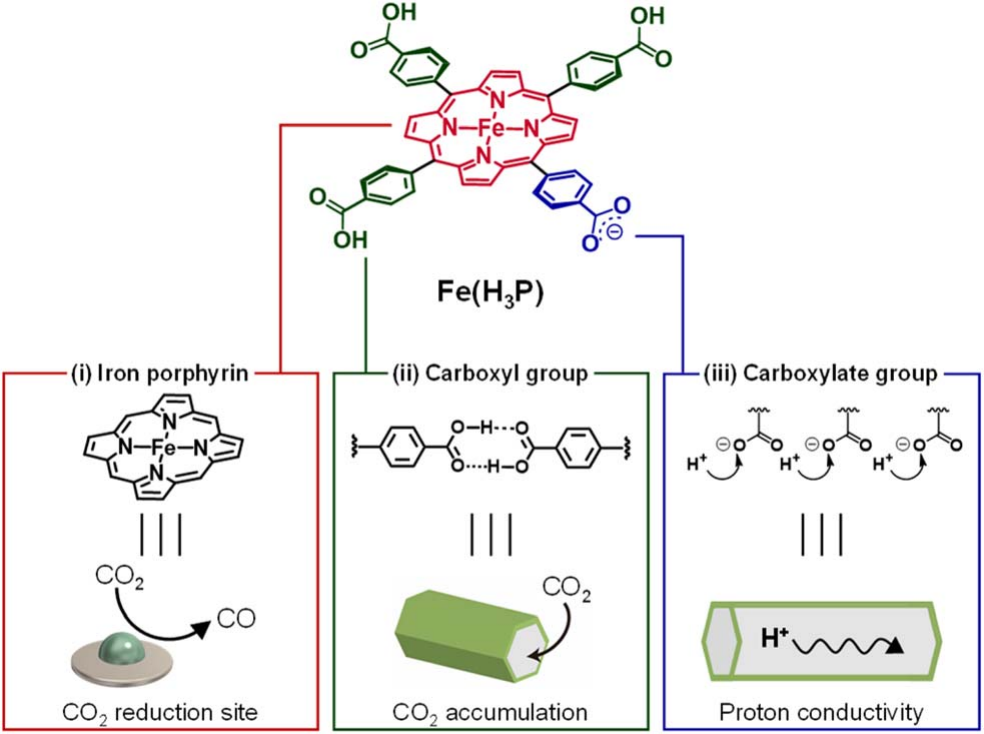
Structure and features of the metal-complex-based catalyst module Fe(H_3_P) used in this study.

The key to our success arises from the self-assembly of metal-complex-based catalyst modules bearing carboxyl and carboxylate groups that enable three critical functions: metal-complex-based catalytic centers exhibiting high catalytic activity for CO_2_ reduction,^2–5^ carboxyl groups forming a porous structure suitable for CO_2_ accumulation *via* hydrogen bonding,^79^ and monodentate or dangling carboxylate groups serving as a proton reservoir to enhance proton conductivity.^72,75,79^ As the catalytic centre, we employed an iron porphyrin complex, which is widely used in both homogeneous systems^80–87,89^ and molecule-based heterogeneous systems,^88,91^ including MOFs.^60,61^ Based on this design, we used an iron porphyrin complex bearing three carboxyl groups and one carboxylate group on phenyl rings at the *meso* positions, 5,10,15-tris(4-carboxyphenyl)-20-(4-carboxylatophenyl)porphyrinato iron(iii), Fe(H_3_P), as the catalyst module (Scheme 2).

**Fig. 1:**
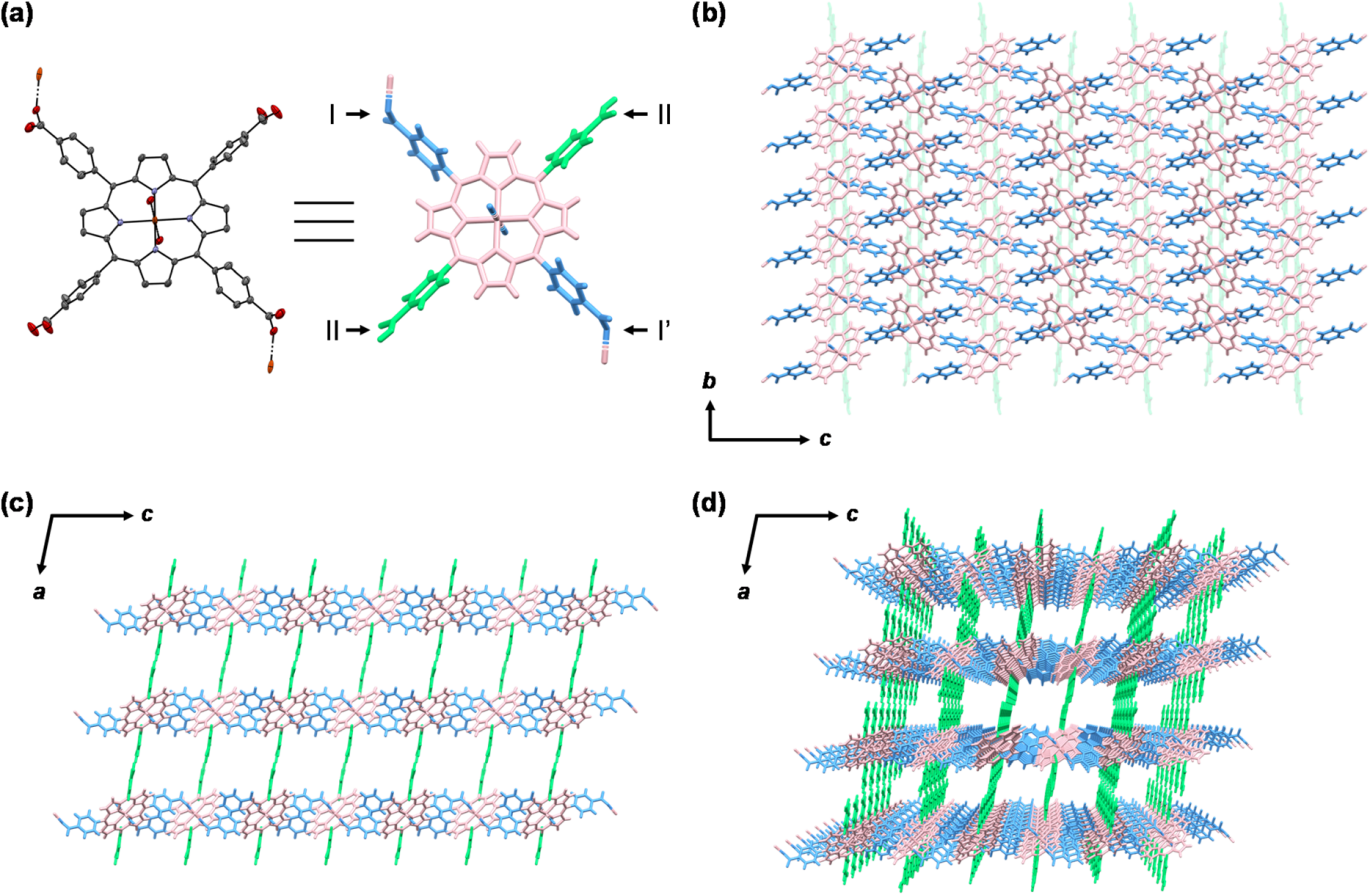
(a) ORTEP drawing (left, 50% probability ellipsoids) and capped stick representation (right) of Fe(H_3_P). In ORTEP drawing, C = gray, N = purple, Fe = orange and H atoms are omitted for clarity. In the capped stick representation, pink = porphyrin scaffold, blue = carboxylate group and green = carboxyl group. (b) 2D layer structure observed in [Fe(H_3_P)]_cryst_. (c) Interlayer interactions observed in [Fe(H_3_P)]_cryst_. (d) Packing structure of [Fe(H_3_P)]_cryst_ along the *b* axis.

The crystal of Fe(H_3_P) was obtained as follows: 5,10,15,20-tetrakis(4-carboxyphenyl)porphyrinato iron(iii) chloride, Fe(H_4_P), was synthesized according to a modification of the literature^90^ (see p. S5 in the SI). Subsequently, Fe(H_4_P) was deprotonated *in situ* in the reaction mixture under basic condition (KOH in methanol, acidified to pH 3 to 5) and heated at 50 °C overnight, affording the self-assembled crystal structure of Fe(H_3_P), [Fe(H_3_P)]_cryst_. The molecular structure of Fe(H_3_P) is shown in Fig. 1a, containing one carboxylate group disordered over two sites (I and I′ in Fig. 1a) (Fig. S1 and Table S1). The remaining substituents (II in Fig. 1a) are assigned to the carboxyl groups based on the charge neutrality of the molecule, confirming that Fe(H_3_P) contains one carboxylate and three carboxyl groups. The packing structure of Fe(H_3_P) is shown in Fig. 1b–d. One oxygen atom of each carboxylate group in Fe(H_3_P) is coordinated with the iron centers of an adjacent iron porphyrin module in a monodentate manner, forming a 2D layer (Fig. 1b and S2a), which is further assembled *via* hydrogen-bonding interactions (Fig. 1c and S2b), resulting in the formation of a porous framework structure, [Fe(H_3_P)]_cryst_ (Fig. 1d, for details of the deprotonation state, see the SI (pp. S12–S13, Fig. S3 and Table S2)). The pore entrance size was 7.93 Å × 6.95 Å, based on the van der Waals radii of the constituent atoms. Gas adsorption analysis reveals that this porous material can accumulate CO_2_ within its pores (Fig. S4). Although the amount of CO_2_ adsorbed (0.65 mmol g^−1^) is moderate compared with that of relevant systems (Table S3), the non-porous iron porphyrin complex does not exhibit CO_2_ adsorption at all^91^ (Fig. S4), indicating the importance of the porous structure for CO_2_ accumulation. Notably, the monodentate carboxylate groups align in a straight line and face the inside of the pores (Fig. S2c), serving as potential proton conduction sites. Therefore, a unique porous crystalline material with three key elements for CO_2_ reduction was obtained.

Encouraged by the obtained structure, we investigated the catalytic activity of [Fe(H_3_P)]_cryst_ for CO_2_ reduction under visible-light irradiation (400 ≤ *λ* ≤ 750 nm). Before the photocatalysis, [Fe(H_3_P)]_cryst_ was heated at 40 °C under vacuum to remove the crystallization water contained in the framework (Fig. S5). Photocatalysis was performed in a CO_2_-saturated acetonitrile solution using [Fe(H_3_P)]_cryst_ as the heterogeneous catalyst, 1,3-dimethyl-2phenyl-2,3-dihydro-1*H*-benzo[*d*]imidazole (BIH, 0.2 M) as the sacrificial electron donor, tris(2-phenylpyridinato)iridium(iii) (Ir(ppy)_3_, 20 µM) as the photosensitizer, and trifluoroethanol (TFE, 0.2 M) as the proton source. After 3 h of irradiation, the gaseous product was quantified by gas chromatography and the formation of CO (selectivity > 99%, 5600 mmol g^−1^, 89.5 µmol) was confirmed (Table 1, entry 1, see the SI (Fig. S6 and pp. S40–S53) for details). The selectivity for CO was high (>99%), consistent with the previously reported iron porphyrin systems.^80–91^ Note that the corresponding turnover number is 4700 (for 3 h). To verify the role of each component in the photoreaction, we performed a series of control experiments (Table 1, entries 2–7). Trace amounts or no CO formation occurred when the reaction was performed without [Fe(H_3_P)]_cryst_, without BIH, without the photosensitizer, under Ar, or under dark conditions (Table 1, entries 2–6). In the absence of a proton source (entry 7), CO was produced because BIH can also serve as a proton donor,^2^ although the amount of the product was much smaller than that in entry 1. These results confirmed the importance of all components for CO_2_ photoreduction. Here, [Fe(H_3_P)]_cryst_ served as the catalyst, BIH as the sacrificial electron donor, Ir(ppy)_3_ as the photosensitizer, and CO_2_ as the substrate under visible-light irradiation. In addition, TFE served as an external proton source. In addition, we examined the catalytic activity of [Fe(H_3_P)]_cryst_ using tris(2,2′-bipyridine)ruthenium(ii) bis(hexafluoro phosphate) as a photosensitizer, though the amount of CO was smaller (971 mmol g^−1^ for 3 h, 16.5 µmol) than that obtained using Ir(ppy)_3_ as a photosensitizer. We also performed isotopic labeling experiments under a ^13^CO_2_ atmosphere and observed the selective formation of ^13^CO, which indicates that CO is produced from CO_2_ reduction (Fig. S7).

**Table 1:** Control experiments for catalytic CO_2_ reduction by [Fe(H_3_P)]_cryst_ for 3 h^a^

Entry	Catalyst	SED	PS	Gas	Light/nm	Proton source	Products/mmol g^−1^	
							CO	H_2_
1	[Fe(H_3_P)]_cryst_	BIH	Ir(ppy)_3_	CO_2_	400 ≤ *λ* ≤ 750	TFE	5600	0
2	—	BIH	Ir(ppy)_3_	CO_2_	400 ≤ *λ* ≤ 750	TFE	0	0
3	[Fe(H_3_P)]_cryst_	—	Ir(ppy)_3_	CO_2_	400 ≤ *λ* ≤ 750	TFE	0	0
4	[Fe(H_3_P)]_cryst_	BIH	—	CO_2_	400 ≤ *λ* ≤ 750	TFE	1	0
5	[Fe(H_3_P)]_cryst_	BIH	Ir(ppy)_3_	Ar	400 ≤ *λ* ≤ 750	TFE	0	0
6	[Fe(H_3_P)]_cryst_	BIH	Ir(ppy)_3_	CO_2_	Dark	TFE	0	0
7	[Fe(H_3_P)]_cryst_	BIH	Ir(ppy)_3_	CO_2_	400 ≤ *λ* ≤ 750	—	1300	2.9

a Standard conditions: [Fe(H_3_P)]_cryst_ (16 µg), BIH (0.2 M), Ir(ppy)_3_ (20 µM) and TFE (0.2 M) in MeCN.

Subsequently, we examined the robustness of [Fe(H_3_P)]_cryst_. Powder X-ray diffraction (PXRD) patterns of [Fe(H_3_P)]_cryst_, measured before and after photocatalysis, as shown in Fig. S8, are similar, indicating the stability of the framework structure during the reaction. PXRD patterns before and after gas adsorption indicate that [Fe(H_3_P)]_cryst_ is resistant to gas adsorption (Fig. S9). Finally, we examined the heterogeneity of [Fe(H_3_P)]_cryst_. After photocatalysis for 3 h, [Fe(H_3_P)]_cryst_ was removed by filtration. CO_2_ was bubbled through the resulting filtrate and then subjected to another round of photocatalysis. After 3 h of photocatalysis, only trace amounts of CO are detected, indicating that [Fe(H_3_P)]_cryst_ serves as a heterogeneous catalyst (Fig. S10 and Table S4).

The catalytic performance of [Fe(H_3_P)]_cryst_ was compared with those of other porous crystalline solids for photochemical CO_2_ reduction. The average CO production rate of [Fe(H_3_P)]_cryst_ is 1.8 × 10^6^ µmol g^−1^ h^−1^ (selectivity > 99%), which is the highest among previously reported related systems (Table S5), outperforming ZIF-67 (7.5 × 10^5^ µmol g^−1^ h^−1^, selectivity 74%),^20^ Co-MOL@GO (3.5 × 10^5^ µmol g^−1^ h^−1^, selectivity 94%),^35^ and Ru@Cu-HHTP (1.3 × 10^5^ µmol g^−1^ h^−1^, selectivity 93%).^36^ Furthermore, the TON of [Fe(H_3_P)]_cryst_ is the highest among the related systems (Table S5), indicating the efficient utilization of active sites. The catalytic performance was also evaluated in terms of the quantum yield (see the SI (pp. S24–S25, Fig. S11)). The apparent quantum yield^92^ and external quantum efficiency^93^ of [Fe(H_3_P)]_cryst_ were 2.97% and 5.95%, respectively, which fall within the higher range for related systems (Table S6).

**Fig. 2:**
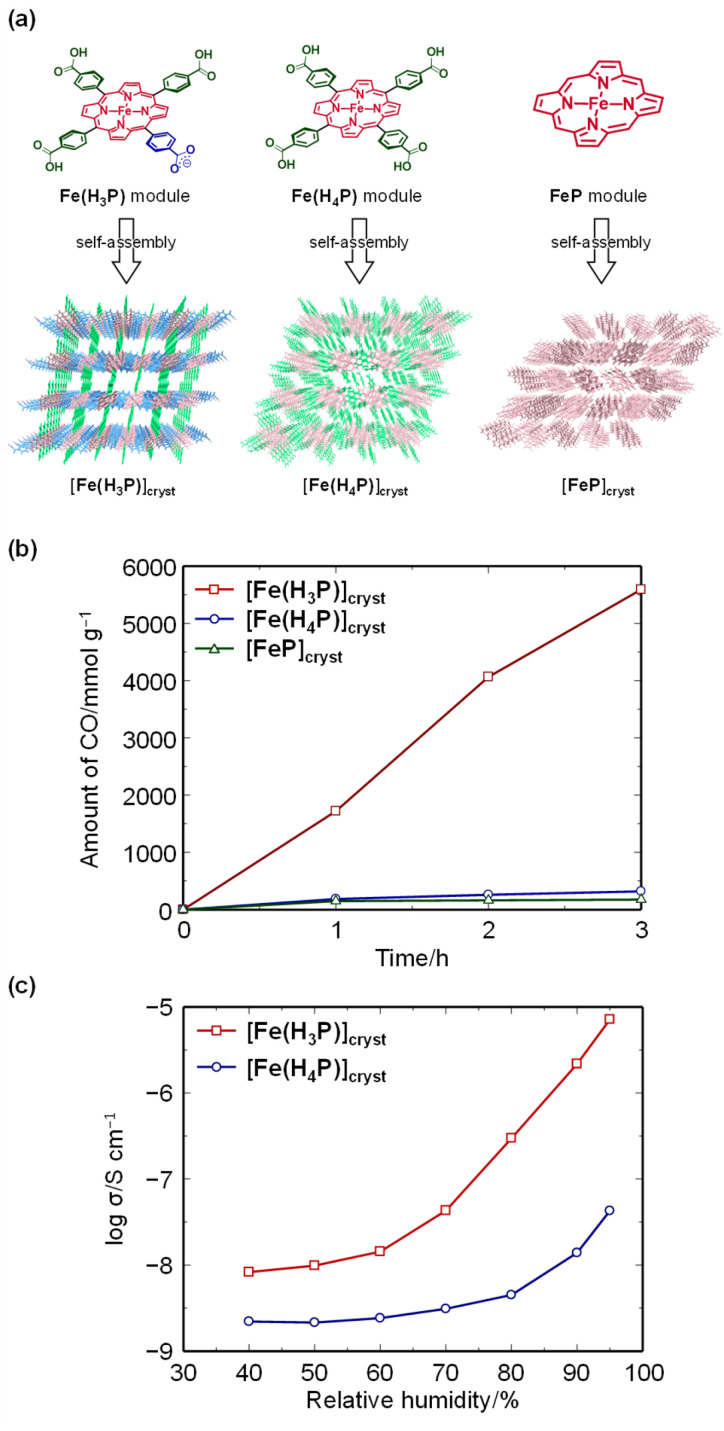
(a) Structural features and (b) time course of CO production by [Fe(H_3_P)]_cryst_, [Fe(H_4_P)]_cryst_, and [FeP]_cryst_. (c) Relative humidity dependence of the proton conductivity of [Fe(H_3_P)]_cryst_ and [Fe(H_4_P)]_cryst_ at 25 °C.

To further examine [Fe(H_3_P)]_cryst_, we conducted several experiments using two molecule-based heterogeneous catalysts containing an iron porphyrin catalytic centre: [FeP]_cryst_ and [Fe(H_4_P)]_cryst_ (Fig. 2a, for details of the structures; see Fig. S12–S15 and Tables S7 and S8).^94^[FeP]_cryst_ lacks a porous structure, whereas [Fe(H_4_P)]_cryst_ is a porous but different structure from [Fe(H_3_P)]_cryst_. As shown in Fig. 2b and Table S9, photocatalysis reveals that their CO_2_ reduction activity follows the order: [FeP]_cryst_ (5.8 × 10^4^ µmol g^−1^ h^−1^) < [Fe(H_4_P)]_cryst_ (1.1 × 10^5^ µmol g^−1^ h^−1^) < [Fe(H_3_P)]_cryst_ (1.8 × 10^6^ µmol g^−1^ h^−1^). A comparison of [FeP]_cryst_ and [Fe(H_4_P)]_cryst_ indicated that the porous structure was effective in enhancing the catalytic activity of the molecule-based heterogeneous catalysts. Furthermore, the CO_2_ reduction activity of [Fe(H_3_P)]_cryst_ was 10 times higher than that of [Fe(H_4_P)]_cryst_, implying that differences in the framework structure influence the CO_2_ reduction activity.

We then measured proton conductivities of [Fe(H_3_P)]_cryst_ and [Fe(H_4_P)]_cryst_. Relative humidity (RH) dependence of proton conductivities at 298 K reveals that the conductivities of [Fe(H_3_P)]_cryst_ are higher than those of [Fe(H_4_P)]_cryst_ at all RH values (Fig. 2c). At 95% RH, the proton conductivity of [Fe(H_3_P)]_cryst_ (7.2 × 10^−6^ S cm^−1^) is 170 times larger than that of [Fe(H_4_P)]_cryst_ (4.3 × 10^−8^ S cm^−1^). We also constructed Arrhenius plots of [Fe(H_3_P)]_cryst_ and [Fe(H_4_P)]_cryst_ by measuring the temperature dependence of their proton conductivities (Fig. S16). Proton conductivities of these materials increased with increasing temperature, and the activation energy of [Fe(H_3_P)]_cryst_, as determined from the slopes of Arrhenius plots, was 0.488 eV, while that of [Fe(H_4_P)]_cryst_ was 0.630 eV. These results indicate that protons are more readily conducted in [Fe(H_3_P)]_cryst_ than in [Fe(H_4_P)]_cryst_.

We further examined these systems by measuring water adsorption and desorption isotherms of [Fe(H_3_P)]_cryst_ and [Fe(H_4_P)]_cryst_ at 298 K and 95% RH. The water uptake of [Fe(H_3_P)]_cryst_ is 2.8 per iron porphyrin module, while that of [Fe(H_4_P)]_cryst_ is 5.6 water molecules (Fig. S17). Despite the high uptake of [Fe(H_4_P)]_cryst_, [Fe(H_3_P)]_cryst_ exhibited higher proton conductivity than [Fe(H_4_P)]_cryst_. We also analysed the crystal structure of [Fe(H_3_P)]_cryst_ immersed in a solvent containing water, [Fe(H_3_P)-H_2_O]_cryst_. The packing structure of [Fe(H_3_P)-H_2_O]_cryst_ reveals that water molecules are aligned along the channel and form a hydrogen network among the monodentate carboxylate groups (Fig. S18 and Table S10). These carboxylate groups in the hydrogen network are the primary driving force for the high proton conductivity.^70–78^ The fact that [Fe(H_3_P)]_cryst_ has much higher activity than [Fe(H_4_P)]_cryst_ even though the CO_2_ adsorption ability of [Fe(H_3_P)]_cryst_ is lower than that of [Fe(H_4_P)]_cryst_ (Fig. S4, *vide supra*) also indicates that the structure of [Fe(H_3_P)]_cryst_ is essential for photochemical CO_2_ reduction. Taken together, these observations suggest that the integration of catalytic centres, porous structures, and proton-conductive features is important for achieving CO_2_ reduction with high performance.

## Conclusions

In conclusion, we have demonstrated a design principle for molecule-based heterogeneous catalysts by crystallographically integrating CO_2_ conversion, CO_2_ accumulation, and proton transport within a single framework. We developed an iron-porphyrin-complex-based framework catalyst, [Fe(H_3_P)]_cryst_, which exhibited high CO_2_ reduction activity under photochemical conditions. This performance is associated with its unique crystalline structure: (i) an iron-porphyrin-complex-based catalytic center, (ii) a crystalline porous structure for CO_2_ accumulation, and (iii) carboxylate groups along the channel. A series of experiments using control materials demonstrated significant differences in catalytic activity among these systems. Owing to the integration of these properties, [Fe(H_3_P)]_cryst_ achieved an average production rate of 1.8 × 10^6^ µmol g^−1^ h^−1^ for CO_2_ to CO conversion (>99% selectivity), which is the highest among related systems. Further investigations of the relationship between the crystal structure and catalytic activity at the molecular level will be important for establishing structure–function relationships in these materials. Our study provides new avenues for designing high-performance molecule-based heterogeneous catalysts.

## Author contributions

X. L. and K. K. contributed equally to this work.

## Conflicts of interest

There are no conflicts to declare.

## Supplementary Material

SC-OLF-D5SC10220K-s001

SC-OLF-D5SC10220K-s002

## Data Availability

The authors have cited additional references within the supplementary information (SI).^95–115^ Supplementary information: general procedures, syntheses, characterization, properties, and crystal structures of catalysts, results of photochemical CO_2_ reduction, and a comparison of catalytic activities are shown in Fig. S1–S18, Tables S1–S10, and Charts 1–29 (PDF). See DOI: https://doi.org/10.1039/d5sc10220k. CCDC 2254373, 2254375, 2500864 and 2495270 contain the supplementary crystallographic data for this paper.^116–119^
