# Supplementary material for: Self-assembly of iron porphyrin complexes bearing carboxyl and carboxylate groups for a highly active CO2 reduction framework catalyst
Source: Chem Sci. 2026 Jul 16;17(31):14901–8. doi: 10.1039/d5sc10220k (PMC13373508; doi:10.1039/d5sc10220k)
Supplement: Supplementary file 1 [file SC-017-D5SC10220K-s001.pdf]

Supplementary Information (SI) for Chemical Science.
This journal is © The Royal Society of Chemistry 2026

Supplementary Information

Self-assembly of iron porphyrin complexes bearing carboxyl and carboxylate groups for highly active CO_2 reduction framework catalyst

Xianjun Li[†], Kento Kosugi[†], Maho Imai, Ritsu Nagai, Zi Lang Goo, Chihoko Fukakusa, Teppei Yamada, Masaki Uchida, Hidehiro Uekusa, Shinpei Kusaka, Ryotaro Matsuda, Yutaka Saga, Tetsuya Kambe, Shigeyuki Masaoka[*], Mio Kondo[*]

[†]These authors contributed equally to this work

[*]Corresponding authors

Table of contents

1. Experimental details **S5**

General procedures **S5**

Synthesis **S5**

Photocatalysis **S7**

$^{13}CO_2$ labeling experiment **S7**

Powder X-ray diffraction measurement **S8**

Single crystal X-ray crystallography **S8**

Calculation of the production rate **S8**

2. Characteristics of the crystal structure of [Fe(H_3P)]$_{cryst}$ **S9**

2.1 Crystallographic data of Fe(H_3P) **S9**

Fig. S1 .... **S9**

Table S1 .... **S10**

2.2 Packing structure of [Fe(H_3P)]$_{cryst}$ **S11**

Fig. S2 .... **S11**

2.3 Protonation state **S12**

Fig. S3 .... **S13**

Table S2 .... **S13**

3. Gas adsorption measurement **S14**

Fig. S4 .... **S14**

Table S3 .... **S15**

4. Thermogravimetric analysis **S16**

Fig. S5 .... **S16**

4. GC calibration curve **S17**

Fig. S6 .... **S17**

4. Isotopic labeling experiments **S18**

Fig. S7 .... **S18**

5. PXRD measurements **S19**

Fig. S8 .... **S19**

Fig. S9 .... **S19**

6. Photocatalysis of the filtrate **S20**

Fig. S10 .... **S20**

Table S4 .... **S20**

7 Performance of porous crystalline solids **S21**

Table S5 .... **S21**

8. Quantum yield **S24**

8.1 Determination of the light intensity **S24**

Fig. S11 .... **S25**

8.2 Comparison of the quantum yield **S26**

Table S6 .... **S26**

9. Characteristics of the crystal structure of $[FeP]_{cryst}$ and $[Fe(H_4P)]_{cryst}$ **S27**

9.1 Crystallographic data of FeP **S27**

Fig. S12 .... **S27**

Table S7 .... **S28**

9.2 Packing structure of $[FeP]_{cryst}$ **S29**

Fig. S13 .... **S29**

9.3 Crystallographic data of $[Fe(H_4P)]_{cryst}$ **S30**

Fig. S14 .... **S30**

Table S8 .... **S31**

9.4 Packing structure of $[Fe(H_4P)]_{cryst}$ **S32**

Fig. S15. .... **S32**

10. Photocatalysis of $[Fe(H_3P)]_{cryst}$, $[Fe(H_4P)]_{cryst}$ and $[FeP]_{cryst}$ **S33**

Table S9 .... **S33**

11. Proton conductivity measurements **S34**

Fig. S16 .... **S34**

12. Water adsorption measurements **S35**

Fig. S17 .... **S35**

13. Characteristics of the crystal structure of [Fe(H_3P)-H_2O]$_{cryst}$ **S36**

13.1 Crystallographic data of Fe(H_3P)-H_2O **S36**

Table S10 .... **S36**

13.2 Packing structure of [Fe(H_3P)-H_2O]$_{cryst}$ **S37**

Fig. S18 .... **S38**

14. Chromatograph data **S39**

15. MALDI-TOF-MS **S53**

16. NMR chart **S54**

1. Experimental details

General procedures

2-phenylbenzimidazole, Methyl iodide, 2,2,2-Trifluoroethanol (TFE) were purchased from Tokyo Chemical Industry Co., Ltd. Methanol (MeOH), ethanol (EtOH), chloroform ($CHCl_3$), *N*, *N*-dimethylformamide (DMF), tetrahydrofuran (THF), hexane and acetonitrile (MeCN) were purchased from Kanto Chemical Co., Inc. Potassium hydroxide (KOH), hydrochloric acid (HCl), methyl *p*-formylbenzoate, acetic acid (AcOH) and tris(2-phenylpyridine)iridium ($Ir(ppy)_3$) were purchased from FUJIFILM Wako Pure Chemical Corporation. Sodium borohydride ($NaBH_4$) and pyrrole were purchased from Sigma-Aldrich Co. LLC. Methyl *p*-formylbenzoate and anhydrous iron(III) chloride ($FeCl_3$) were purchased from Kishida Chemical Co., LLC. Chloroform-d_1 ($CDCl_3$) and barium carbonate-^{13}C ($Ba^{13}CO_3$) were purchased from Cambridge Isotopes, Inc. Argon (Ar) was purchased from Kansai Gas First Co., Ltd. Carbon dioxide (CO_2) was purchased from Kinki Sanso Co., Ltd. Helium (He) and nitrogen (N_2) were purchased from Iwatani Corporation. Pure water was purified using the Millipore MiliQ purifier. ^1H-NMR spectra were collected at room temperature on a JEOL JNM-ECS400 spectrometer. UV-visible absorption spectra were recorded on a Shimadzu UV-3600 UV-vis-near-IR spectrophotometer. Elemental analysis was performed on a J-SCIENCE LAB MICRO CORDER JM10 elemental analyzer. The adsorption isotherm of CO_2 at 195 K was measured using a BELSORP-max volumetric adsorption analyzer from Microtrac-BEL corp. The adsorption isotherm of H_2O at 298 K was measured using BELSORP-max II volumetric adsorption analyzer from MICROTRAC MRB. Powder X-ray diffraction (PXRD) patterns were collected on MiniFlex600/DTEX (Rigaku) at 293K, being processed through PDXL2 software. Simulated PXRD patterns were generated from single-crystal X-ray structures using Mercury 3.3.

Synthesis

Synthesis of 5,10,15,20-tetrakis(4-methoxycarbonylphenyl)porphyrin (Me$_4$P)

Me$_4$P was prepared by a modification of a reported procedure.[90] In a round-bottom flask, methyl *p*-formylbenzoate (1999.3 mg, 12.2 mmol) was dissolved in propanoic acid (29 mL). Then, pyrrole (0.87 mL, 2.5 mmol) was added dropwise, followed by reflux overnight in darkness. After cooling down to room temperature, purple precipitates were collected through membrane filtration, washing with methanol. The obtained solid was dried in a vacuum oven for 2 hours at 40 °C, giving **Me$_4$P** in 77% yield (1985.3 mg). ^1H NMR (400 MHz, $CDCl_3$): δ = 8.82 (s, 8H), 8.44-8.46 (d, 8H), 8.28-8.30 (d, 8H), 4.11(s, 12H), −2.82 (s, 2H). Elemental analysis Calcd. for **Me$_4$P**·0.75 H_2O: C, 72.59%; H, 4.63%; N, 6.51%. Found: C, 72.40%; H, 4.37%; N, 6.38%.

Synthesis of iron(III) 5,10,15,20-tetrakis(4-methoxycarbonylphenyl)porphyrin chloride (Fe(Me$_4$P))

Fe(Me$_4$P) was prepared by a modification of a reported procedure.[90] **Me$_4$P** (264.1 mg, 0.3 mmol) and anhydrous $FeCl_3$ (186.0 mg, 1.2 mmol) were mixed in DMF (20 mL) then heated in a microwave reactor

for 1 h at 200 °C after 20 minutes pre-stirring. Subsequently, diluted HCl (1 M, 80 mL) was added to the resulting mixture. Dark brown precipitates were obtained by filtration and washed twice with 1 M HCl (20 mL). The obtained solid was dried in a vacuum oven for 1.5 hours at 100 °C, giving **Fe(Me$_4$P)** as a brown solid in 90% yield (264.1 mg). Elemental analysis Calcd. for **Fe(Me$_4$P)**·0.55 H_2O·0.55 DMF: C, 65.33%; H, 4.18%; N, 6.46%. Found: C, 65.31%; H, 4.13%; N, 6.47%.

Synthesis of iron(III) 5,10,15,20-tetrakis(4-carboxyphenyl)porphyrin chloride (Fe(H$_4$P))

Fe(H$_4$P) was prepared by a modification of a reported procedure.[90] **Fe(Me$_4$P)** (368.9 mg, 0.4 mmol) and KOH (1347.8 mg, 24 mmol) were dissolved in a mixed solvent of MeOH (12.5 mL), THF (12.5 mL) and water (12.5 mL). After heating up the mixture at 66 °C overnight, THF and MeOH were removed by evaporation. Excess hot water was used to dissolve all the solids, and the resulting solution was acidified with 1 M HCl until no further precipitate formed. The precipitate was recrystallized from acetone and hexane, giving **Fe(H$_4$P)** as a brown solid (287.2 mg, yield 83%). Elemental analysis Calcd. for **Fe(H$_4$P)**·0.6 H_2O: C, 64.71%; H, 3.30%; N, 6.29%. Found: C, 64.90%; H, 3.51%; N, 6.41%.

Synthesis of [Fe(H$_3$P)]$_{cryst}$ framework

To the solution of **Fe(H$_4$P)** (58.4 mg, 0.066 mmol) in MeOH (50 mL), KOH (143.6 mg, 2.56 mmol) was added at room temperature. After 10 minutes of sonication, the solution color completely changed from dark reddish-brown to dark green. Then the pH of the solution was adjusted to around 3-5 by acetic acid, heating up overnight at 50 ℃ in an oven. Dark brown needle-like crystals were obtained by membrane filtration, followed by washing with ethyl acetate until the filtrate became colorless (37 mg, 63% yield). Elemental analysis Calcd. for **Fe(H$_3$P)**·3.6 H_2O: C, 63.46%; H, 3.79%; N, 6.17%. Found: C, 63.17%; H, 3.48%; N, 6.36%.

Synthesis of [Fe(H$_4$P)]$_{cryst}$ framework

[**Fe(H$_4$P)**]$_{cryst}$ was prepared by recrystallization. **Fe(H$_4$P)** (49.8 mg) was dissolved in 10 mL AcOH. Then, 0.3 mL of 0.05 wt% KOH aqueous solution was added. The mixture was heated in a Teflon-lined stainless-steel autoclave to 180 °C for 3 h and then maintained at 60 ℃ overnight. After drying up in a 60 °C vacuum oven for 2 h, **[Fe(H$_4$P)]$_{cryst}$** was obtained as a dark brown crystal (39.6 mg, yield 80%).

Synthesis of iron(III) porphine (FeP)

Porphine was synthesized by the modification of a previous report.[95] Formaldehyde (370 µL, 5.0 mmol, 37% in methanol) was dissolved in a mixed solvent of $CHCl_3$ (500 mL) and EtOH (7 mL), then stirred for 30 min under N_2 atmosphere. After pyrrole (0.35 mL, 5.0 mmol) was added, boron trifluoride diethyl etherate (BF_3·OEt_2) (0.23 mL, 1.7 mmol) was added to the reaction mixture, and the mixture was stirred for 17 h at room temperature in the dark. Subsequently, a solution of DDQ (0.9 g, 4.0 mmol) in toluene (20

mL) was added, and the mixture was refluxed. After 2 h, the reaction mixture was quenched by TEA (0.20 mL, 1.4 mmol). The resulting mixture was purified by silica gel column chromatography ($CHCl_3$ as eluent) to afford a dark purple solution. Recrystallization from DCM/MeOH gave a purple crystal of porphine (10.6 mg, yield 3%). Insertion of an iron ion into porphine was carried out as follows. A solution of DMF (5.0 mL) containing porphine (9.6 mg, 0.031 mmol) and $FeCl_3 \cdot 4H_2O$ (15.6 mg, 0.078 mmol) was heated at 160 °C and stirred for 5.5 h under an Ar atmosphere. Diluted HCl (0.2 M) was added to the resulting solution. Precipitate was collected by filtration and washed with 0.2 M HCl. Recrystallization from slow evaporation of a mixed solvent of $CHCl_3$ and acetone gave a dark brown crystal, **[FeP]$_{cryst}$**, suitable for single crystal X-ray analysis (6.5 mg, yield 52%).

Synthesis of 1,3-dimethyl-2-phenyl-2,3-dihydro-1*H*-benzo[d]imidazole (BIH)

BIH was prepared by a modification of a reported procedure.[96] In a two-necked flask, 2-phenylbenzimidazole (4998.8 mg, 25.8 mmol) and potassium hydroxide (2227.0 mg, 39.7 mmol) were dissolved in methanol (100 mL) under an argon atmosphere. Methyl iodide (4.9 mL, 78.7 mmol) was added in the mixture and heated up to 110 °C for 3 days. After cooling down to room temperature, excess water was added in the solution until no further white precipitates were formed. The crude intermediate was collected by filtration, washing with water multiple times. The intermediate was dissolved in 80 mL of methanol and cooled in an ice bath, and then $NaBH_4$ (5914.7 mg, 156.3 mmol) was added slowly. After stirring for 1 h at room temperature, excess water was added. The white solid was collected by membrane filtration. Needlelike crystal of BIH was obtained by thermal recrystallization from ethanol/water (5/1) upon slow cooling from 95 °C to room temperature (1801 mg, yield 31%). ^1H NMR (400 MHz, $CDCl_3$): δ = 7.55 (d, 2H), 7.44 (d, 3H), 6.61 (m, 2H), 6.46 (m, 2H), 4.86 (s, 1H), 2.47 (s, 6H).

Photocatalysis

For a typical run, 16 μg of crystal of **[Fe(H$_3$P)]$_{cryst}$** was suspended in a MeCN (2.0 mL) solution containing 0.20 M BIH, 0.20 M TFE and 20 μM Ir(ppy)$_3$. The solution was sonicated for 15 minutes unless otherwise stated. Subsequently, CO_2 was purged for 10 minutes. The solution was then irradiated with a 300 W Xe lamp equipped with a 400 nm long pass filter (Edmund Industrial Optics) to produce the light in the range of $400 \leq \lambda \leq 750$ nm at 20 ºC in a custom made aluminium box with a cooling system. The amount of CO and H_2 produced at the headspace of the cell was quantified by a Shimadzu GC-8A with a TCD detector equipped with a packed column with Molecular Sieve 13X-S 60/80. Calibration curves were obtained by sampling known amounts of H_2 and CO.

$^{13}CO_2$ labeling experiment

A solution of MeCN (2.0 ml) containing 35 μg **[Fe(H$_3$P)]$_{cryst}$** suspension, 0.20 M BIH**,** 0.20 M TFE and 20 μM Ir(ppy)$_3$ was purged with Ar for 15 min, followed by $^{13}CO_2$ bubbling for 5 min. The $^{13}CO_2$ gas was

produced by adding 2.0 M HCl to solid $Ba^{13}CO_3$ (98 atom % ^{13}C, Sigma Aldrich). The solution was then irradiated for 3 h with a 300 W Xe lamp equipped with a 400 nm long pass filter (Edmund Industrial Optics) to produce the light in the range of $400 \leq \lambda \leq 750$ nm at 20 ºC in a custom made aluminium box with a cooling system. The evolved CO was detected by a GCMS-QP2020 (Rt®-Msieve 5A (30 m, 0.53 mm ID, 50 µm df) He carrier gas, 40 °C).

Powder X-ray diffraction measurement

Powder X-ray diffraction data before and after photocatalysis were collected on a Rigaku MiniFlex600. Photocatalysis was performed using CO_2 saturated MeCN solution containing 3.1 mg **[Fe(H$_3$P)]$_{cryst}$** suspension, 0.20 M BIH, 0.20 M TFE and 20 µM Ir(ppy)$_3$ at 20 ºC upon irradiation with Xe lamp ($400 \leq \lambda \leq 750$ nm) for 3 h.

Single crystal X-ray crystallography

Single crystal X-ray diffraction data of **[Fe(H$_3$P)]$_{cryst}$**, **[Fe(H$_4$P)]$_{cryst}$**, and **[FeP]$_{cryst}$** were collected on a Synergy Custom system CCD Plate equipped with confocal monochromated Mo-Kα radiation ($\lambda = 0.71069$ Å) , and that of **[Fe(H$_3$P)-H$_2$O]$_{cryst}$** were collected on a diffractometer equipped with a beamline BL-5A at KEK (the High Energy Accelerator Research Organization, Japan) with a Pilatus3 S6M detector (synchrotron, $\lambda = 0.7500$ Å, T = 100 K). In both cases, crystals were coated with Paratone-N (Hampton Research Corp., Aliso Viejo, CA, USA). Data was processed using CrysAlisPro system software.[97] The structure was solved by dual-space algorithm using SHELXT program[98] through the Olex2 interface.[99] All non-hydrogen atoms were refined anisotropically using a least-squares method, and hydrogen atoms were fixed at calculated positions and refined using a riding model. SHELXL−2014/7 was used for structure refinement.[100] Full-matrix least-squares refinements on F^2 based on unique reflections with unweighted and weighted agreement factors of $R = \Sigma||F_o| - |F_c||/\Sigma|F_o|$ ($I > 2.00\ \sigma(I)$) and $wR = [\Sigma w(F_o^2 - F_c^2)^2/\Sigma w(F_o^2)^2]^{1/2}$ were performed. Mercury 4.0.0 was used for visualization and analysis of the structure. Crystallographic data have been deposited with Cambridge Crystallographic Data Centre: Deposition numbers CCDC 2254373, 2254375, 2500864, and 2495270 for **[Fe(H$_3$P)]$_{cryst}$**, **[Fe(H$_4$P)]$_{cryst}$**, **[Fe(H$_3$P)-H$_2$O]$_{cryst}$**, and **[FeP]$_{cryst}$**, respectively. Copies of the data can be obtained free of charge via www.ccdc.cam.ac.uk/data_request/cif.

Calculation of the production rate

Production rates for CO and H_2 were evaluated by

$$\text{Production rate } [\mu\text{mol g}^{-1}\text{ h}^{-1}] = \frac{\text{amount of product } [\mu\text{mol}]}{\text{amount of catalyst } [\text{g}] \times \text{time } [\text{h}]} \quad \text{(S1)}$$

2. Characteristics of the crystal structure of $[Fe(H_3P)]_{cryst}$

2.1 Crystallographic data of $Fe(H_3P)$

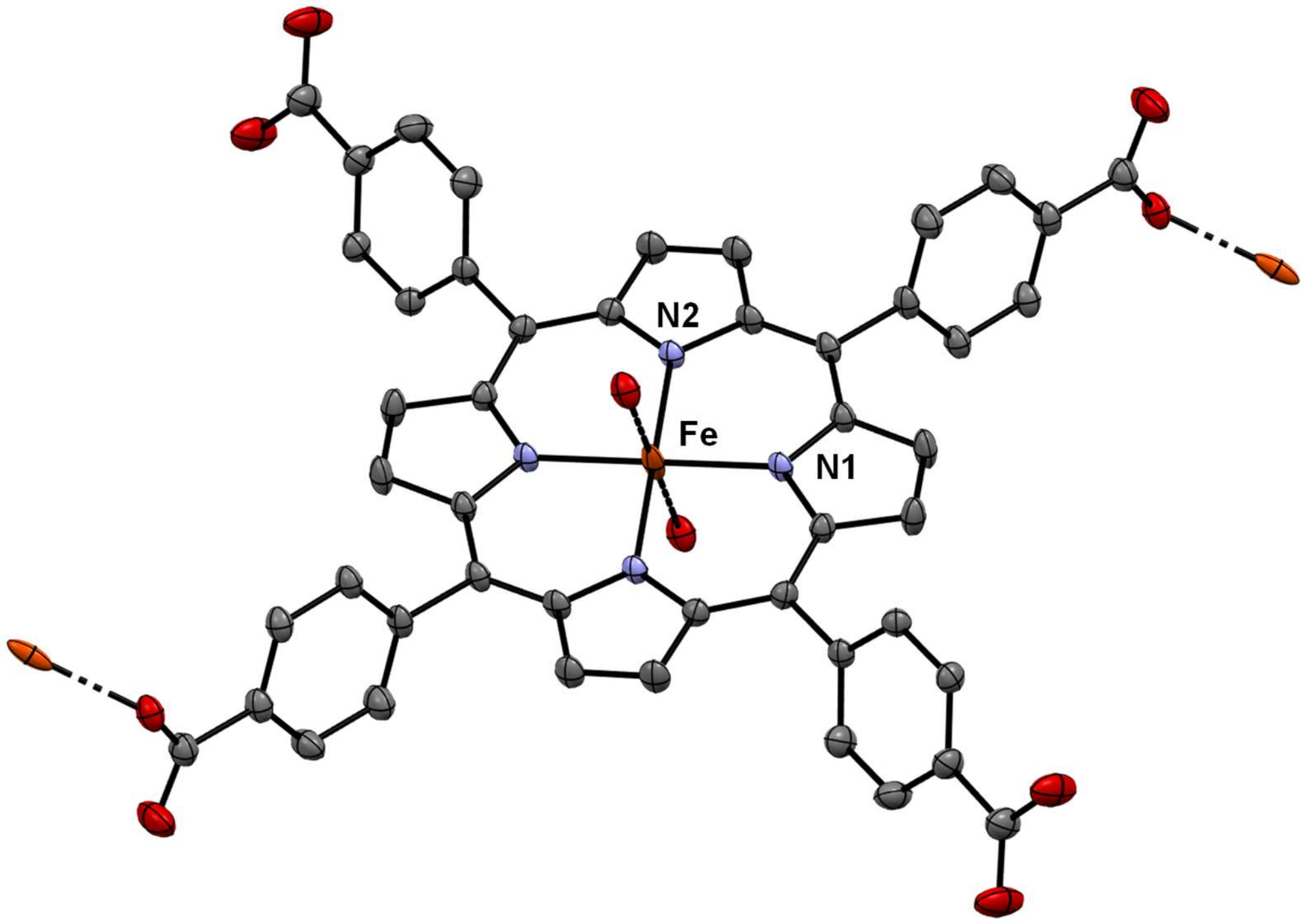

Fig. S1. ORTEP drawings of **$Fe(H_3P)$** (50% probability ellipsoids). Hydrogen atoms are omitted for clarity. C = gray, N = blue, O = red, and Fe = orange.

Table S1. Summary of crystallographic data for **Fe(H_3P)**.

	**Fe(H_3P)**
Formula	$C_{48}H_{27}FeN_4O_8$
Fw	843.59
Crystal color, habit	Dark brown, block
Crystal size / mm^3	0.24 × 0.05× 0.05
Crystal system	Monoclinic
Space group	P 2_1/c
a / Å	15.4700(6)
b / Å	8.0358(3)
c / Å	20.9396(7)
α / °	90
β / °	101.340(4)
γ / °	90
V / Å^3	2552.26(17)
Z	2
F(000)	864.0
d_{calc} / g cm^{-3}	1.096
μ(MoKa) / mm^{-1}	0.710
T / K	123(2)
R_1	0.0535
wR_2	0.1533
GooF	1.036

2.2 Packing structure of $[Fe(H_3P)]_{cryst}$

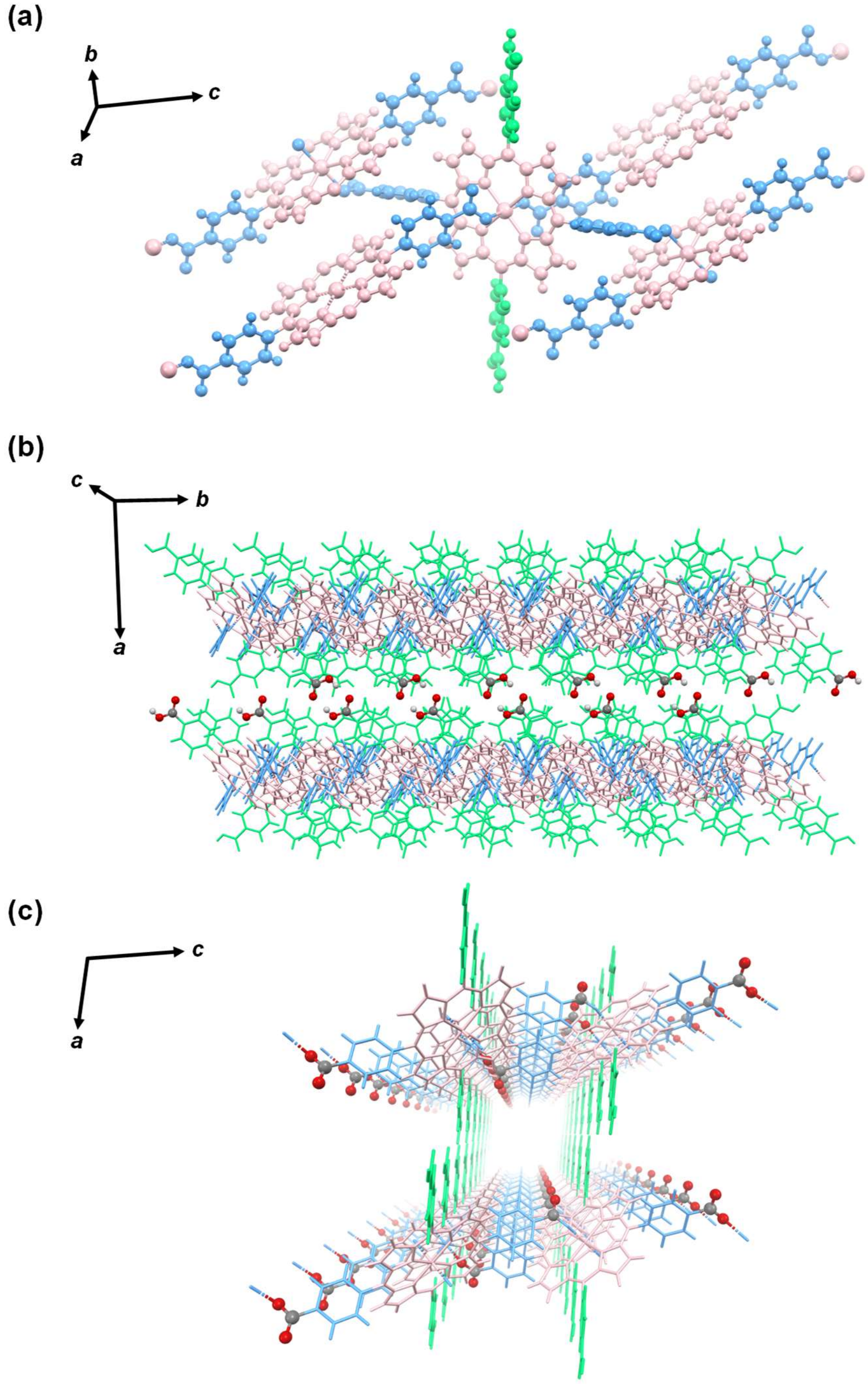

Fig. S2. (a) Coordination of the carboxylate groups of **$Fe(H_3P)$** to iron centers of adjacent iron porphyrin modules. For clarity, carboxyl groups of adjacent modules are omitted. (b) Hydrogen bonding interactions between carboxylic acid groups across the 2D layers. For clarity, the frontmost carboxylic groups are represented as ball and stick. (c) Carboxylate groups align in a straight line and face inside the pores. For clarity, carboxylate groups are represented as ball and stick.

2.3 Protonation state

The protonation state of **[Fe(H$_3$P)]$_{cryst}$** was confirmed based on the single-crystal X-ray structure of **[Fe(H$_3$P)]**$_{cryst}$. From the average distance between an iron centre and nitrogen atoms of the porphyrin core (2.046 Å), the oxidation state of iron centre is determined to be Fe(III).[101] Given that neither counter cations nor anions were observed in the crystal structure, the molecule in the structure should be neutral. As the charge of Fe(III)-porphyrin core is +1, one carboxylic acid group must be deprotonated and coordinated to Fe(III) centre to maintain overall charge neutrality, leading to the assignment of **Fe(H$_3$P)** (Fig. S3). It should be noted that the composition of **[Fe(H$_3$P)]$_{cryst}$** was also confirmed by elemental analysis (p. S6 in the SI).

We also examined the pK_a of **Fe(H$_3$P)**. However, its low solubility in aqueous media prevented reliable experimental determination. In addition, iron porphyrin complexes are known to form *μ*-oxo dimers under basic conditions[102] making it difficult to define a single species over a wide pH range. As an alternative approach, we performed quantum chemical calculations to evaluate the enthalpy change (ΔH) associated with the deprotonation process. Quantum chemical calculations were performed on **Fe(H$_n$P)** (n = 0, 1, 2, 3, 4 for high spin state of iron center) using density functional theory (DFT). All calculations were performed with the ORCA 6.1.1 program package[103]. Geometries were optimized with DFT, where hybrid meta-GGA TPSSh functionals was used for the exchange-correlation term[104]. As a basis set, def2-svp was used and the resolution-of-identity (RI) approximation was applied using the def2/J auxiliary basis set. Based on the experimental conditions, PCM-SMD solvation model was used to implicitly include the effect of methanol. Vibrational analyses confirmed the absence of imaginary frequencies. Note that Gibbs free energy is not rational to use in the ordinary framework of PCM because entropy term is treated in ideal gas[105]. The results are summarized in Table S2. The ΔH are slightly high (~30 kcal/mol), suggesting that simple deprotonation is thermodynamically unfavored in solution. Nevertheless, we could selectively obtain **[Fe(H$_3$P)]$_{cryst}$** as a single product. These facts indicate that **[Fe(H$_3$P)]$_{cryst}$** is thermodynamically stable crystalline phase maybe due to its neutral and infinite three-dimensional network structure stabilized by coordination bonds and hydrogen bonds.

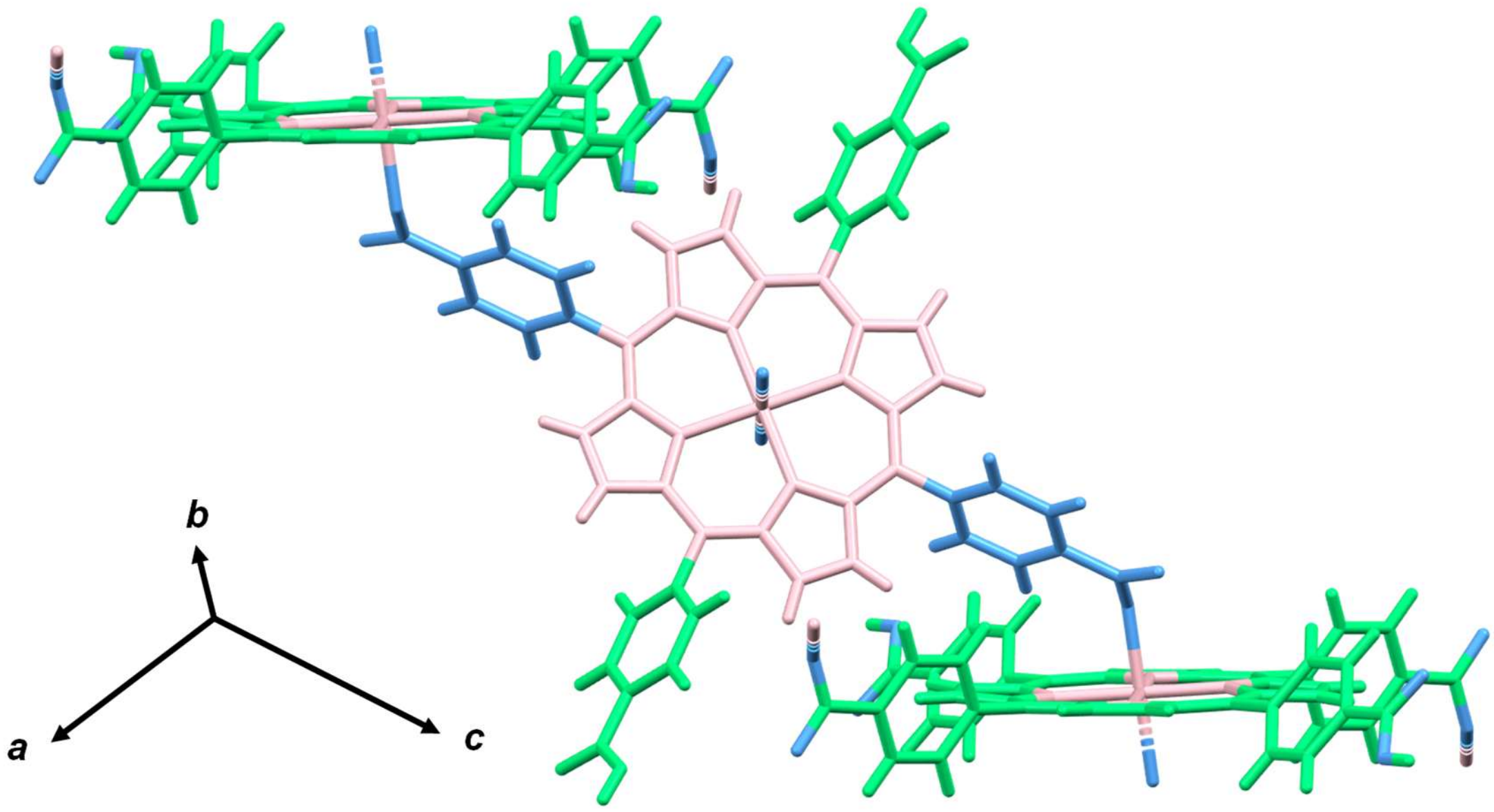

Fig. S3. Coordination of the carboxylate group to the Fe(III) center in the crystal structure of **[Fe(H3P)]cryst**.

Table S2. Enthalpies for the deprotonation reactions.

Reactions	ΔH/kcal mol^{-1}	ΔG[a]/kcal mol^{-1}
$Fe(H_4P) + H_2O \rightarrow Fe(H_3P) + H_3O^+$	31.4	31.3
$Fe(H_3P) + H_2O \rightarrow Fe(H_2P) + H_3O^+$	28.9	26.9
$Fe(H_2P) + H_2O \rightarrow Fe(H_1P) + H_3O^+$	29.4	28.7
$Fe(H_1P) + H_2O \rightarrow Fe(H_0P) + H_3O^+$	33.7	33.2

[a] Gibbs free energies are shown for reference only and should be interpreted with caution, since entropy terms in the conventional PCM framework are evaluated using the ideal-gas approximation.[105]

3. Gas adsorption measurement

Fig. S4 shows the results of gas adsorption measurement of **[Fe(H_3P)]$_{cryst}$**, **Fe(H_4P)]$_{cryst}$**, and the crystal of iron(III) tetraphenylporphyrin chloride, **[FeTPPCl]$_{cryst}$**. Table S3 shows the comparison of CO_2 adsorption ability of **[Fe(H_3P)]$_{cryst}$** with relevant systems. Although the CO_2 adsorption ability of **[Fe(H_3P)]$_{cryst}$** is lower than that of **[Fe(H_4P)]$_{cryst}$**, it remains clearly distinguishable from that of nonporous molecular crystal **[FeTPPCl]$_{cryst}$**, which shows negligible CO_2 uptake under the similar conditions (orange line). These observations together with our investigations on catalytic activity clearly indicate the following points. First, the existence of porous structures is essential for obtaining high catalytic activity as non-porous **[FeP]$_{cryst}$** exhibited significantly lower activity than porous structures, **[Fe(H_3P)]$_{cryst}$** and **[Fe(H_4P)]$_{cryst}$**. Second, the CO_2 adsorption ability is not sole factor to determine catalytic activity because **[Fe(H_3P)]$_{cryst}$**, which has lower CO_2 absorption ability than **[Fe(H_4P)]$_{cryst}$**, exhibited better catalytic performance than **[Fe(H_4P)]$_{cryst}$**. These considerations suggest that the structure of **[Fe(H_3P)]$_{cryst}$** is crucial for obtaining efficient catalyst for photochemical CO_2 reduction.

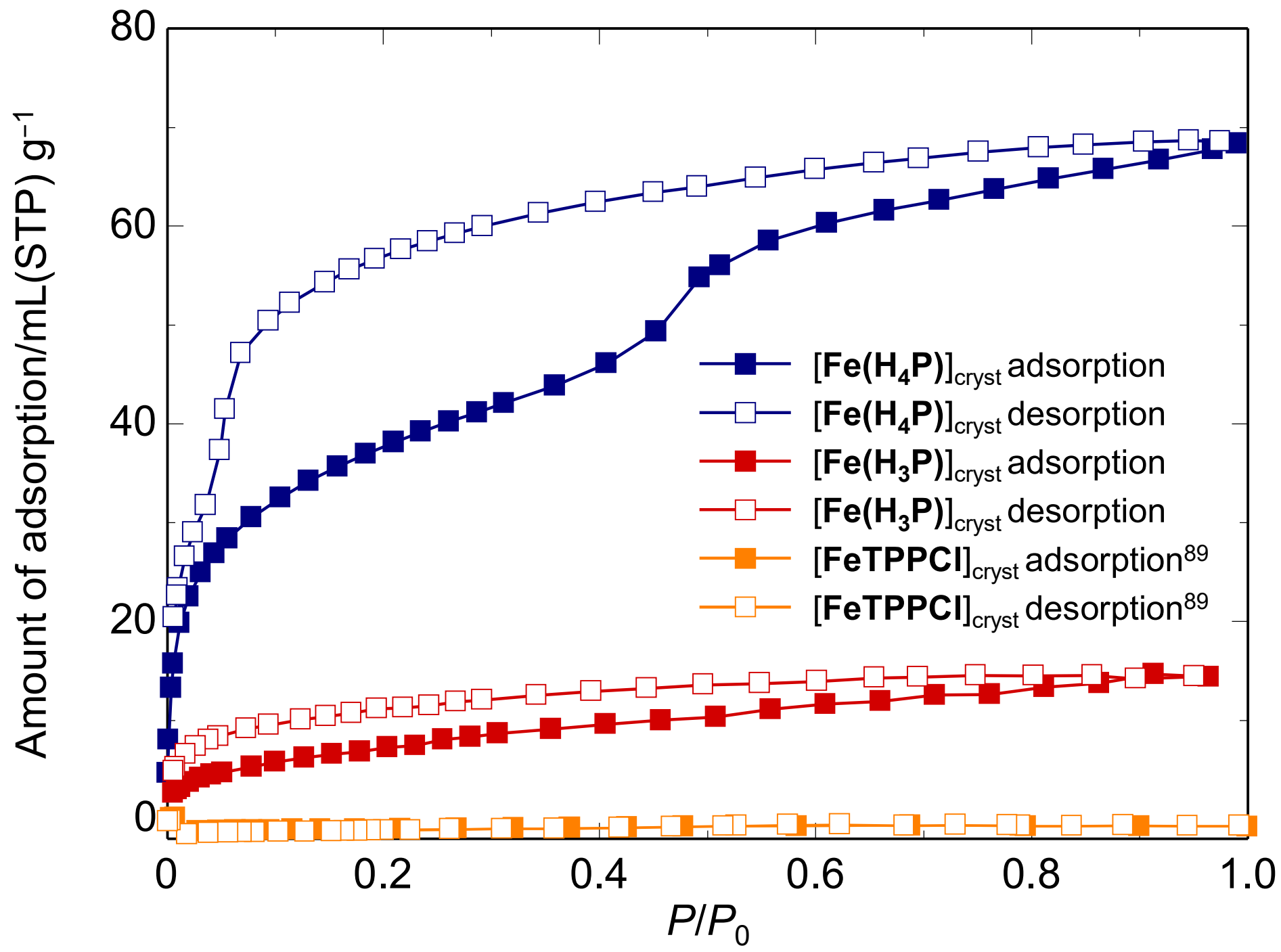

Fig. S4. Adsorption and desorption isotherms of CO_2 for **[Fe(H_3P)]$_{cryst}$** (blue), **[Fe(H_4P)]$_{cryst}$** (red) and **[FeTPPCl]$_{cryst}$** (orange) at 195 K.

Table S3. Comparison of CO_2 uptakes of the porphyrin-based framework systems.

Frameworks	CO_2 uptake/mmol g^{-1}	Ref.
**$[Fe(H_3P)]_{cryst}$**	0.65	This work
**$[Fe(H_4P)]_{cryst}$**	**3.06**	**This work**
MOF-525	1.12	106
MOF-525-Zn	1.25	106
MOF-525-Co	1.50	106
PCN-222	2.60	107

4. Thermogravimetric analysis

Prior to the catalysis, **[Fe(H_3P)]$_{cryst}$** was heated under vacuum to remove the solvents. Indeed, the thermogravimetric analysis (TGA) data of dried crystals of **[Fe(H_3P)]$_{cryst}$** indicates that the crystalline solvent has been completely removed from the pores (**Fig. S5**).

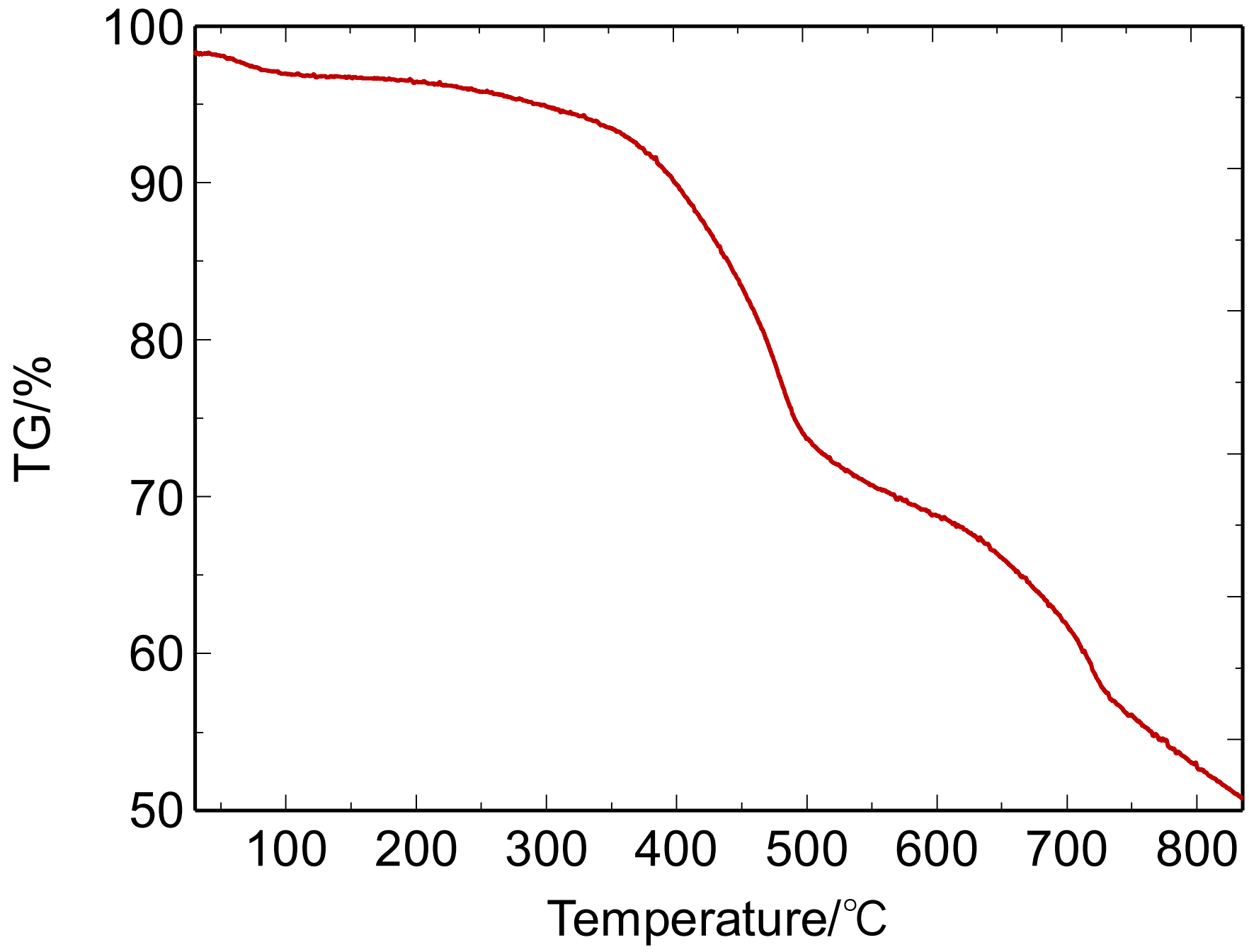

Fig. S5. Thermogravimetric curve of **[Fe(H_3P)]$_{cryst}$** under N_2.

4. GC calibration curve

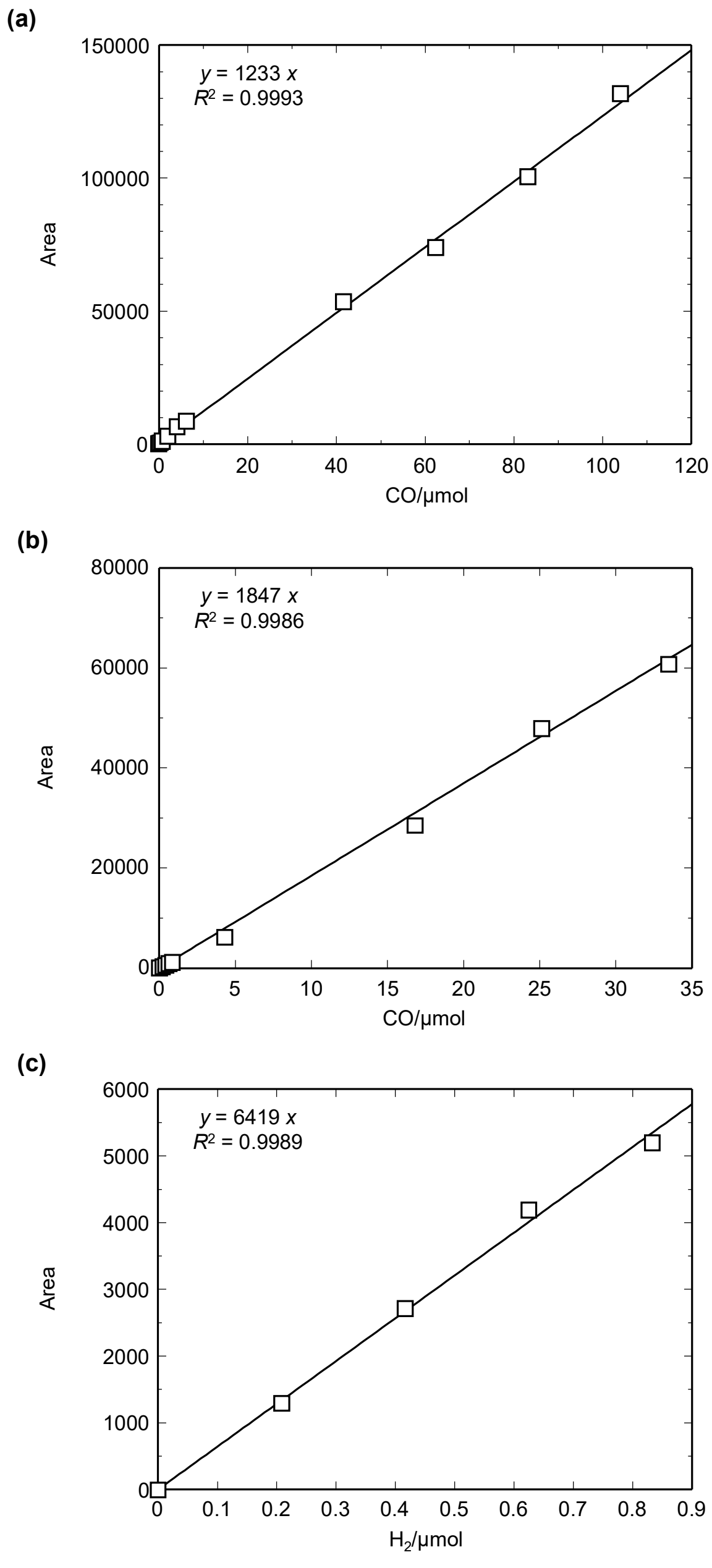

Fig. S6. GC calibration curves for the quantification of (a) CO in photochemical CO_2 reduction for **[Fe(H$_3$P)]$_{cryst}$** (except for the quantification to determine quantum yield) and **[Fe(H$_4$P)]$_{cryst}$**, (b) CO in photochemical CO_2 reduction for **[FeP]$_{cryst}$** and quantum yield determination of **[Fe(H$_3$P)]$_{cryst}$**, and (c) H_2 in photochemical CO_2 reduction.

4. Isotopic labeling experiments

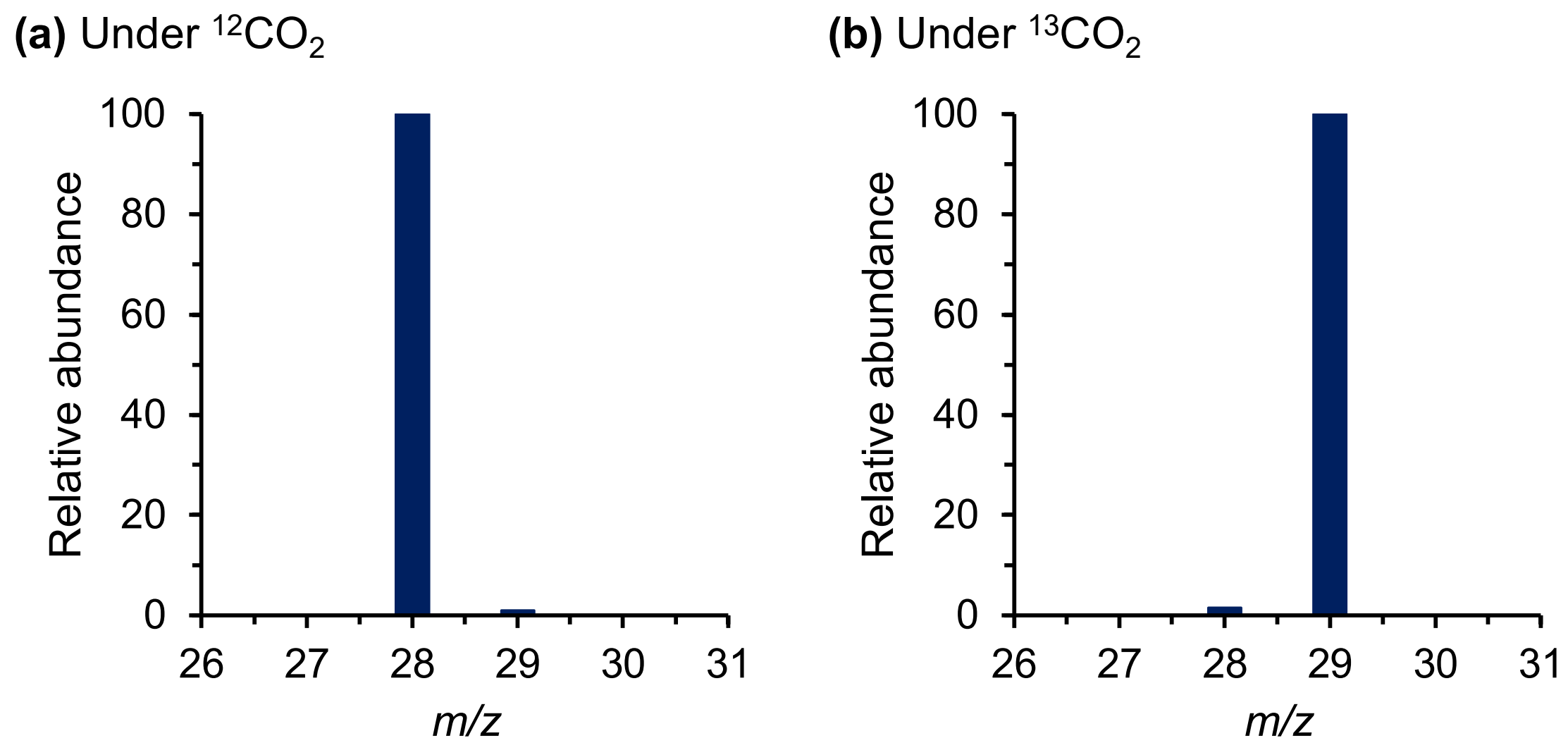

Fig. S7. Mass spectra of CO generated using MeCN solution containing **[Fe(H$_3$P)]$_{cryst}$**, Ir(ppy)$_3$, BIH and TFE at 20 °C upon irradiation with a Xe lamp ($400 \leq \lambda \leq 750$ nm) under (a) $^{12}CO_2$ and (b) $^{13}CO_2$ for 3 h.

5. PXRD measurements

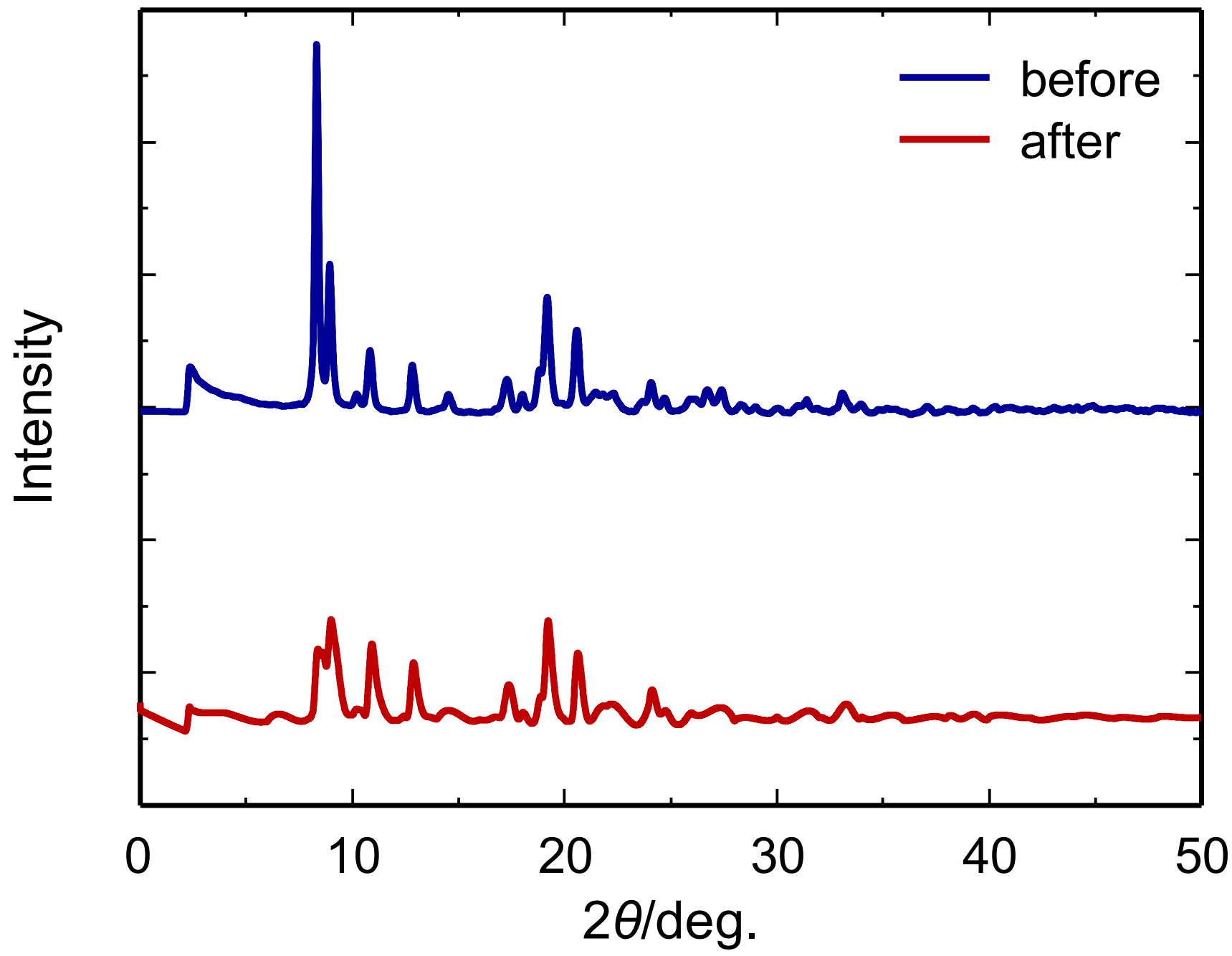

Fig. S8. PXRD patterns of **[Fe(H3P)]**$_{cryst}$ before (blue) and after (red) the photocatalysis.

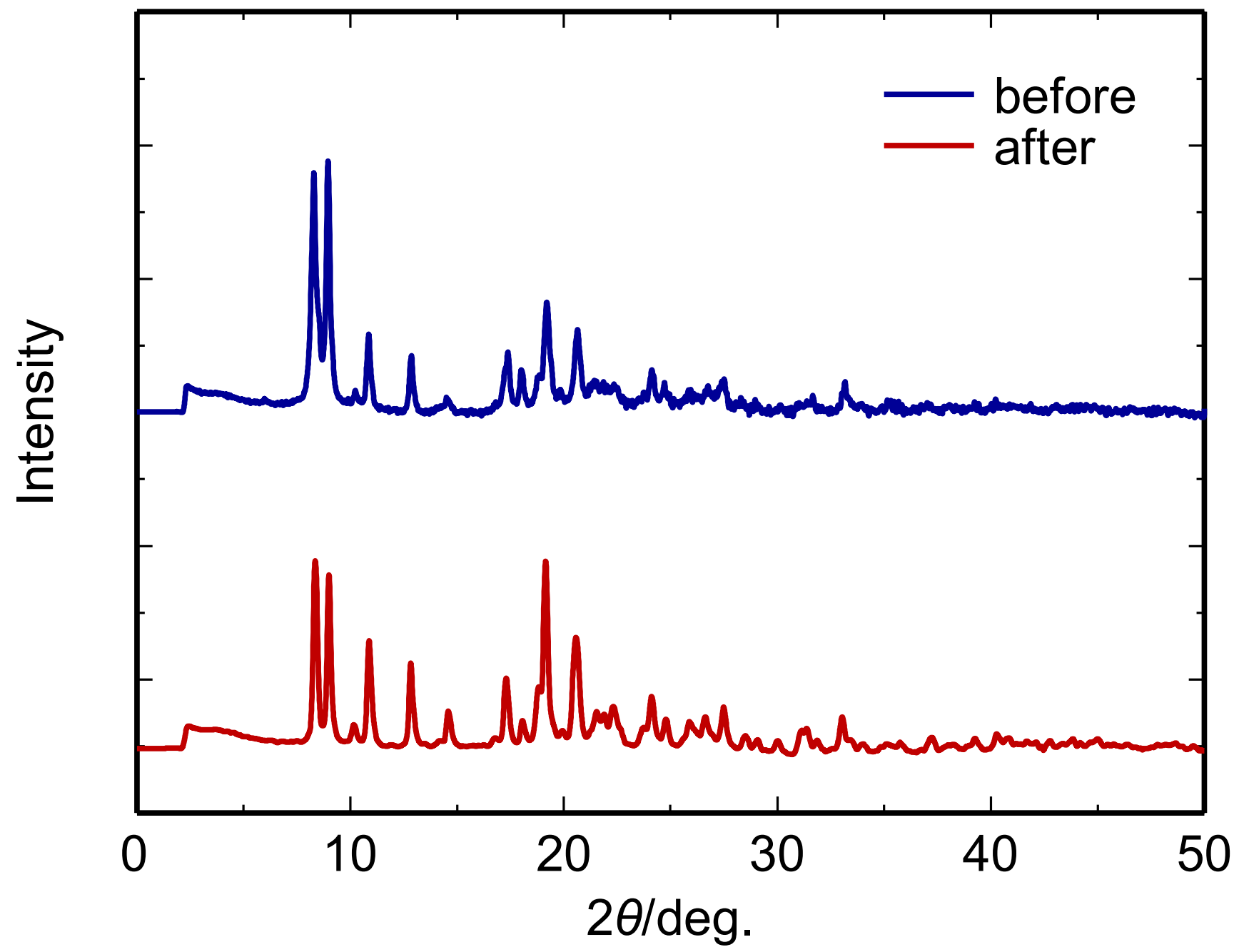

Fig. S9. PXRD patterns of **[Fe(H3P)]**$_{cryst}$ before (blue) and after (red) gas adsorption measurement.

6. Photocatalysis of the filtrate

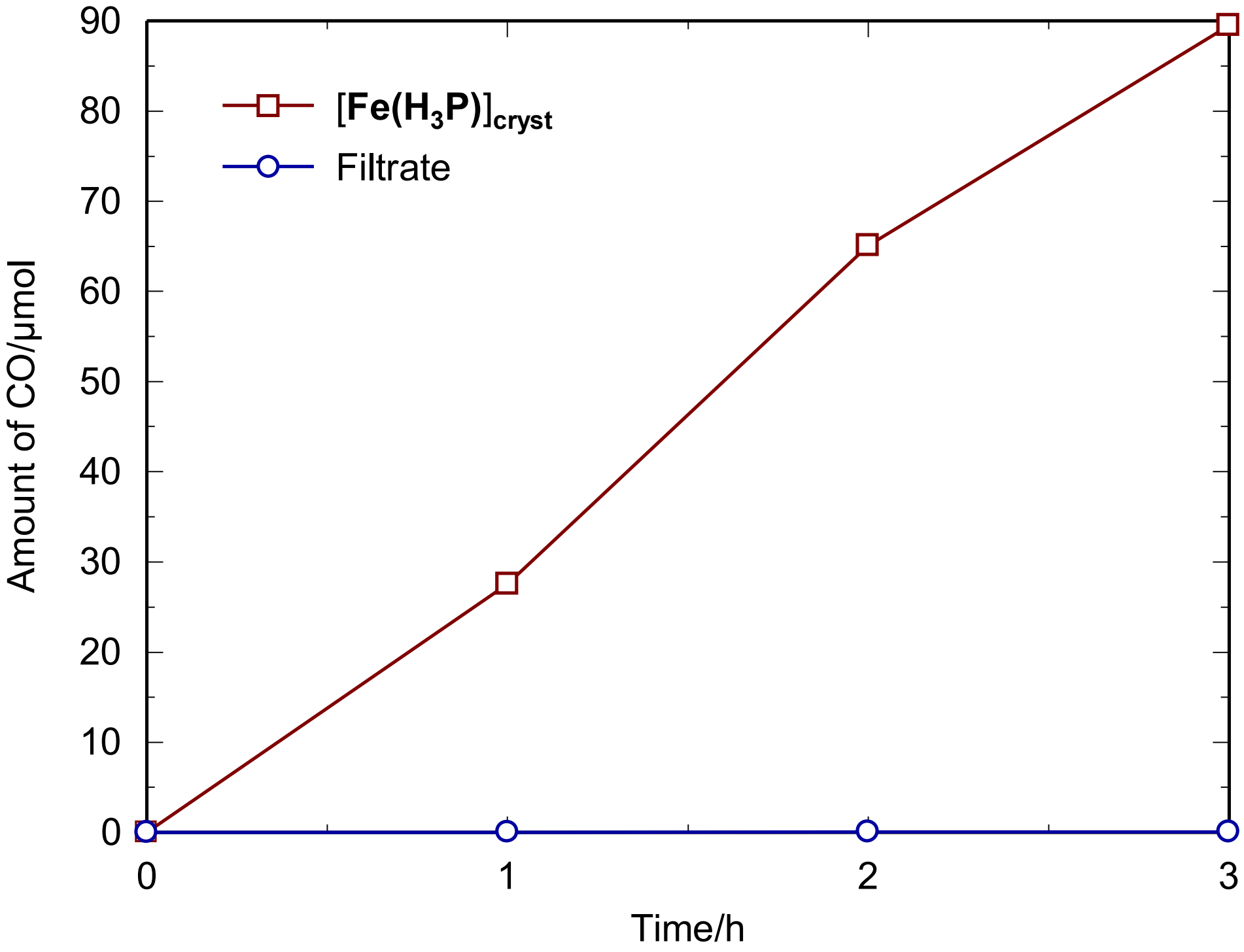

Fig. S10. Time course of CO production from **$[Fe(H_3P)]_{cryst}$** (red) and its filtrate after photocatalysis (blue).

Table S4. Details of the time course of CO production from **$[Fe(H_3P)]_{cryst}$** and its filtrate after photocatalysis.

Time/h	Amount of CO/μmol	
	**$[Fe(H_3P)]_{cryst}$**	Filtrate
1	27.53	0.02
2	65.05	0.05
3	89.47	0.07

7 Performance of porous crystalline solids

Catalytic activity, reaction condition and selectivity for photochemical CO_2 to CO conversion of porous crystalline solids are summarized in Table S5. TON values were calculated based on the number of active sites.

Table S5. Porous crystalline solids for photochemical CO_2 reduction to CO.

Catalyst	Solvent	Electron donor	Photosensitizer	Light/nm	Selectivity[a]/%	Production rate[b] /μmol g^{-1} h^{-1}	Ref.
**$[Fe(H_3P)]_{cryst}$**	**MeCN/TFE**	**BIH**	**$[Ir(ppy)_3]$**	**$400 \le \lambda \le 750$**	**> 99**	**1,800,000 (3 h) TON 4700**	**This work**
ZIF-67	MeCN/H_2O	TEOA	$[Ru(bpy)_3]Cl_2$	$\lambda \ge 420$	74	748,000 (0.5 h) TON 112 (0.5 h)	20
Co-MOL@GO	MeCN/H_2O	TEOA	$[Ru(phen)_3]Cl_2$	450	94	346,700 (10 h) TON 1065 (10 h)	35
Ru@Cu-HHTP	MeCN/H_2O	TEOA	/	$\lambda \ge 400$	92.9	130,000 (4 h) TON 173 (4 h)	36
**$[Fe(H_4P)]_{cryst}$**	**MeCN/TFE**	**BIH**	**$[Ir(ppy)_3]$**	**$400 \le \lambda \le 750$**	**> 99**	**110,000 (3 h) TON 270 (3 h)**	**This work**
**$[FeP]_{cryst}$**	**MeCN/TFE**	**BIH**	**$[Ir(ppy)_3]$**	**$400 \le \lambda \le 750$**	**> 99**	**58,000 (3 h) TON 70 (3 h)**	**This work**
$Ni_3(HITP)_2$	MeCN/H_2O	TEOA	$[Ru(bpy)_3]Cl_2$	420	97	34,500 (3 h) TON 28 (3 h)	23
Ni-MOL-100	MeCN/H_2O	TEOA	$[Ru(phen)_3](PF_6)_2$	$\lambda \ge 420$	100	31,610 (18 h) TON 100 (18 h)	37
FC1	MeCN/TFE	BIH	/	$400 \le \lambda \le 750$	> 99	29,100 (24 h) TON 1219 (24 h)	88
Ni(TPA/TEG)	MeCN/H_2O	TIPA	$[Ru(bpy)_3]Cl_2$	$\lambda \ge 400$	100	26,620 (2 h) TON 12 (2 h)	21
PCN-250-Fe_2Mn	MeCN/H_2O	TIPA	$[Ru(bpy)_3]Cl_2$	$420 \le \lambda \le 800$	82.17	21,510 (4 h) TON 29 (4 h)	32
UiO-Ru-2	MeCN/H_2O	BIH	$[Ru(bpy)_3]Cl_2$	$\lambda \ge 400$	100	21,375 (8 h)	38
PCN-250-Fe_2Zn	MeCN/H_2O	TIPA	$[Ru(bpy)_3]Cl_2$	$420 \le \lambda \le 800$	82.3	19,450 (4 h)	32
Co-2,3-DHTA-COF	MeCN/H_2O	TEOA	$[Ru(bpy)_3]Cl_2$	$\lambda \ge 420$	95.7	18,000 (4 h)	51
EPCo-COF-AT	MeCN/H_2O	TEOA	$[Ru(bpy)_3]Cl_2$	$\lambda \ge 420$	97.8	17,700 (2 h)	53
PCN-250-Fe_2Ni	MeCN/H_2O	TIPA	$[Ru(bpy)_3]Cl_2$	$420 \le \lambda \le 800$	81.84	15,860 (4 h)	32
PCN-250-Fe_2Co	MeCN/H_2O	TIPA	$[Ru(bpy)_3]Cl_2$	$420 \le \lambda \le 800$	83.53	14,010 (4 h)	32
PCN-250-Fe_3	MeCN/H_2O	TIPA	$[Ru(bpy)_3]Cl_2$	$420 \le \lambda \le 800$	75.45	13,450 (4 h)	32
Cu-HITP	MeCN/H_2O	TEOA	$[Ru(bpy)_3]Cl_2$	$\lambda \ge 420$	31.2	12,800 (4 h)	36
Ni MOLs	MeCN/H_2O	TEOA	$[Ru(bpy)_3]Cl_2$	$400 \le \lambda \le 800$	97.8	12,500 (2 h)	24
COF-367-Co-NSs	$H_2O/KHCO_3$	AA[c]	$[Ru(bpy)_3]Cl_2$	$\lambda \ge 420$	78	10,162 (2 h)	46

Table S5. Continued.

Catalyst	Solvent	Electron donor	Photosensitizer	Light/nm	Selectivity[a]/%	Production rate[b] /μmol g^{-1} h^{-1}	Ref.
Cu-THQ	MeCN/H_2O	TEOA	$[Ru(bpy)_3]Cl_2$	$\lambda \geq 420$	48.9	9,900 (4 h)	23
Fe/Ni-T120	MeCN/H_2O	TEOA	$[Ru(bpy)_3]Cl_2$	$\lambda \geq 420$	92.1	9,740(15 h)	43
Ni-MOF-W	MeCN/H_2O	TEOA	$[Ru(bpy)_3]Cl_2$	$\lambda \geq 400$	96.7	9,680 (1 h)	39
MCF-55-Ni	MeCN/H_2O	TEOA	$[Ru(phen)_3]Cl_2$	450	96.1	9,377 (46 h)	26
Zr_3CP_3–Ni	MeCN/H_2O	TEOA	$[Ru(bpy)_3]Cl_2$	$\lambda \geq 420$	96.7	9,300 (1 h)	40
Fe-MIL-101-NH_2	MeCN/H_2O	TEOA	$[Ru(bpy)_3]Cl_2$	$\lambda \geq 420$	69	9,400 (0.5 h)	20
Co-TAPT-COF-1	MeCN/H_2O	TEOA	$[Ru(bpy)_3]Cl_2$	$\lambda \geq 420$	42.6	8,390 (4 h)	52
Zn/Co/Mo-MOF	MeCN	TEOA	$[Ru(bpy)_3]Cl_2$	$\lambda \geq 420$	91.4	7,680 (6 h)	41
BIF-101	MeCN/H_2O	TEOA	$[Ru(bpy)_3]Cl_2$	$\lambda \geq 420$	84.1	5,830 (10 h)	30
Zr_3CP_3–Mn	MeCN/H_2O	TEOA	$[Ru(bpy)_3]Cl_2$	$\lambda \geq 420$	90.45	5,210 (1 h)	40
$(Co/Ru)_{2.4}$-UiO-67(bpydc)	MeCN/H_2O	TEOA	/	450	33.1	4,520 (16 h)	27
Co_6–MOF	MeCN/H_2O	TEOA	$[Ru(bpy)_3]Cl_2$	$\lambda \geq 420$	58.3	4,373 (3 h)	22
2D-Co_2TCPE-*PE*	MeCN/H_2O	TEOA	$[Ru(bpy)_3]Cl_2$	$420 \leq \lambda \leq 800$	74.3	4,147 (10 h)	33
Zn-ZIF-8	MeCN/H_2O	TEOA	$[Ru(bpy)_3]Cl_2$	$\lambda \geq 420$	47	3,600 (0.5 h)	20
Co_3O_4 HPs	MeCN/H_2O	TEOA	$[Ru(bpy)_3]Cl_2$	$\lambda \geq 420$	77.1	3,523 (1 h)	19
BIF-29	MeCN/H_2O	TEOA	$[Ru(bpy)_3]Cl_2$	$\lambda \geq 420$	82.6	3,334 (5 h)	28
Zr-DMBD−Co	MeCN/H_2O	TEOA	$[Ru(bpy)_3]Cl_2$	450	98	3,330 (10 h)	29
Co-UiO-67	MeCN/H_2O	TEOA	$[Ru(bpy)_3]Cl_2$	$400 \leq \lambda \leq 800$	31.7	3,292.5 (4 h)	34
2D-Ni_2TCPE-*PE*	MeCN/H_2O	TEOA	$[Ru(bpy)_3]Cl_2$	$420 \leq \lambda \leq 800$	97.3	3,000 (10 h)	33
2D-Ni_2TCPE-*ME*	MeCN/H_2O	TEOA	$[Ru(bpy)_3]Cl_2$	$420 \leq \lambda \leq 800$	96.9	2,944 (10 h)	33
2D-Co_2TCPE	MeCN/H_2O	TEOA	$[Ru(bpy)_3]Cl_2$	$420 \leq \lambda \leq 800$	75.8	2,560 (10 h)	33
Zr-NDI@Ru-tpy	DMF/H_2O	TEA	/	$\lambda \geq 420$	97	2449 (6 h)	44
Cu HKUST-1	MeCN/H_2O	TEOA	$[Ru(bpy)_3]Cl_2$	$\lambda \geq 420$	44	2,400 (0.5 h)	20
2D-Ni_2TCPE	MeCN/H_2O	TEOA	$[Ru(bpy)_3]Cl_2$	$420 \leq \lambda \leq 800$	97.2	2,000 (10 h)	33
Zr-UIO-66-NH_2	MeCN/H_2O	TEOA	$[Ru(bpy)_3]Cl_2$	$\lambda \geq 420$	43	1,800 (0.5 h)	20
MCOF-Co-315	MeCN	TEOA	$[Ru(bpy)_3]Cl_2$	$390 \leq \lambda \leq 740$	/	1616 (8 h)	54

Table S5. Continued.

Catalyst	Solvent	Electron donor	Photosensitizer	Light/nm	Selectivity[a]/%	Production rate[b] /μmol g^{-1} h^{-1}	Ref.
TFBD-COF-Co-SA	MeCN	TEOA	$[Ru(bpy)_3]Cl_2$	$\lambda \geq 420$	90	1,480 (5 h)	50
Re-Bpy-sp^{2}c-COF	MeCN	TEOA	$Ir[dF(CF_3)ppy]_2(dtbpy)PF_6$	$\lambda \geq 420$	86	1,400 (5 h)	48
MOF-Co	$MeCN/H_2O$	TIPA	$[Ru(bpy)_3]Cl_2$	$420 \leq \lambda \leq 800$	47.4	1,140.0 (12 h)	31
PMOF/Re	DMF/H_2O	BIH	/	$\lambda \geq 500$	99	1,070 (59 h)	42
Re-Bpy-sp^{2}c-COF	MeCN	TEOA	/	$\lambda \geq 420$	81	1,040 (17.5 h)	48
DQTP COF-Co	MeCN	TEOA	$[Ru(bpy)_3]Cl_2$	$\lambda \geq 420$	59	1,020 (4 h)	47
Ni-TpBpy	$MeCN/H_2O$	TEOA	$[Ru(bpy)_3]Cl_2$	$\lambda \geq 420$	96	966 (6 h)	25
Re-COF	MeCN	TEOA	/	$\lambda \geq 420$	98	750 (20 h)	45
Ni-PCD@TD-COF	$MeCN/H_2O$	TEOA	$[Ru(bpy)_3]Cl_2$	$\lambda \geq 420$	98	480 (2 h)	49
Zr-MBA-Ru/ Re-MOF	H_2O	/	/	$400 \leq \lambda \leq 800$	99	440 (1 h)	108
MOF-Ni	$MeCN/H_2O$	TIPA	$[Ru(bpy)_3]Cl_2$	$420 \leq \lambda \leq 800$	97.7	371.6 (12 h)	31
BUT-33(Pd)	H_2O	TEA	/	Xe lamp	48.3	269 (6 h)	109
MOF-Cu	$MeCN/H_2O$	TIPA	$[Ru(bpy)_3]Cl_2$	$420 \leq \lambda \leq 800$	77.3	232.0 (12 h)	31
NH_2-UiO-66-tpy	MeCN	TEOA	/	$350 \leq \lambda \leq 780$	/	209.2 (8 h)	110
MOF-525-Co	MeCN	TEOA	/	$400 \leq \lambda \leq 800$	85.2	200.6 (6 h)	106
In-$Fe_{1.91}$TCPP-MOF	EtOAc	L-AP	/	$\lambda \geq 400$	99.5	144 (24 h)	60
BUT-110-65%-Co	H_2O	TEA	/	$320 \leq \lambda \leq 780$	88.7	70.8 (8 h)	111

[a] Selectivity calculated by the molar ratio of the main product versus all the products. [b] Production rate calculated by amount of CO production versus reaction time described in the parentheses. [c] AA = ascorbic acid.

8. Quantum yield

8.1 Determination of the light intensity

The light intensity was determined by the standard ferrioxalate actinometry[112,113] to estimate the apparent quantum yield (AQY) and external quantum efficiency (EQE) for CO production. 2 mL (V_1) of 10 mM $K_3[Fe^{III}(C_2O_4)_3]$ in 0.05 M H_2SO_4 was irradiated with Xe lamp combined with band pass filter (400 nm) under efficient stirring. After the irradiation, 0.5 mL (V_2) of the irradiated solution was added into a mixed solution containing 1,10-phenanthroline and top up to 10 mL (V_3) by H_2O and incubated in the dark for 1 h. The components of the mixed solution are 2 mL of 0.1% phenanthroline aqueous solution and 0.25 mL of buffer solution (1 M sodium acetate: 0.05 M H_2SO_4: H_2O = 60: 36: 4, v/v/v). After 1 h, the absorbance of the mixed solution was measured. Absorbance at 510 nm indicates the formation of $[Fe^{II}(phen)_3]^{2+}$ complex due to the amount of photon absorbed.

The rate of photon flux of the light I (einstein s^{-1}) was evaluated by

$$I = \frac{\Delta A V_1 V_3}{\Phi_\lambda \varepsilon_{510\ \mathrm{nm}} V_2 L t}, \tag{S2}$$

where ΔA is the absorbance at 510 nm, V_1 = 0.002 L, V_2 = 0.0005 L, V_3 = 0.01 L, Φ_λ is the quantum yield for $[Fe^{II}(phen)_3]^{2+}$ at λ nm. Here, Φ_λ = 1.14 at 400 nm, 1.11 at 420 and 450 nm, and 0.93 at 470 nm.[112] ε_{510nm} is the molar absorption coefficient of $[Fe^{II}(phen)_3]^{2+}$ at 510 nm (11100 mol^{-1} L cm^{-1}), L is the length of cuvette (1 cm) and t is the irradiation time (0 s, 20 s, 40 s and 60 s). Absorbance changes of $K_3[Fe^{III}(C_2O_4)_3]$ and 1,10-phenanthroline mixture solution and time course changes of the absorbance at 510 nm were shown in Fig. S11a. $\Delta A/t$ in Eq.(S2) were taken from the slope shown in Fig. S11b. AQY[92] and EQE[93] were evaluated by

$$\mathrm{AQY} = \frac{R_{\mathrm{CO}}}{I} \times 100, \tag{S3}$$

and

$$\mathrm{EQE} = \frac{2R_{\mathrm{CO}}}{I} \times 100, \tag{S4}$$

where R_{CO} (mol s^{-1}) is the rate of CO production. The factor of 2 in the numerator of the EQE equation reflects that the conversion of CO_2 to CO is a two-electron reduction process.

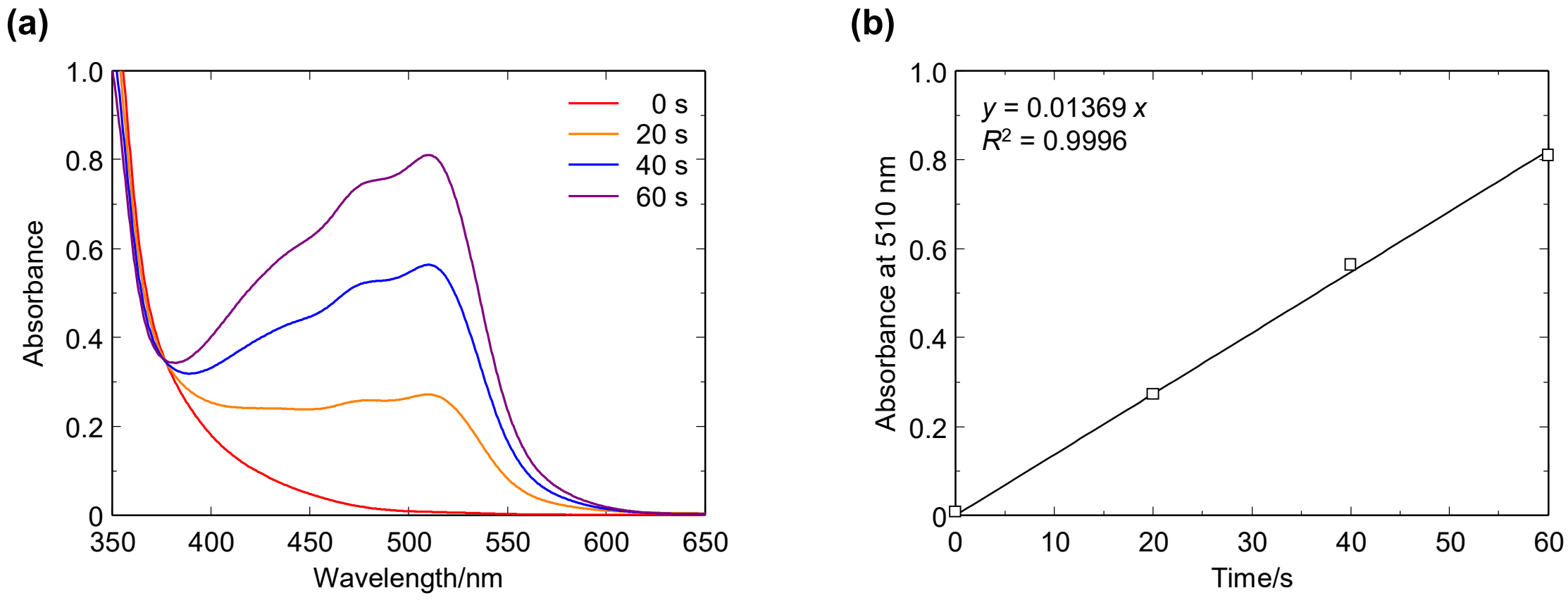

Fig. S11. (a) Absorbance changes of $K_3[Fe^{III}(C_2O_4)_3]$ and 1,10-phenanthroline mixture solution after photo irradiation at 400 nm and (b) time course changes of the absorbance at 510 nm measured from the solution after photo irradiation at 400 nm.

8.2 Comparison of the quantum yield

Table S6. Quantum yields of molecule-based heterogeneous catalysts for CO_2 reduction.[a]

Catalyst	Solvent	Electron dnonor	PS	Wavelength/nm	AQY/%	EQE/%	Ref.
**$[Fe(H_3P)]_{cryst}$**	MeCN/TFE	BIH	$Ir(ppy)_3$	400	2.97	5.95	**This work**
MCOF-Co-315	MeCN/H_2O	TEOA	$Ru(bpy)_3Cl_2$	450	/	9.13	54
Ru@Cu-HHTP	MeCN/H_2O	TEOA	/	450	/	2.87	36
PCN-250-Fe_2Mn	MeCN/H_2O	TIPA	$Ru(bpy)_3Cl_2$	450	/	2.60	32
ZIF-67	MeCN/H_2O	TEOA	$[Ru(bpy)_3]Cl$	/	1.55[b]	/	20
TNP-MOF	H_2O	/	/	450	/	2.03	114
Cu(I)-MOF	H_2O	/	/	450	1.32	/	115
$Ni_3(HITP)_2$	MeCN/H_2O	TEOA	$[Ru(bpy)_3]Cl$	420	/	0.92	23
FC1	MeCN/TFE	BIH	/	400	0.298	/	88
Zr-MBA-Ru/Re-MOF	H_2O	/	$Ru(bpy)_3Cl_2$	450	/	0.11	108

[a] In all system, the main product is CO. [b]The definition of quantum yield and the wavelength of irradiated light were not described in the literature.

9. Characteristics of the crystal structure of $[FeP]_{cryst}$ and $[Fe(H_4P)]_{cryst}$

9.1 Crystallographic data of FeP

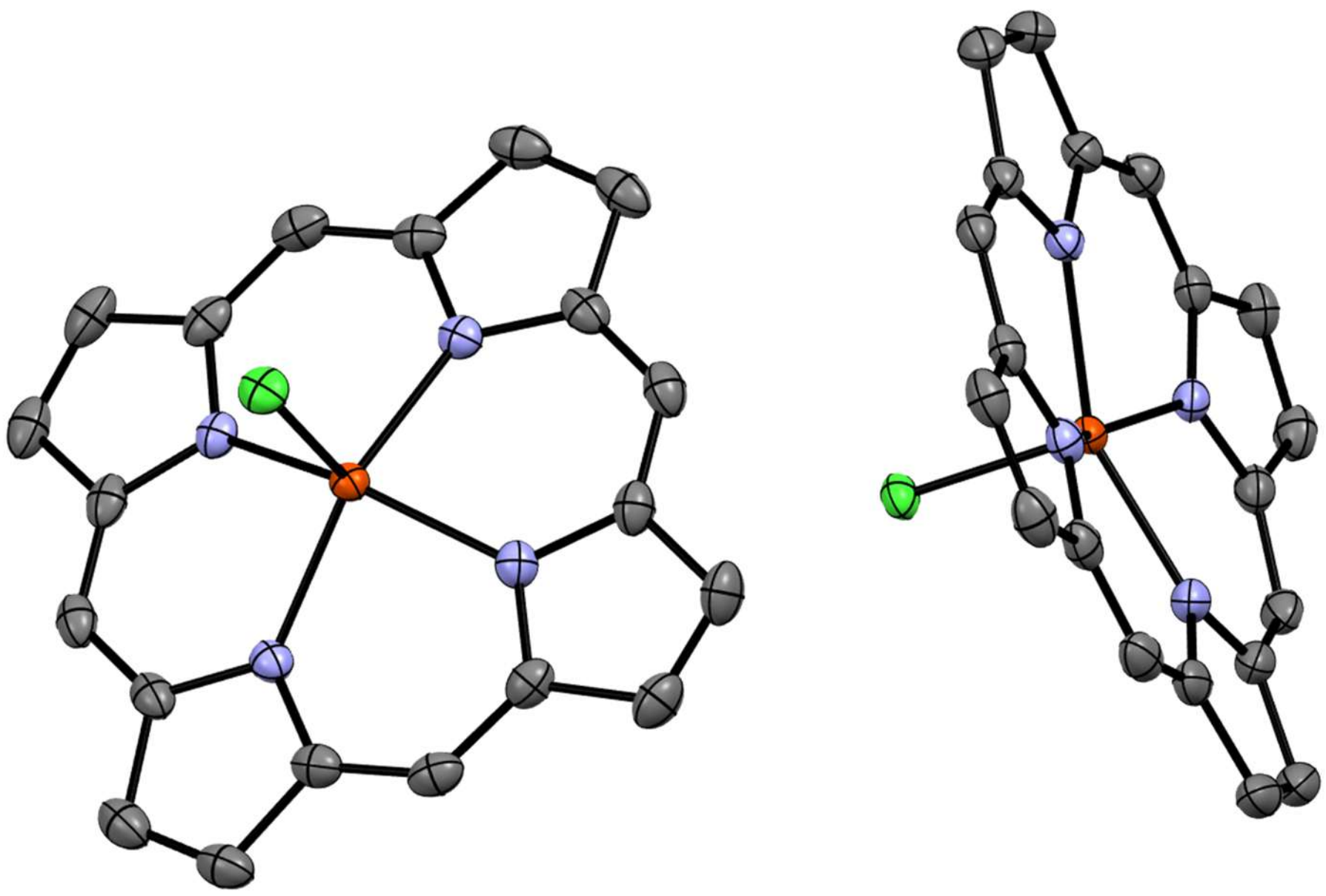

Fig. S12. ORTEP drawings of **FeP** (50% probability ellipsoids). Hydrogen atoms are omitted for clarity. C = gray, N = blue, O = red, Cl = green, and Fe = orange.

Table S7. Summary of crystallographic data for **FeP**.

	**FeP**
Formula	$(C_{20}H_{12}FeClN_4)_2$
Fw	1624.0
Crystal color, habit	dark orange, plate
Crystal size / mm^3	0.125 × 0.049× 0.037
Crystal system	monoclinic
Space group	$P2_1/c$
a / Å	17.2346(5)
b / Å	12.0382(2)
c / Å	17.7243(5)
α / °	90
β / °	115.378(4)
γ / °	90
V / Å^3	3322.46(18)
Z	8
F(000)	1624.0
d_{calc} / g cm^{-3}	1.598
μ(MoKa) / mm^{-1}	1.080
T / K	123(2)
R_1	0.0317
wR_2	0.0800
GooF	1.035

9.2 Packing structure of [FeP]$_{cryst}$

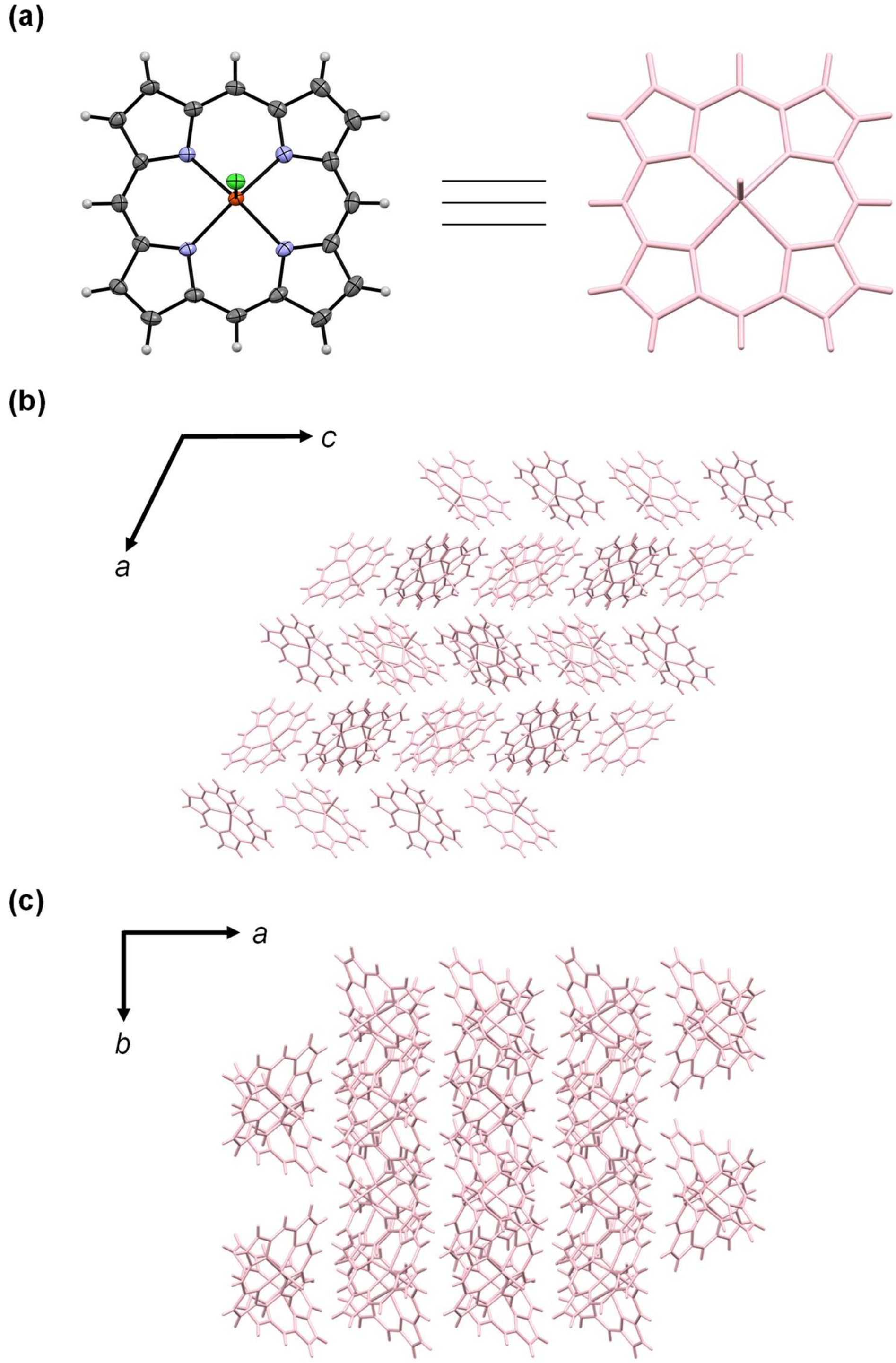

Fig. S13. (a) Correspondence between ORTEP drawing and the capped stick representation of **FeP**. In the capped stick representation, pink = porphyrin scaffold. (b) Packing structure of [**FeP**]$_{cryst}$ along the *b* axis. (c) Packing structure of [**FeP**]$_{cryst}$ along the *c* axis.

9.3 Crystallographic data of $[Fe(H_4P)]_{cryst}$

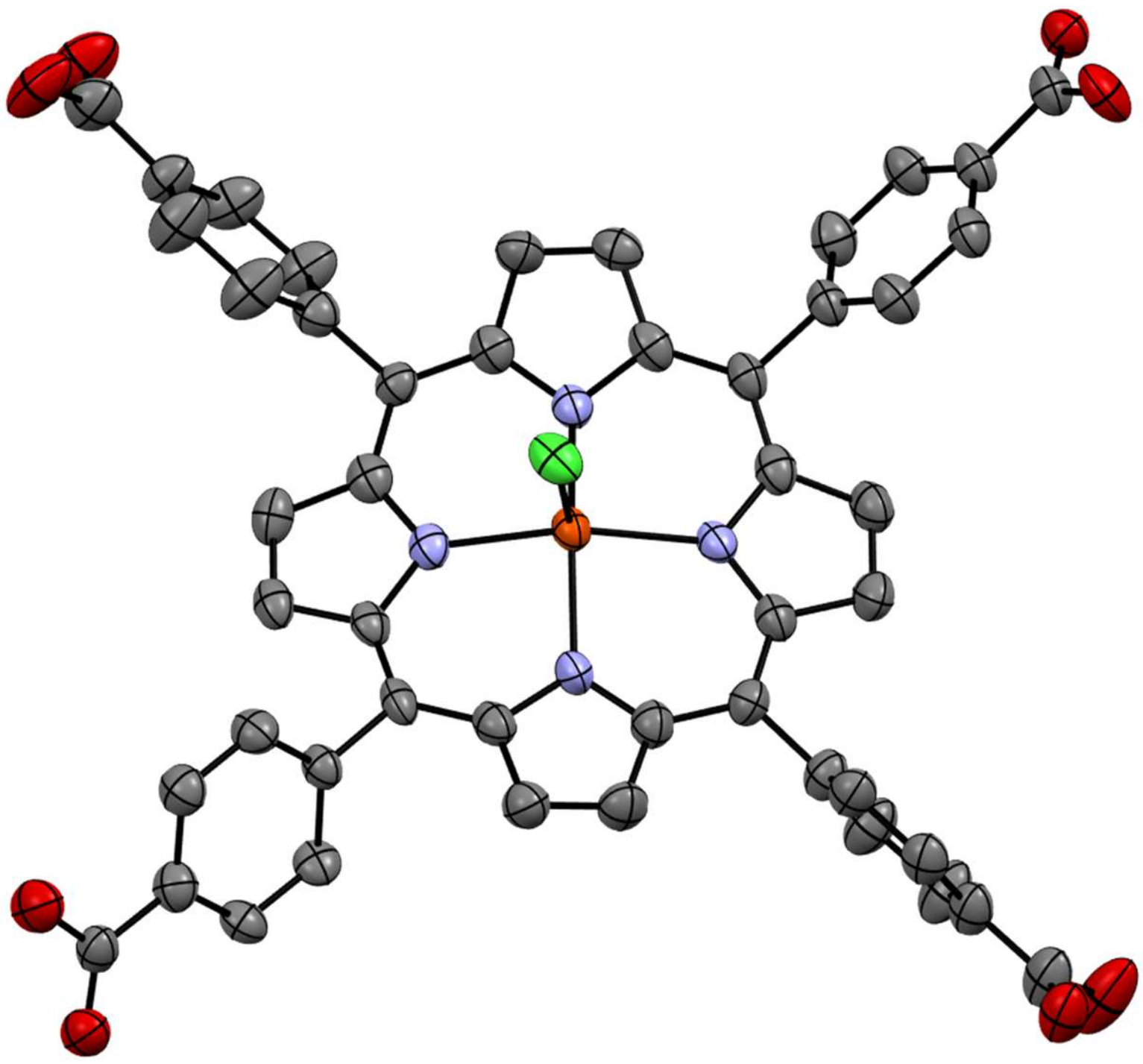

Fig. S14. ORTEP drawing of **Fe(H$_4$P)** (50% probability ellipsoids). Hydrogen atoms are omitted for clarity. C = gray, N = blue, O = red, Cl = green, and Fe = orange.

Table S8. Summary of crystallographic data for **Fe(H$_4$P)**.

	**Fe(H$_4$P)**
Formula	$C_{48}H_{28}FeClN_4O_8$
Fw	880.04
Crystal color, habit	dark brown, plate
Crystal size / mm^3	0.20 × 0.13× 0.05
Crystal system	Triclinic
Space group	$P\bar{1}$
a / Å	9.4258(8)
b / Å	15.6984(13)
c / Å	15.9354(15)
α / °	70.250(8)
β / °	87.219(7)
γ / °	73.749(7)
V / Å^3	2127.8(3)
Z	2
F(000)	902.0
d_{calc} / g cm^{-3}	1.374
μ(MoKa) / mm^{-1}	0.477
T / K	123(2)
R_1	0.1105
wR_2	0.3461
GooF	1.074

9.4 Packing structure of [Fe(H_4P)]$_{cryst}$

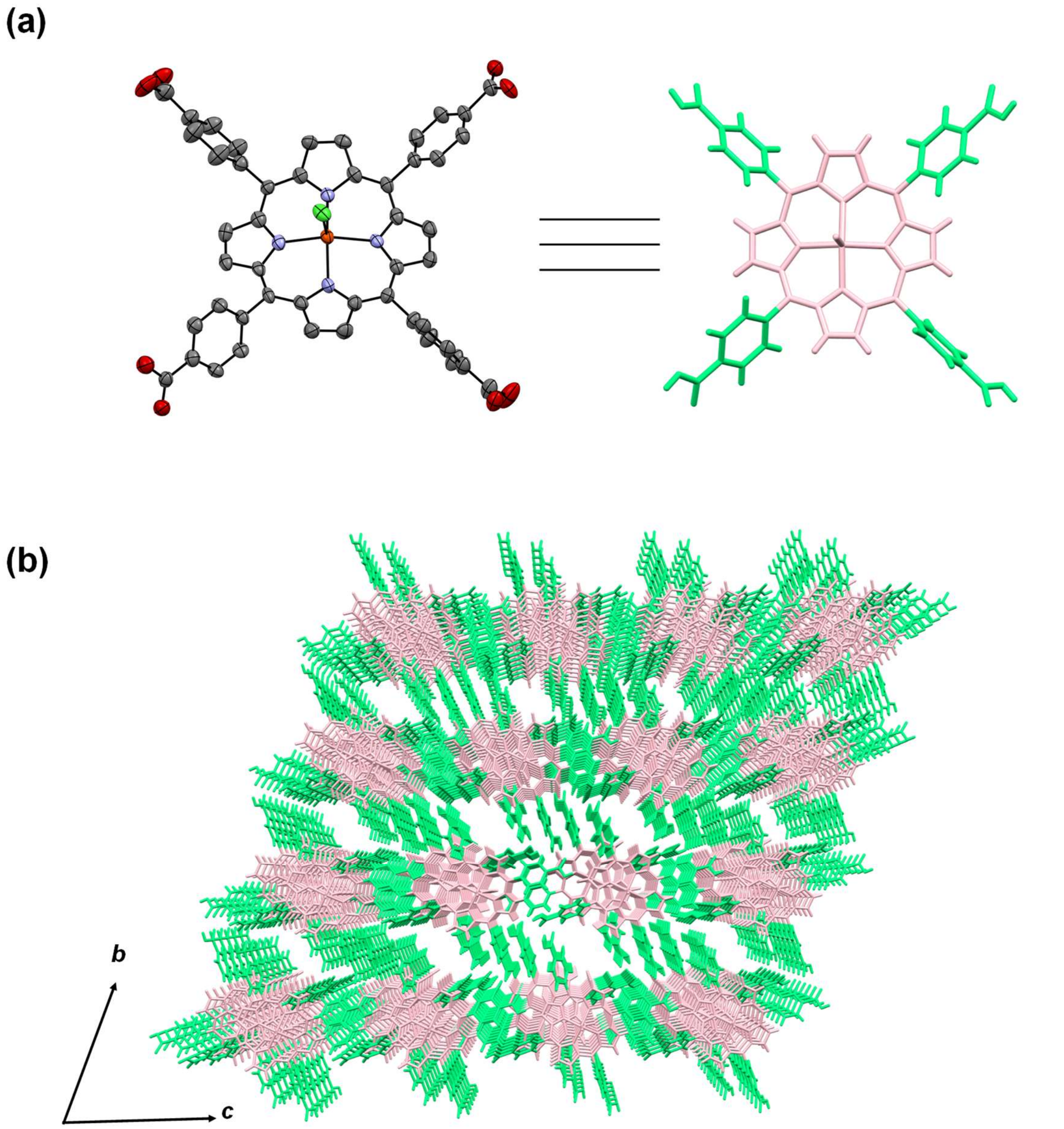

Fig. S15. (a) Correspondence between ORTEP drawing and the capped stick representation of **Fe(H_4P)**. In the capped stick representation, pink = porphyrin scaffold and green = carboxyl group. (b) Packing structure of [**Fe(H_4P)**]$_{cryst}$ along the *a* axis.

10. Photocatalysis of [Fe(H_3P)]$_{cryst}$, [Fe(H_4P)]$_{cryst}$ and [FeP]$_{cryst}$

Table S9. Time course of CO production catalyzed by **[Fe(H_3P)]$_{cryst}$**, **[Fe(H_4P)]$_{cryst}$** and [**FeP**]$_{cryst}$.

Time/h	Amount of CO/mmol g^{-1}		
	**[Fe(H_3P)]$_{cryst}$**	**[Fe(H_4P)]$_{cryst}$**	**[FeP]$_{cryst}$**
1	1700	190	150
2	4100	260	160
3	5600	320	180

11. Proton conductivity measurements

For the proton conductivity test, the crystals were pelletized by pressing in a cylindrical die (1 cm diameter, about 0.7 mm thickness) at ca. 0.3 GPa for 5 minutes. The resistance was estimated from the equivalent circuit fitting of the Nyquist plots. Proton conductivity was calculated with σ = (l/R) (t/a), where σ is conductivity, R is the resistance inside the catalysts, and t and a are the thickness and area of the pellet, respectively. The activation energy, E_a, was calculated from $\ln\sigma T = \ln\sigma_0 - E_a/k_B T$, where σ is the conductivity (S cm^{-1}), k_B is the Boltzmann constant (8.6 × 10^{-5} eV/K) and T is the temperature (K). AC impedance measurement was performed using a 1260 Impedance/Gain-Phase Analyzer (Solartron) with a temperature and humidity chamber IW222 (Yamato) from 1 MHz–1 Hz.

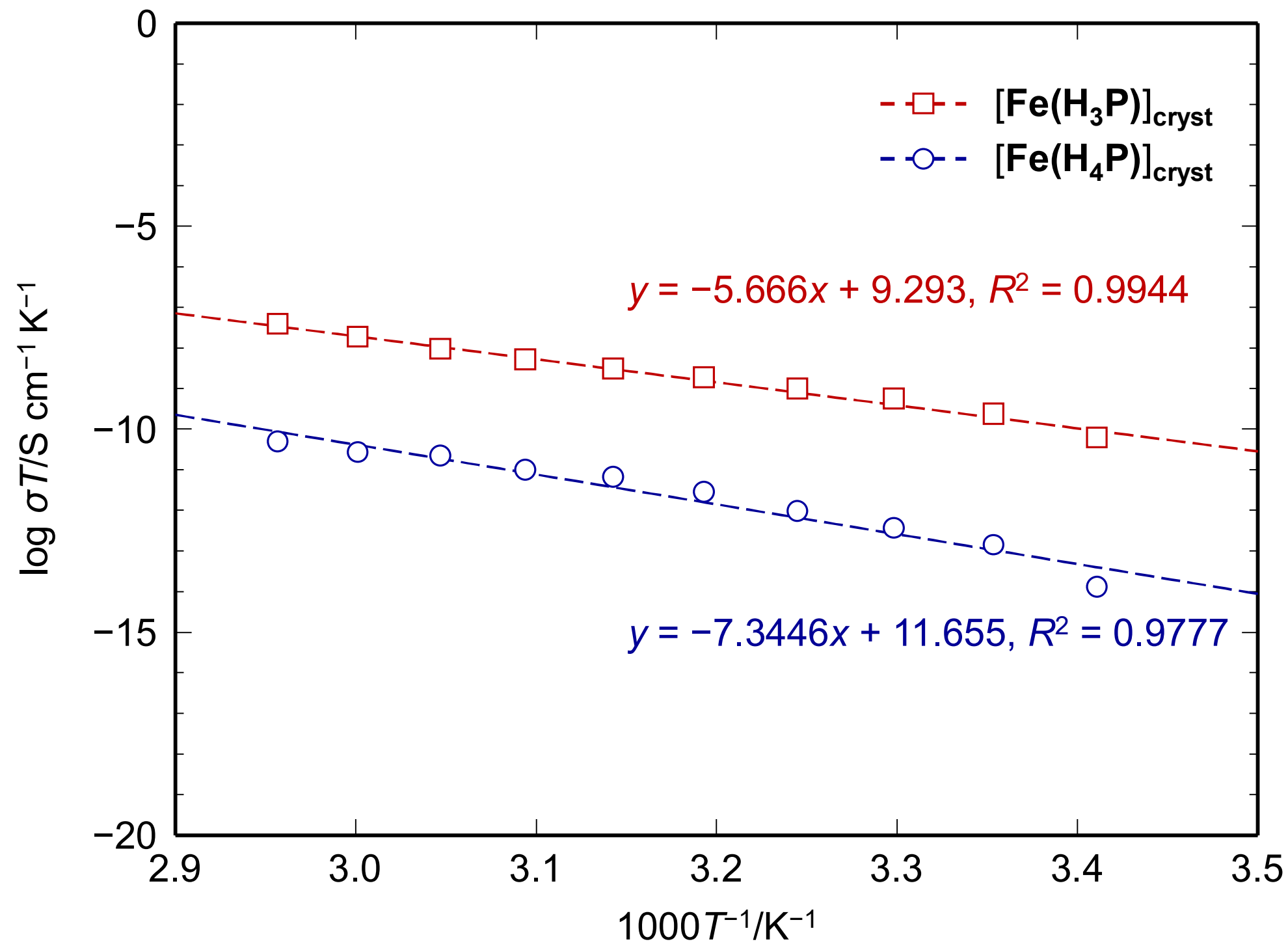

Fig. S16. Arrhenius plots of the proton conductivity of **[Fe(H$_3$P)]$_{cryst}$** (red) and **[Fe(H$_4$P)]$_{cryst}$** (blue) under 95% RH conditions. Least squares fits are shown as dashed lines.

12. Water adsorption measurements

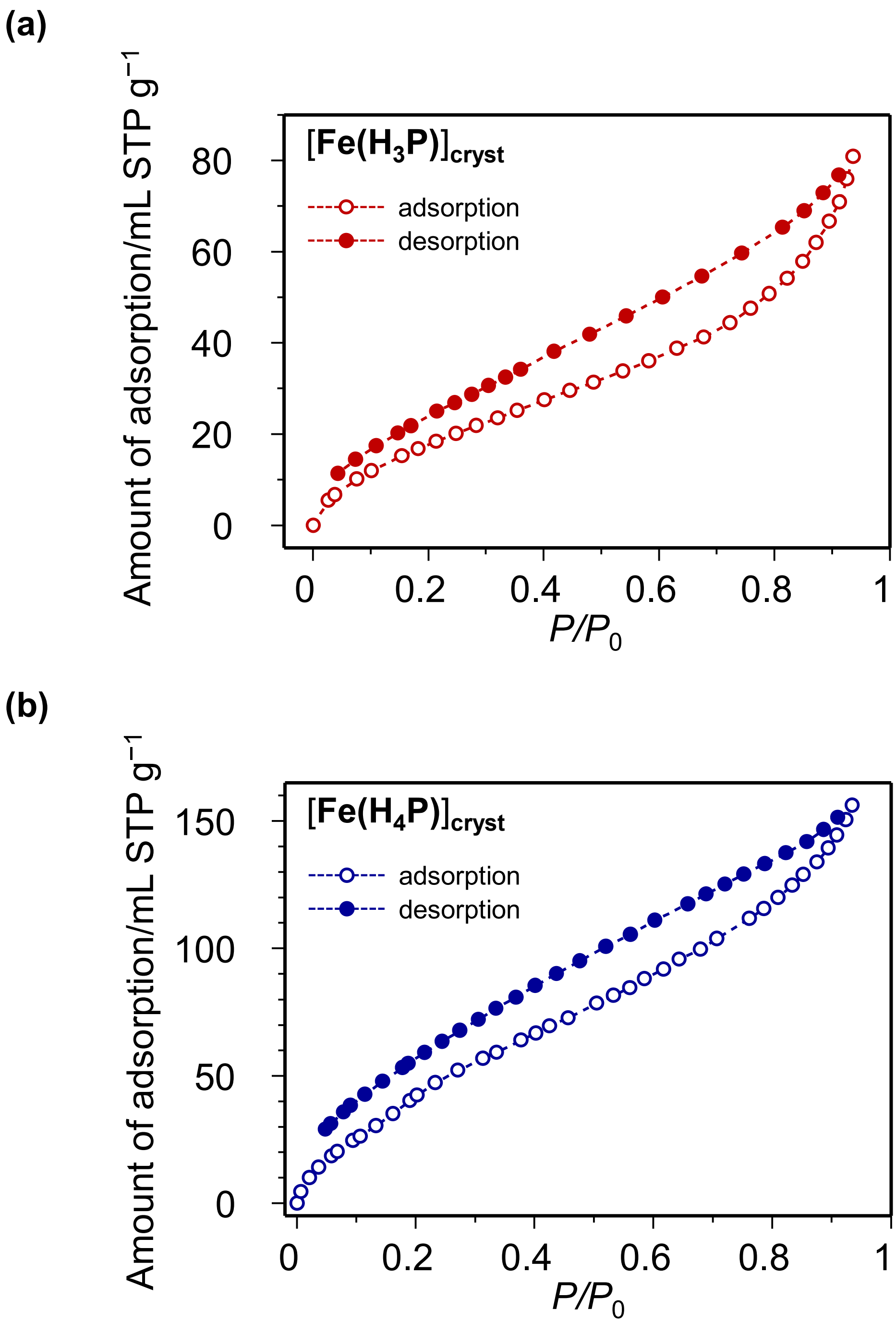

Fig. S17. Adsorption and desorption isotherms of water for (a) **[Fe(H$_3$P)]$_{cryst}$** and (b) **[Fe(H$_4$P)]$_{cryst}$** at 298 K.

13. Characteristics of the crystal structure of [Fe(H_3P)-H_2O]$_{cryst}$

13.1 Crystallographic data of Fe(H_3P)-H_2O

Table S10. Summary of crystallographic data for **Fe(H_3P)-H_2O**.

	**Fe(H_3P)-H_2O**
Formula	$C_{48}H_{27}FeN_4O_8$ • 3.1H_2O
Fw	899.43
Crystal color, habit	Dark brown, block
Crystal size / mm^3	0.20 × 0.13× 0.05
Crystal system	Monoclinic
Space group	$P\ 2_1/c$
a / Å	15.5314(6)
b / Å	7.9913(2)
c / Å	21.0109(6)
α / °	90
β / °	99.207(3)
γ / °	90
V / Å^3	2574.19(14)
Z	2
F(000)	928.0
d_{calc} / g cm^{-3}	1.160
μ(MoKa) / mm^{-1}	0.350
T / K	100(2)
R_1	0.0999
wR_2	0.2958
GooF	1.080

13.2 Packing structure of [Fe(H_3P)-H_2O]$_{cryst}$

Based on the valence state of the Fe atom, the molecule is expected to have three carboxylic acid groups and one deprotonated carboxylate group for charge neutrality. Therefore, one of the two carboxyl groups directly coordinated to the Fe atom must be in its deprotonated (carboxylate) form. However, the **Fe(H_3P)** moiety is located on a crystallographic inversion center, making the two coordinating carboxyl groups symmetry equivalent. To satisfy the overall charge balance, these two groups were modeled as a 50:50 disordered superposition of a carboxylic acid and a carboxylate group. This was implemented by assigning an occupancy of 0.5 to the disordered hydrogen atom, which was positioned via geometrical calculation to form a hydrogen bond with a crystalline water molecule.

The total occupancy of crystalline water molecules was fixed at 3.1, consistent with the water adsorption measurement (Fig. S18a). The positions of the crystalline water molecules were identified from residual electron density maps and were modeled to form hydrogen bonds with the coordinating carboxylic acid/carboxylate groups.

(a)

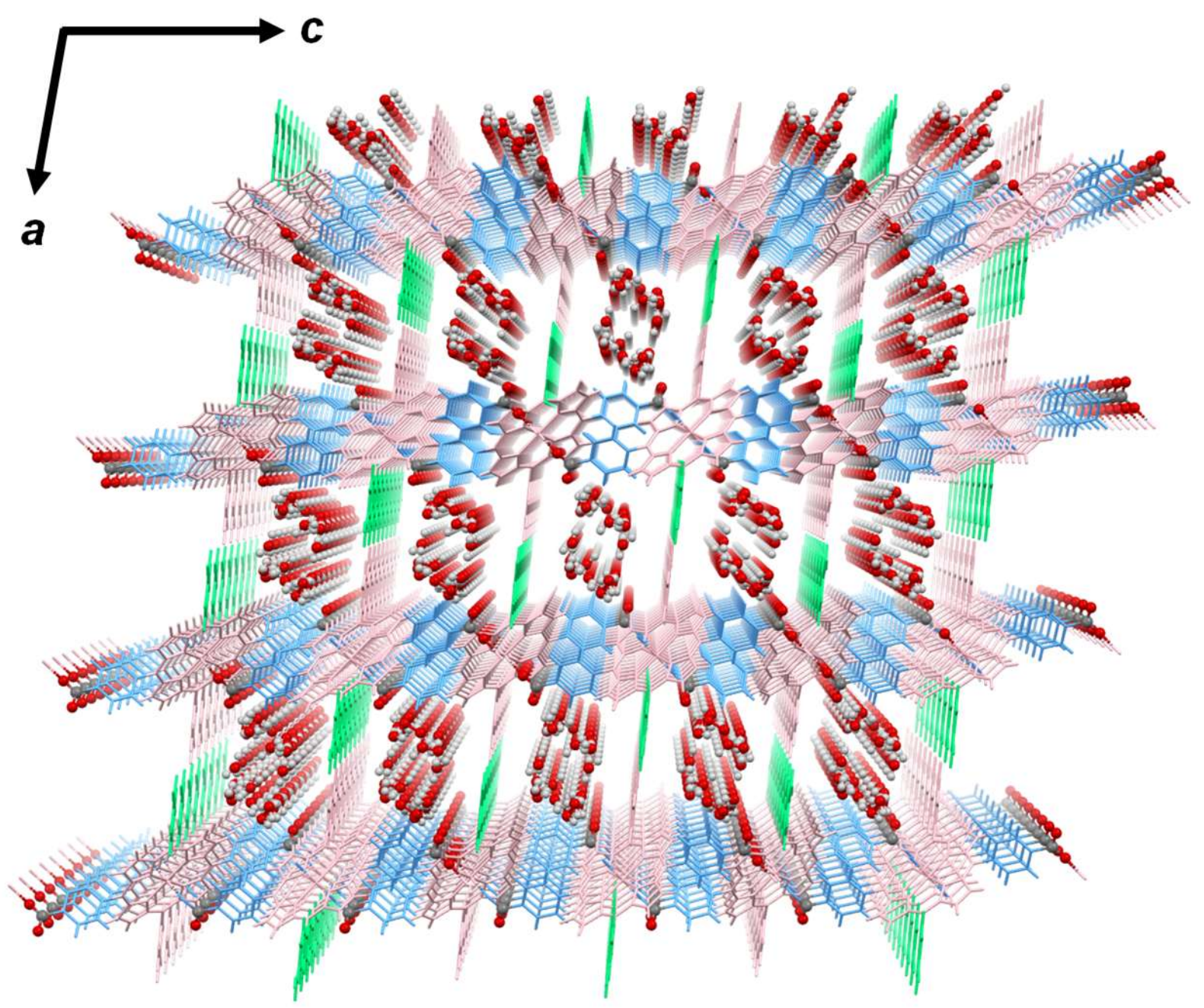

(b)

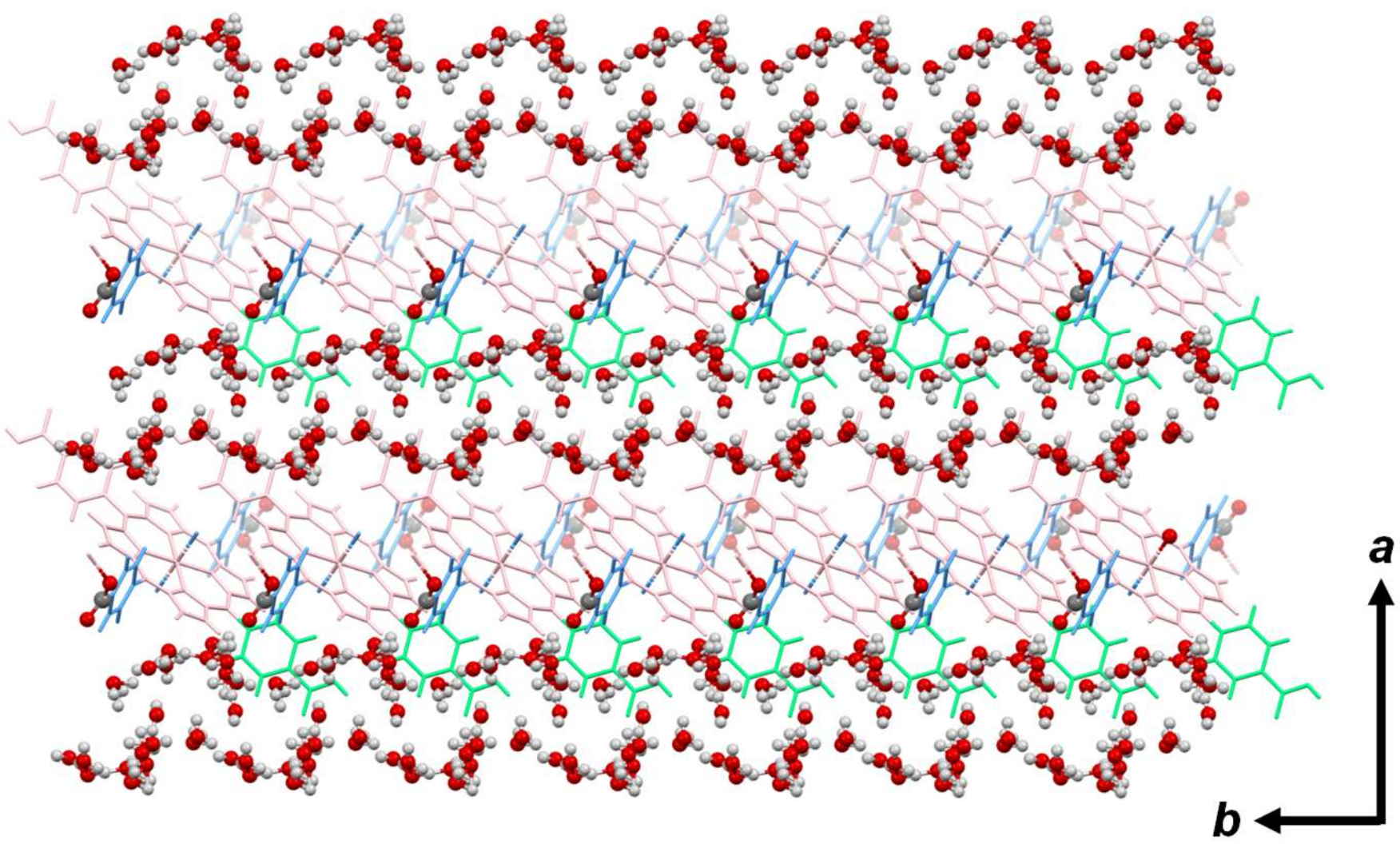

Fig. S18. (a) Packing structure of **$[Fe(H_3P)$-$H_2O]_{cryst}$** along *b* axis. For clarity, water molecules (H_2O) and carboxylate (COO) are represented as ball and stick. Porphyrin scaffold (pink), carboxyl groups (green) and phenyl moiety of carboxylate groups (blue) are represented as capped stick. (b) Hydrogen bonding network between carboxylate groups and water molecules along *c* axis.

14. Chromatograph data

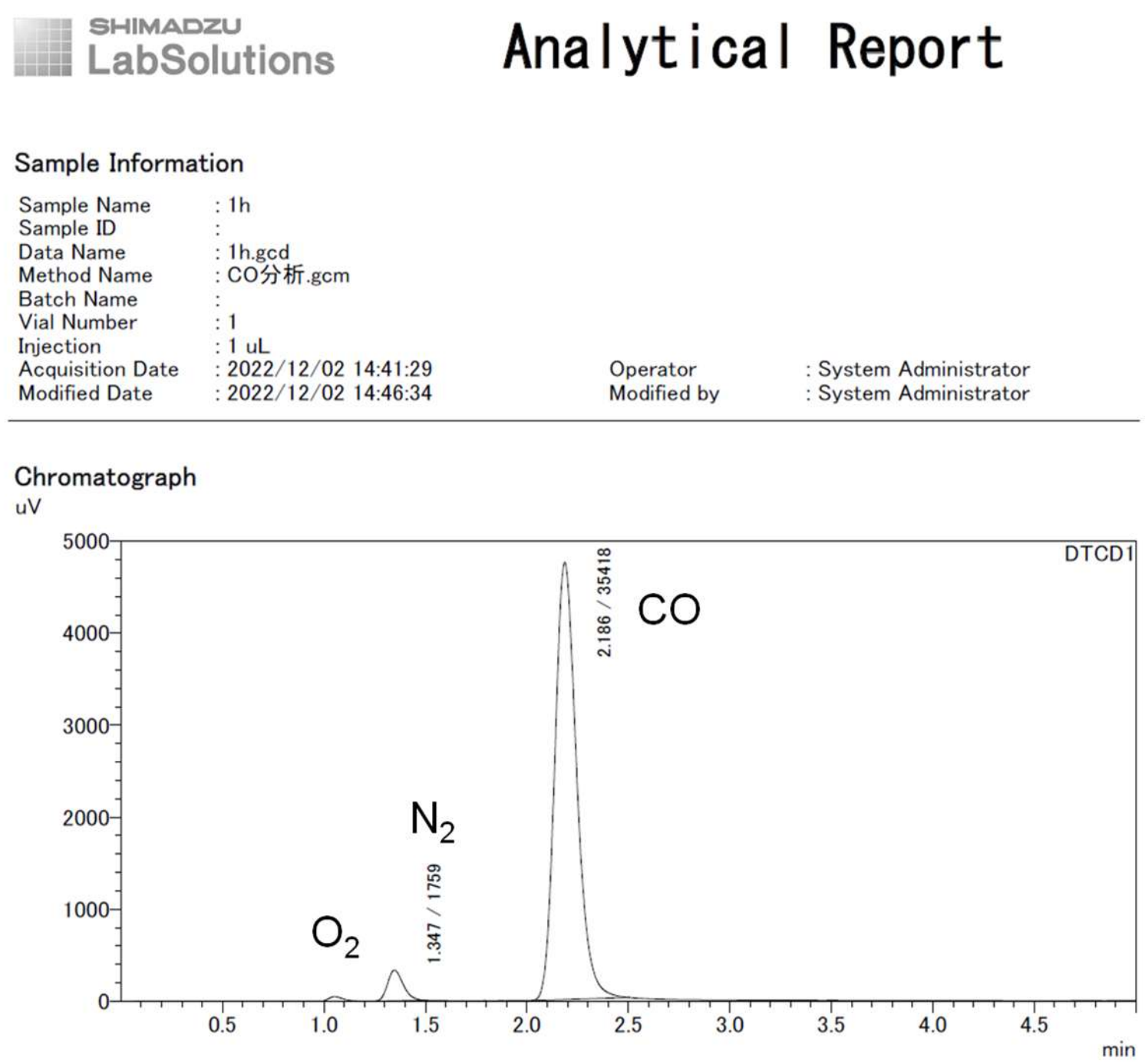

Chart S1. Chromatogram of CO in Fig.2b when **[Fe(H$_3$P)]$_{cryst}$** was used as a catalyst (1 h).

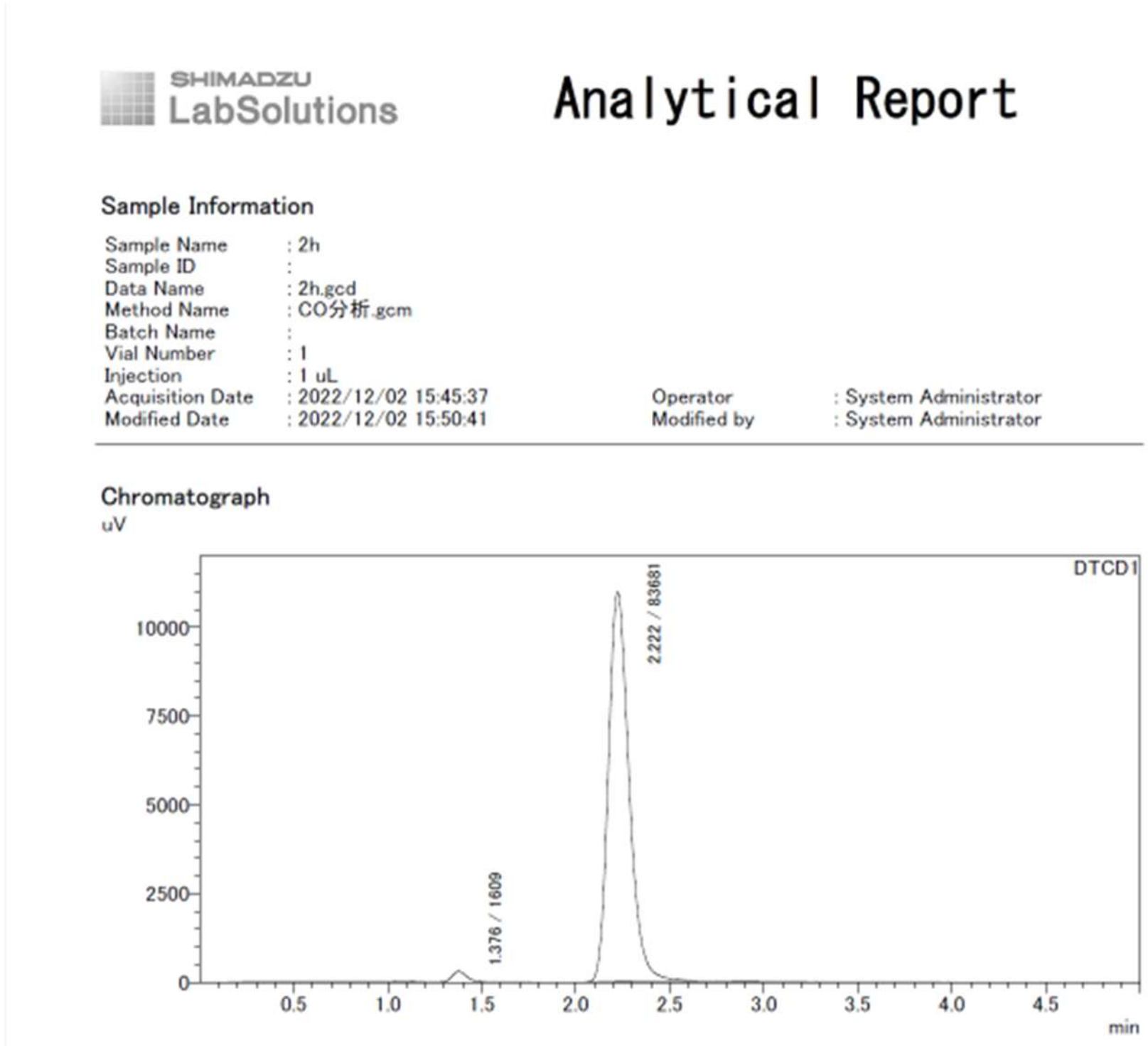

Chart S2. Chromatogram of CO in Fig.2b when **[Fe(H$_3$P)]$_{cryst}$** was used as a catalyst (2 h).

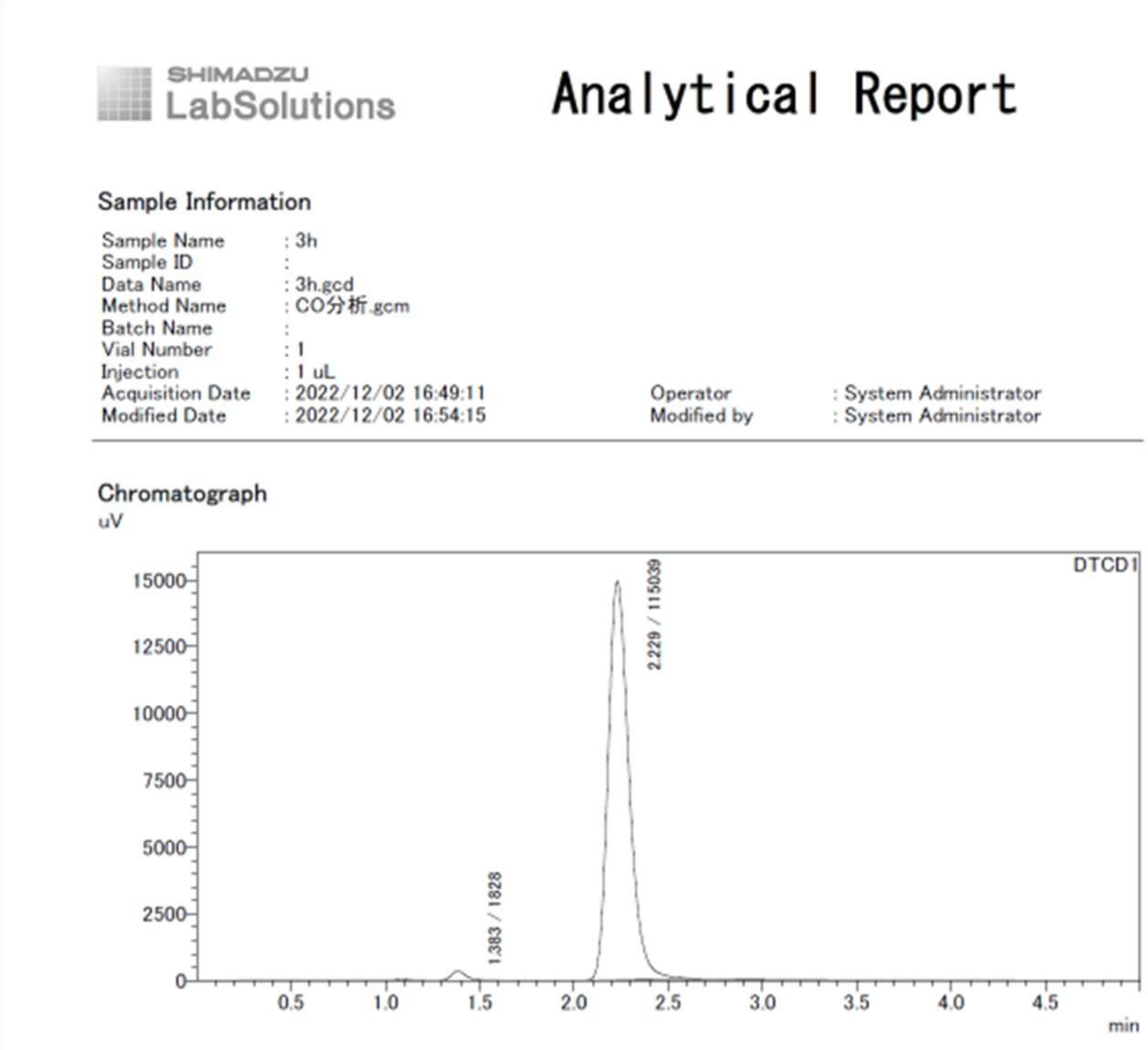

Chart S3. Chromatogram of CO shown in Fig. 2b when **[Fe(H$_3$P)]$_{cryst}$** was used as a catalyst (3 h) and in Table 1 (Entry 1).

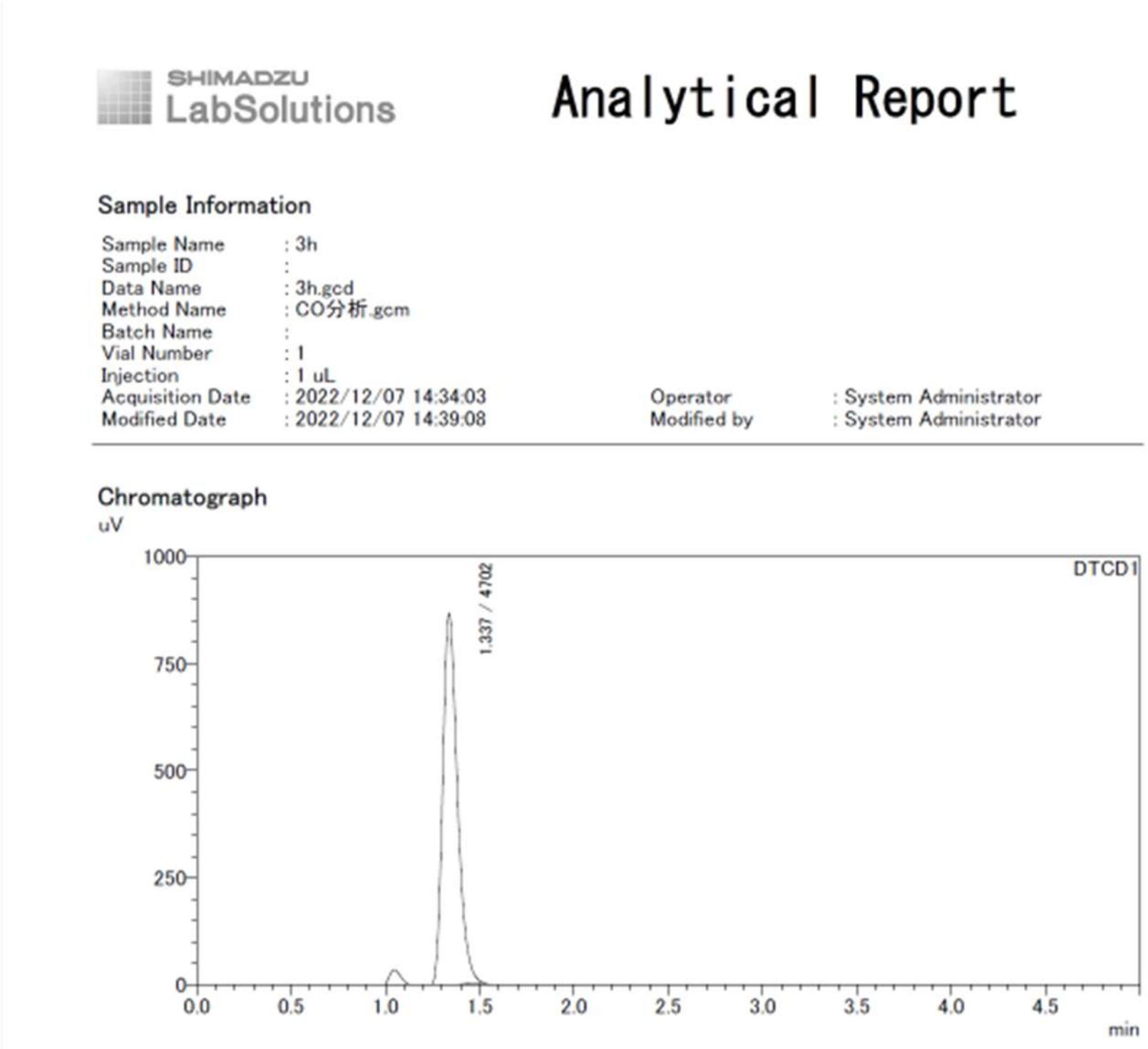

Chart S4. Chromatogram of CO in Table 1 (Entry 2).

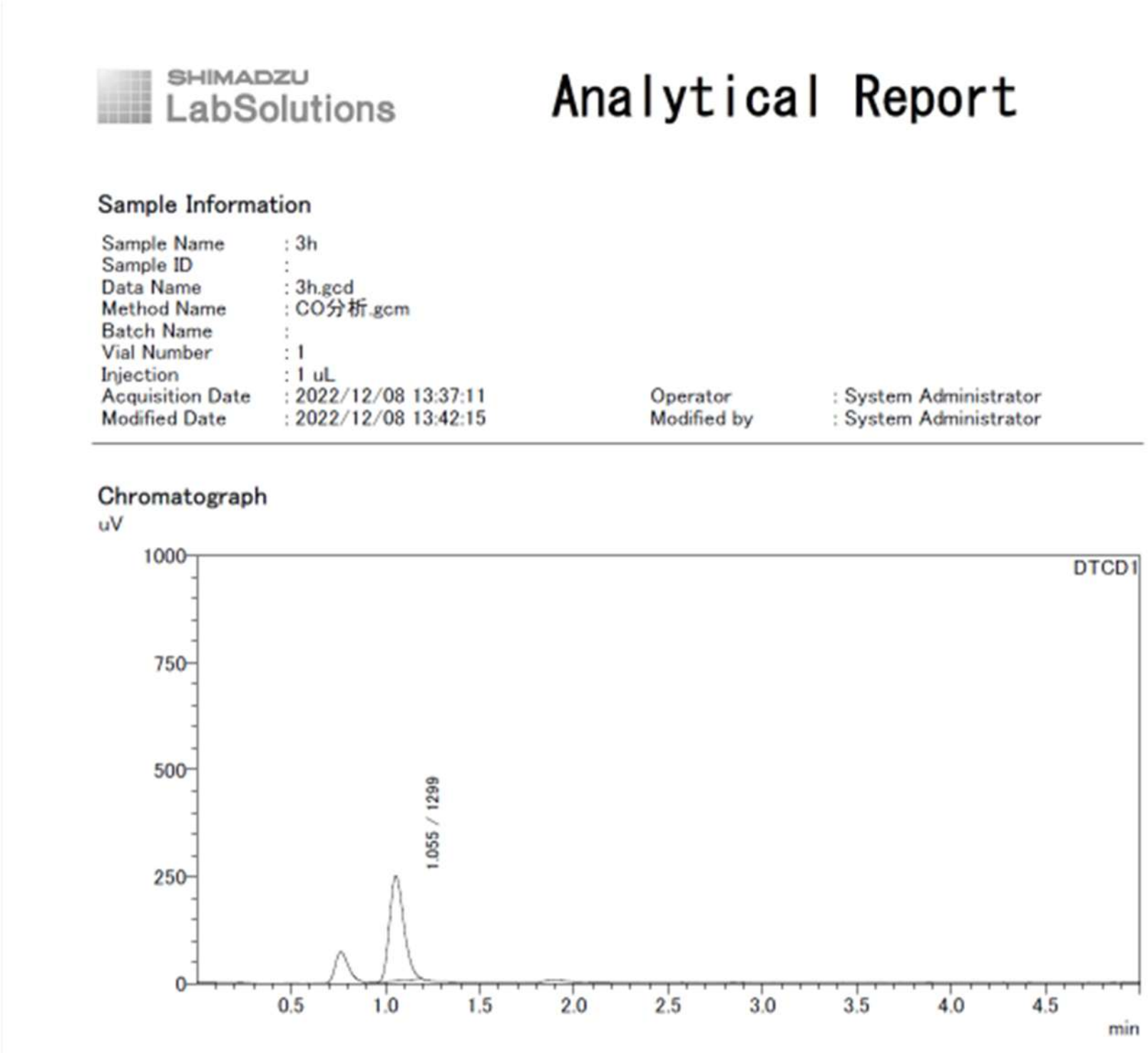

Chart S5. Chromatogram of CO in Table 1 (Entry 3).

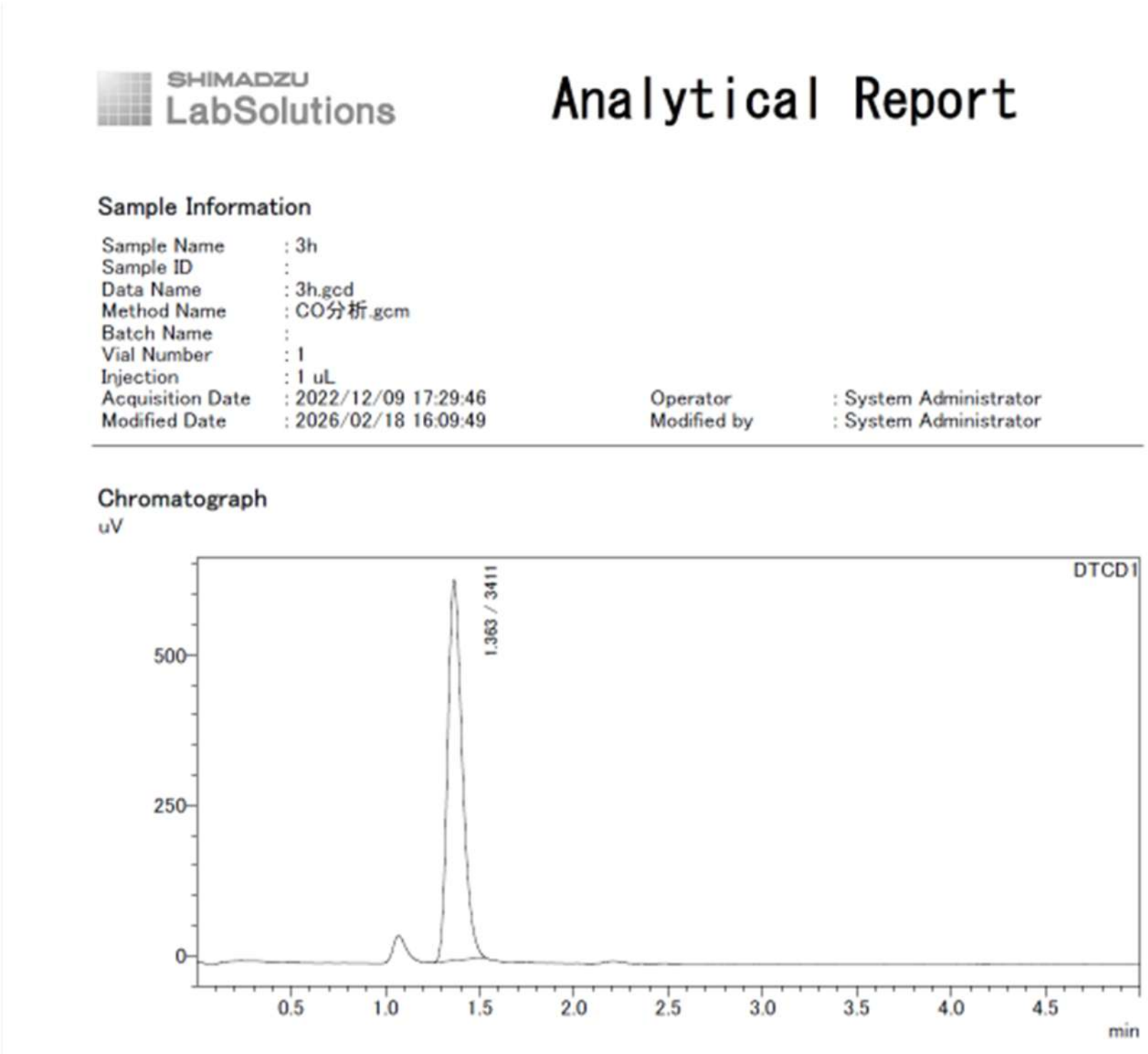

Chart S6. Chromatogram of CO in Table 1 (Entry 4).

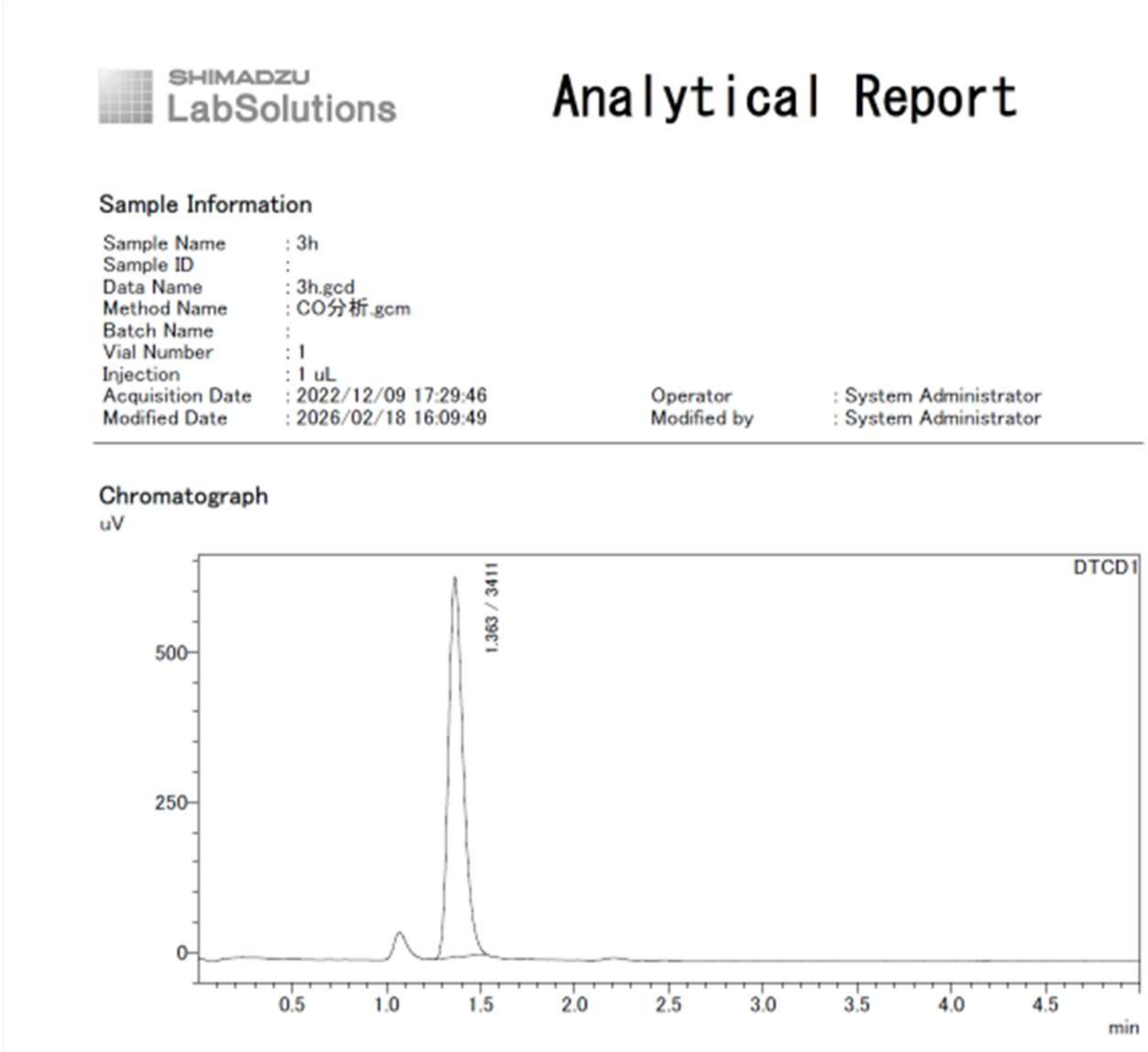

Chart S7. Chromatogram of CO in Table 1 (Entry 5).

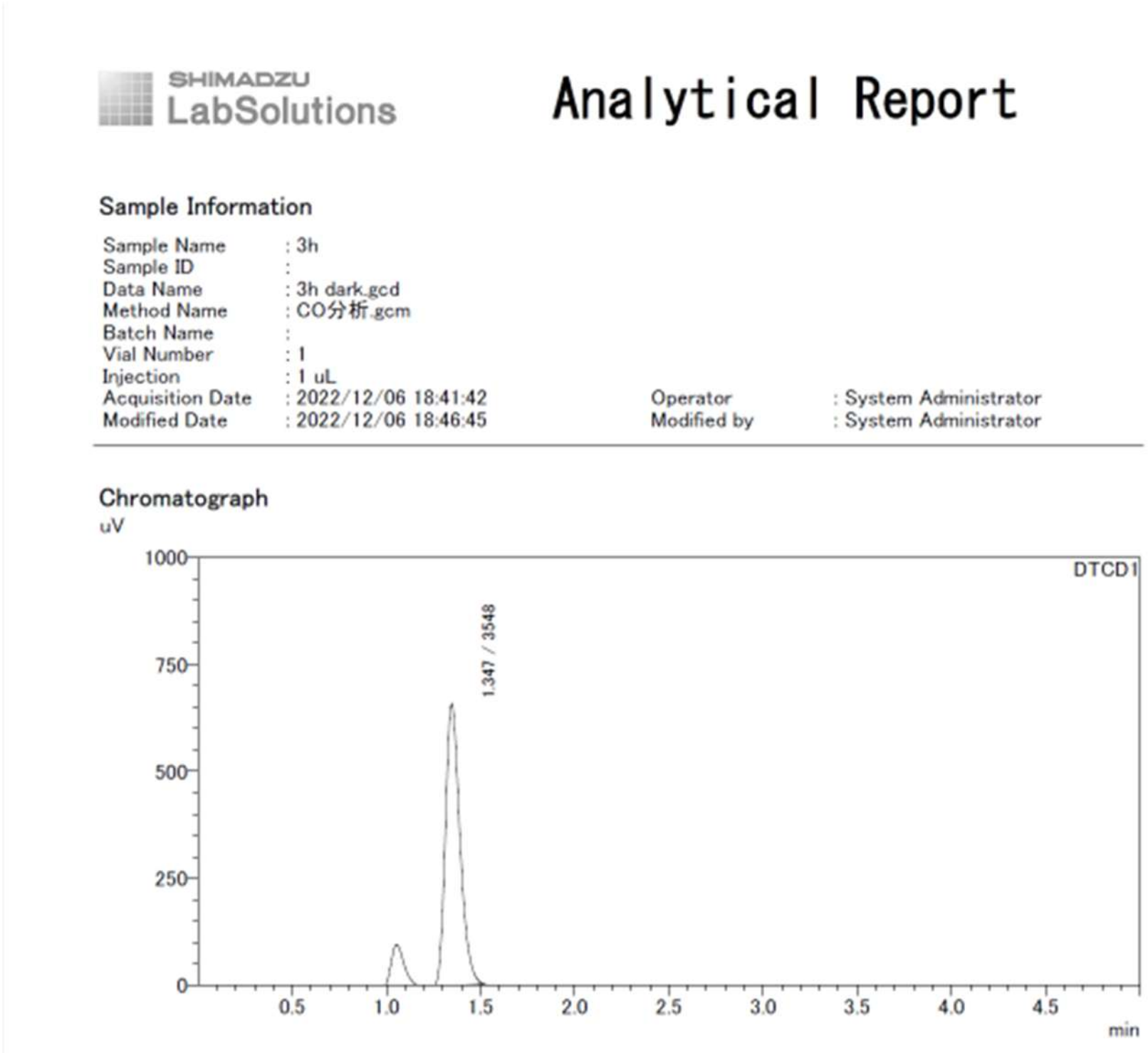

Chart S8. Chromatogram of CO in Table 1 (Entry 6).

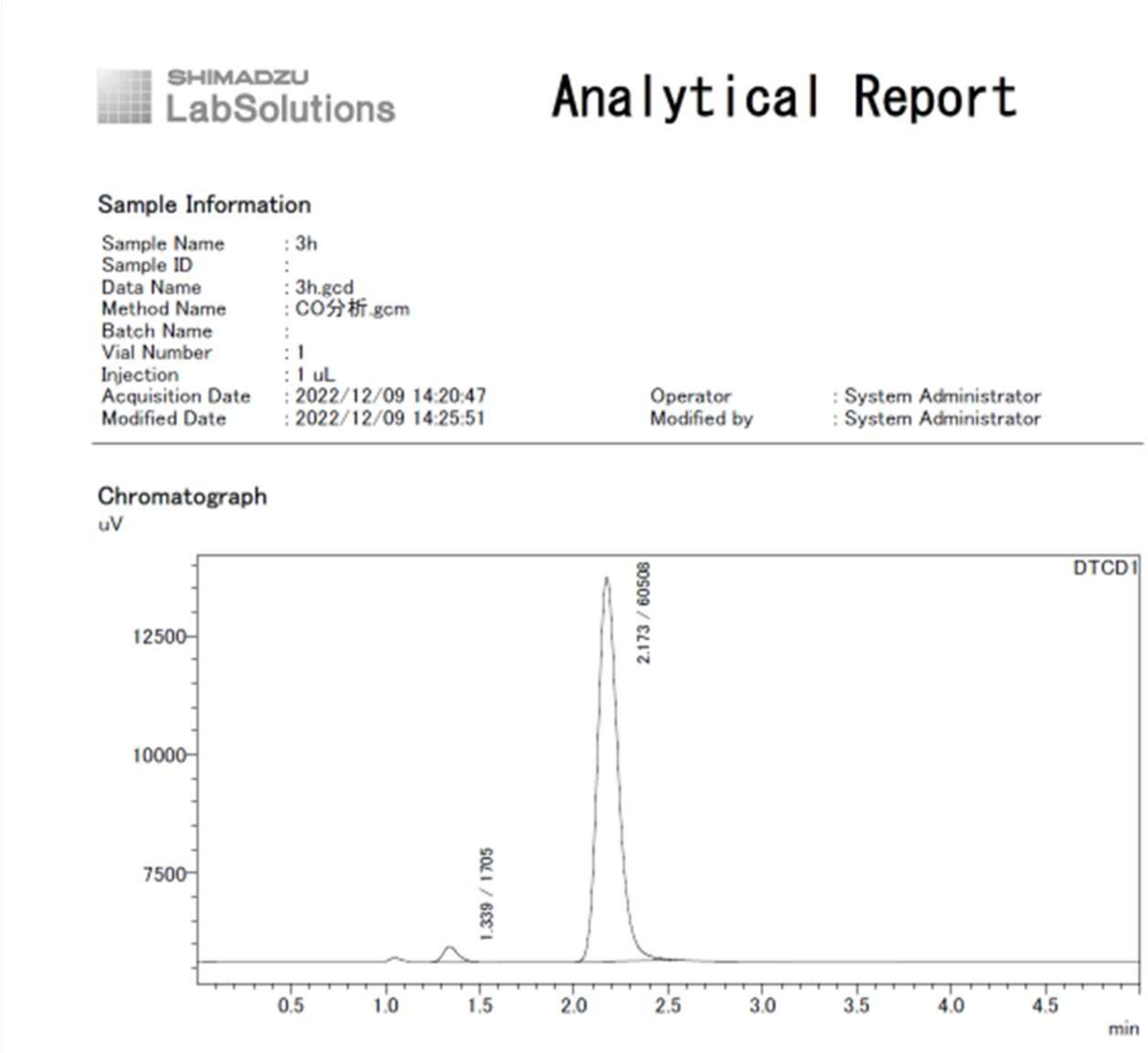

Chart S9. Chromatogram of CO in Table 1 (Entry 7).

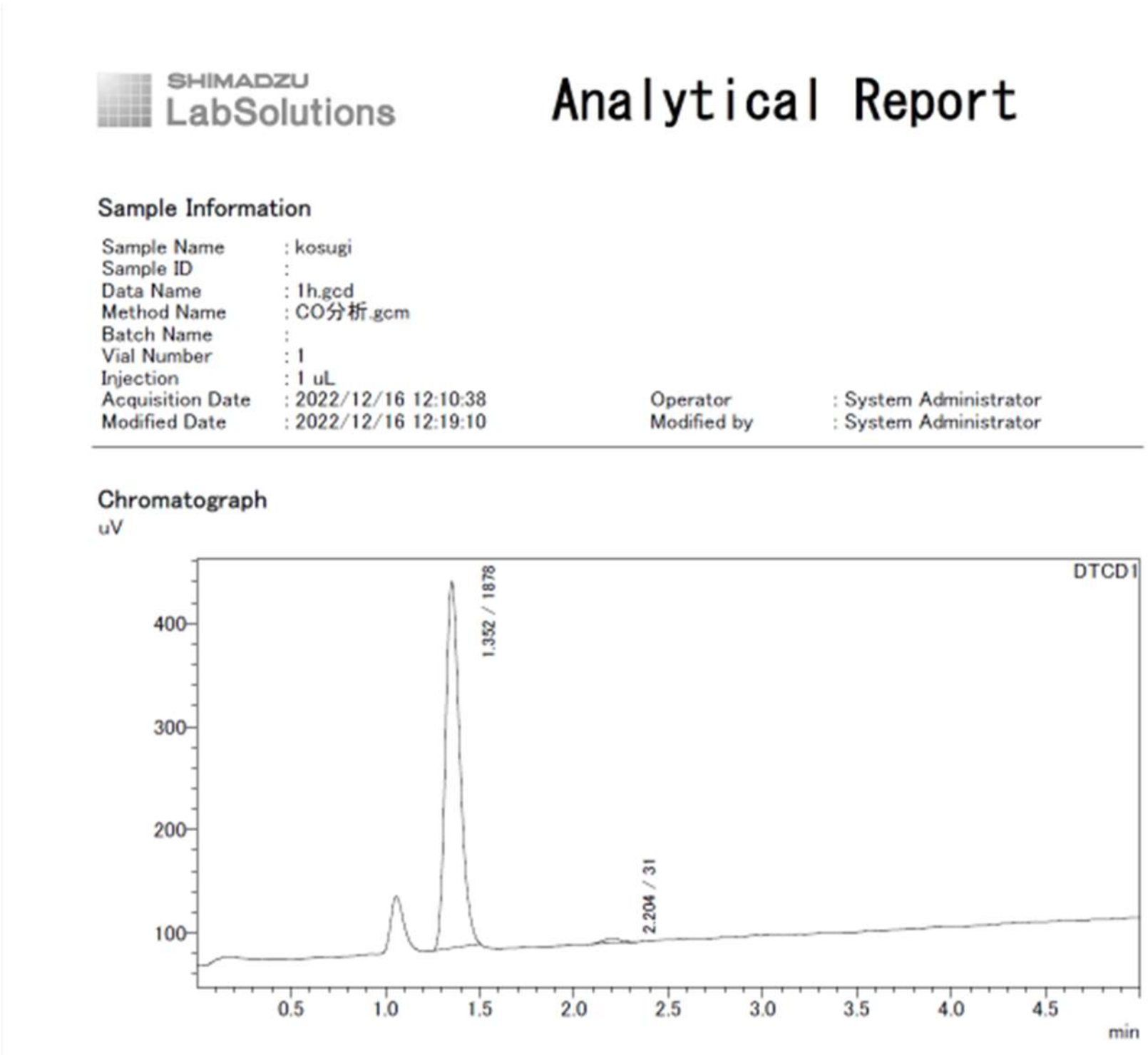

Chart S10. Chromatogram of CO shown in Fig. S7 (filtrate, 1 h).

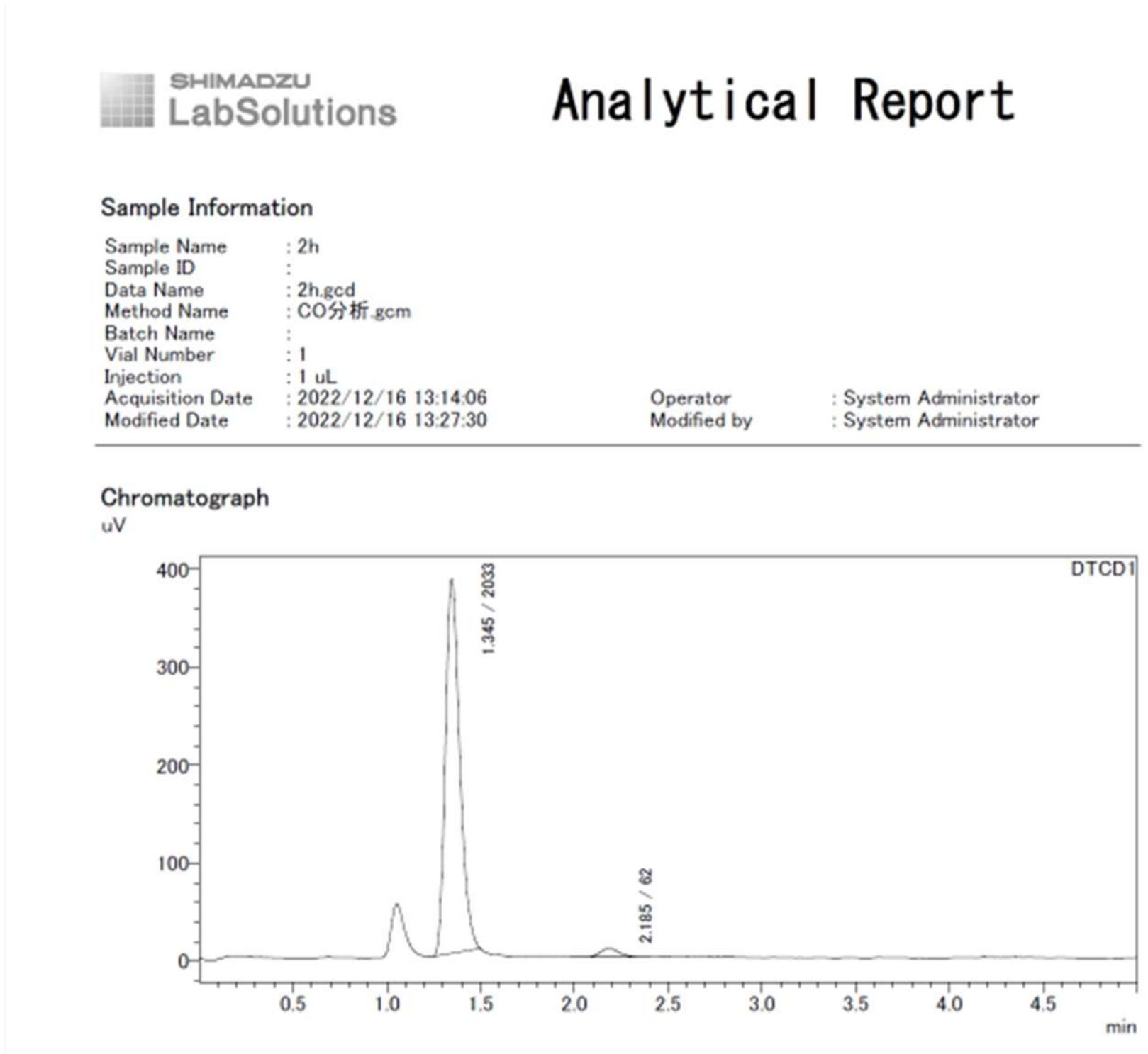

Chart S11. Chromatogram of CO shown in Fig. S7 (filtrate, 2 h).

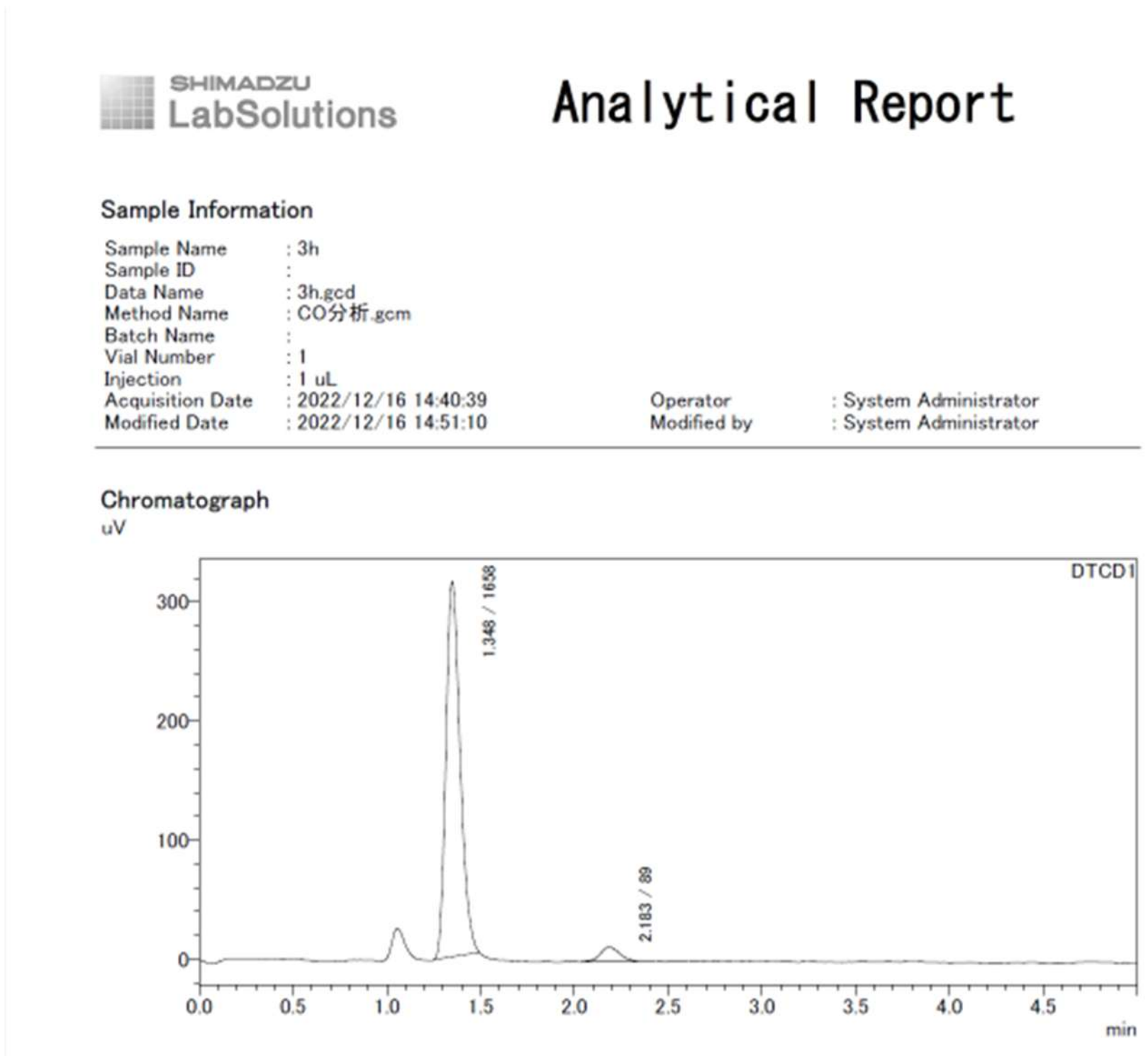

Chart S12. Chromatogram of CO shown in Fig. S7 (filtrate, 3 h).

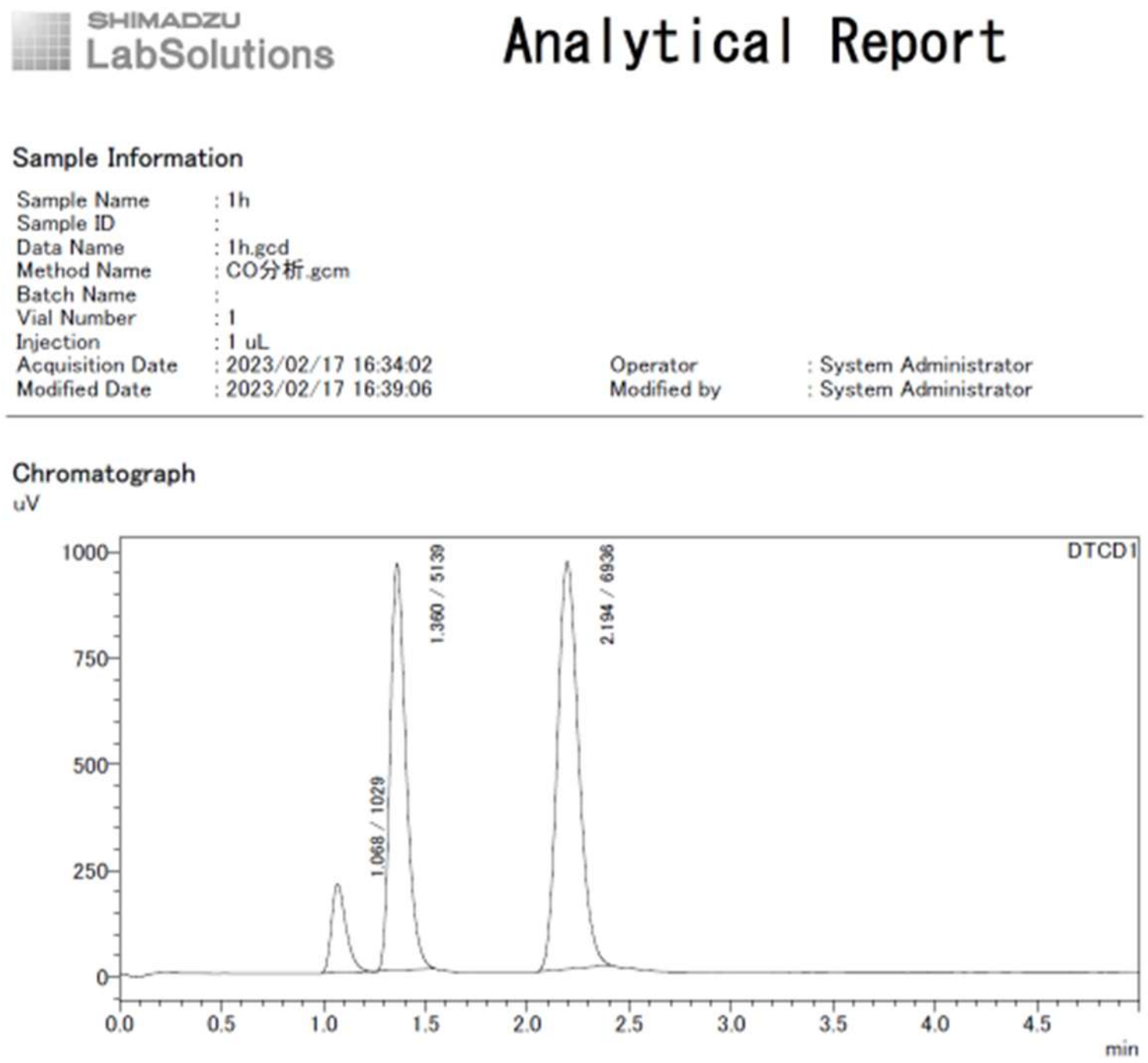

Chart S13. Chromatogram of CO in Fig. 2b when **[Fe(H4P)]cryst** was used as a catalyst (1 h).

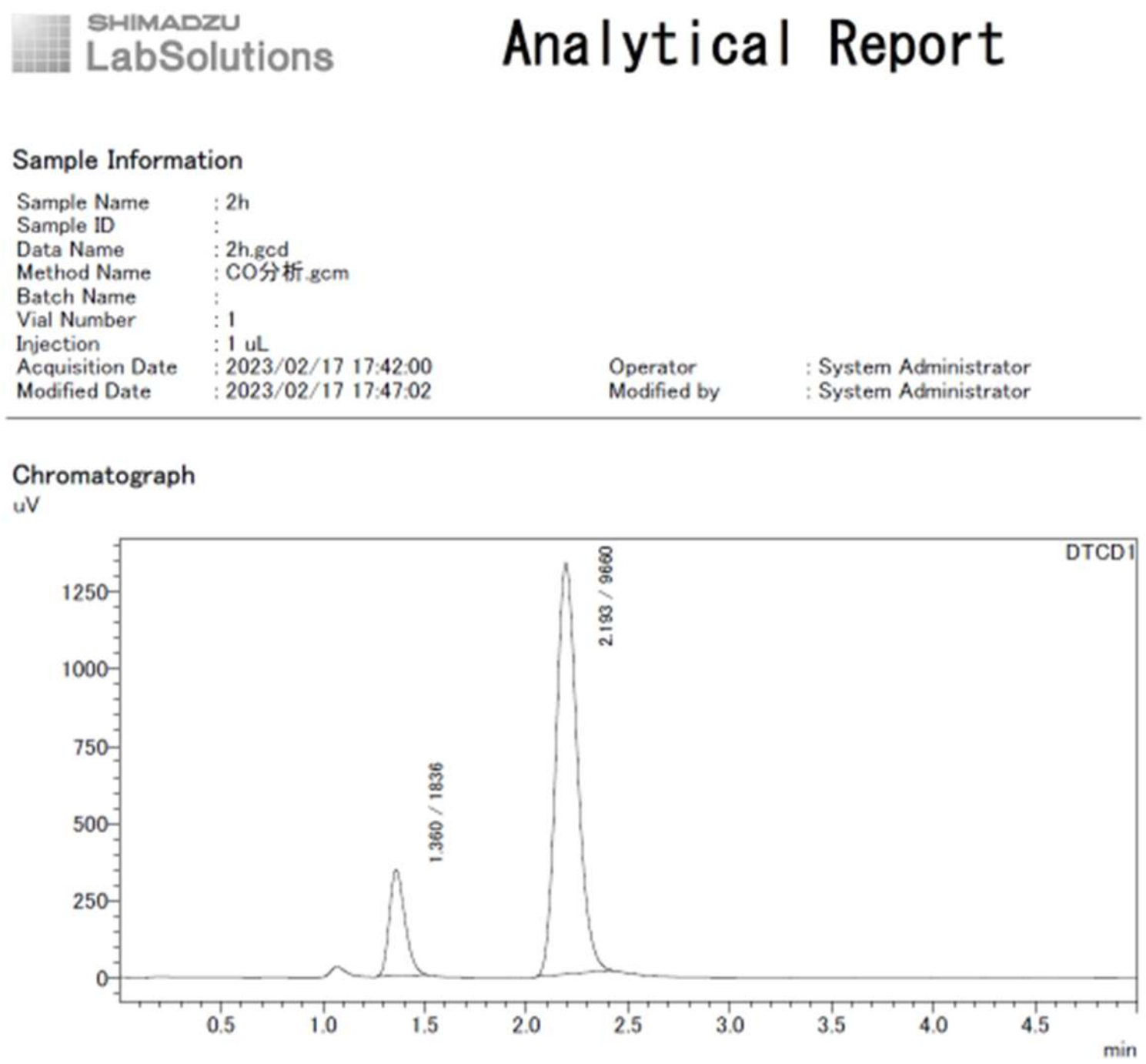

Chart S14. Chromatogram of CO in Fig. 2b when **[Fe(H4P)]cryst** was used as a catalyst (2 h).

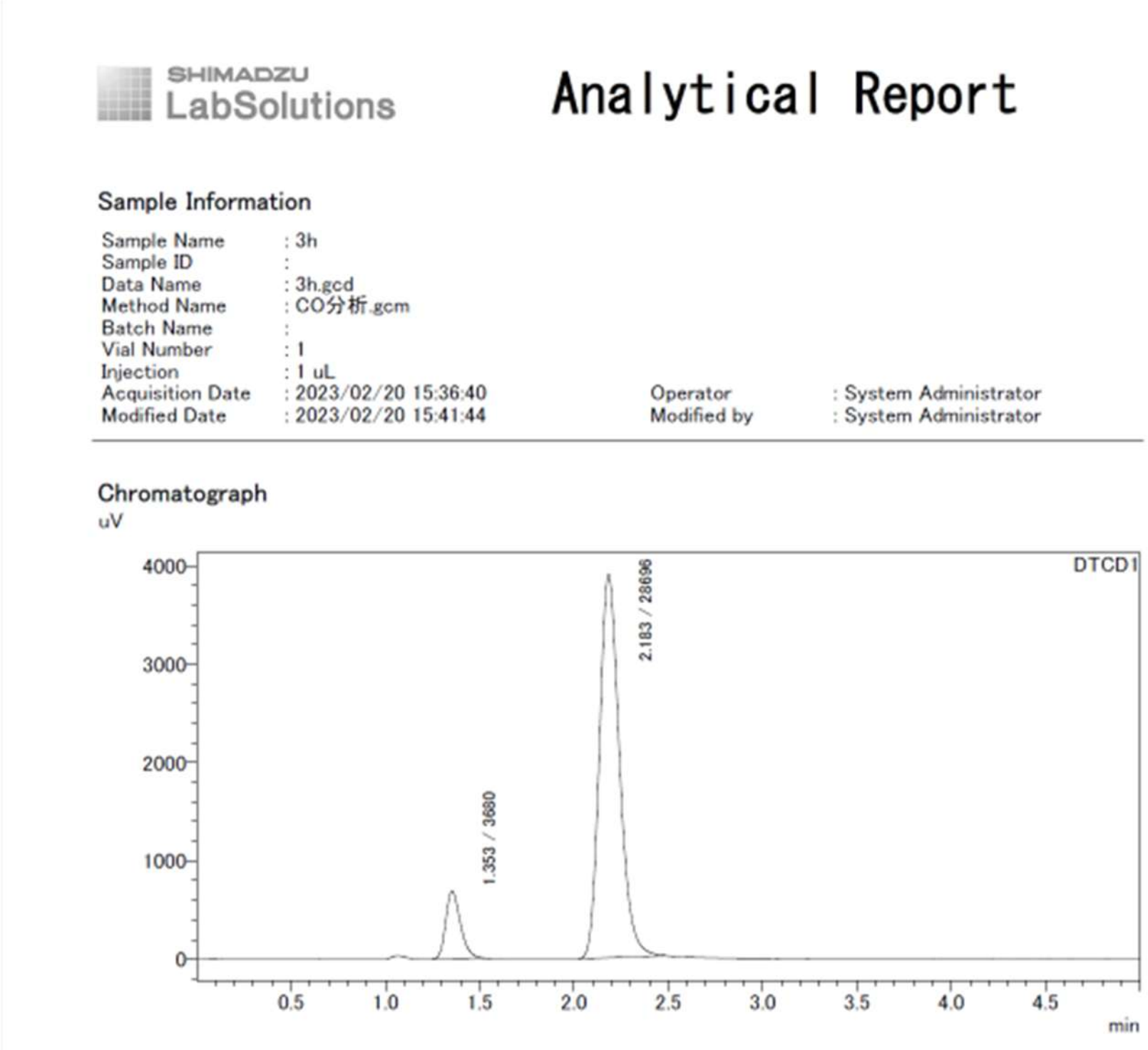

Chart S15. Chromatogram of CO in Fig. 2b when **[Fe(H4P)]cryst** was used as a catalyst (3 h).

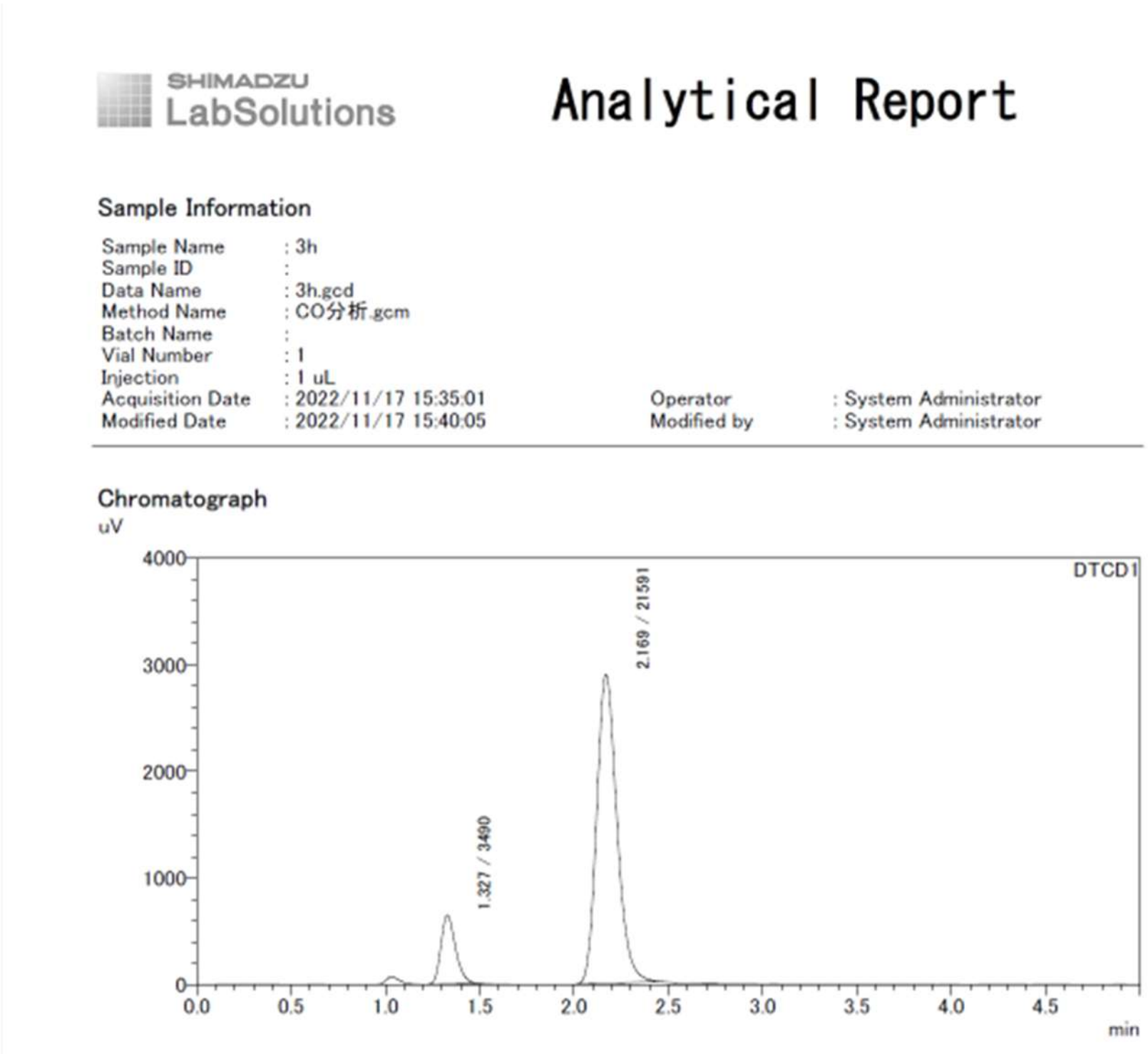

Chart S16. Chromatogram of CO when **[Fe(H3P)]cryst** and **Ru(bpy)3Cl2** were used as a catalyst and photosensitizer, respectively (3 h).

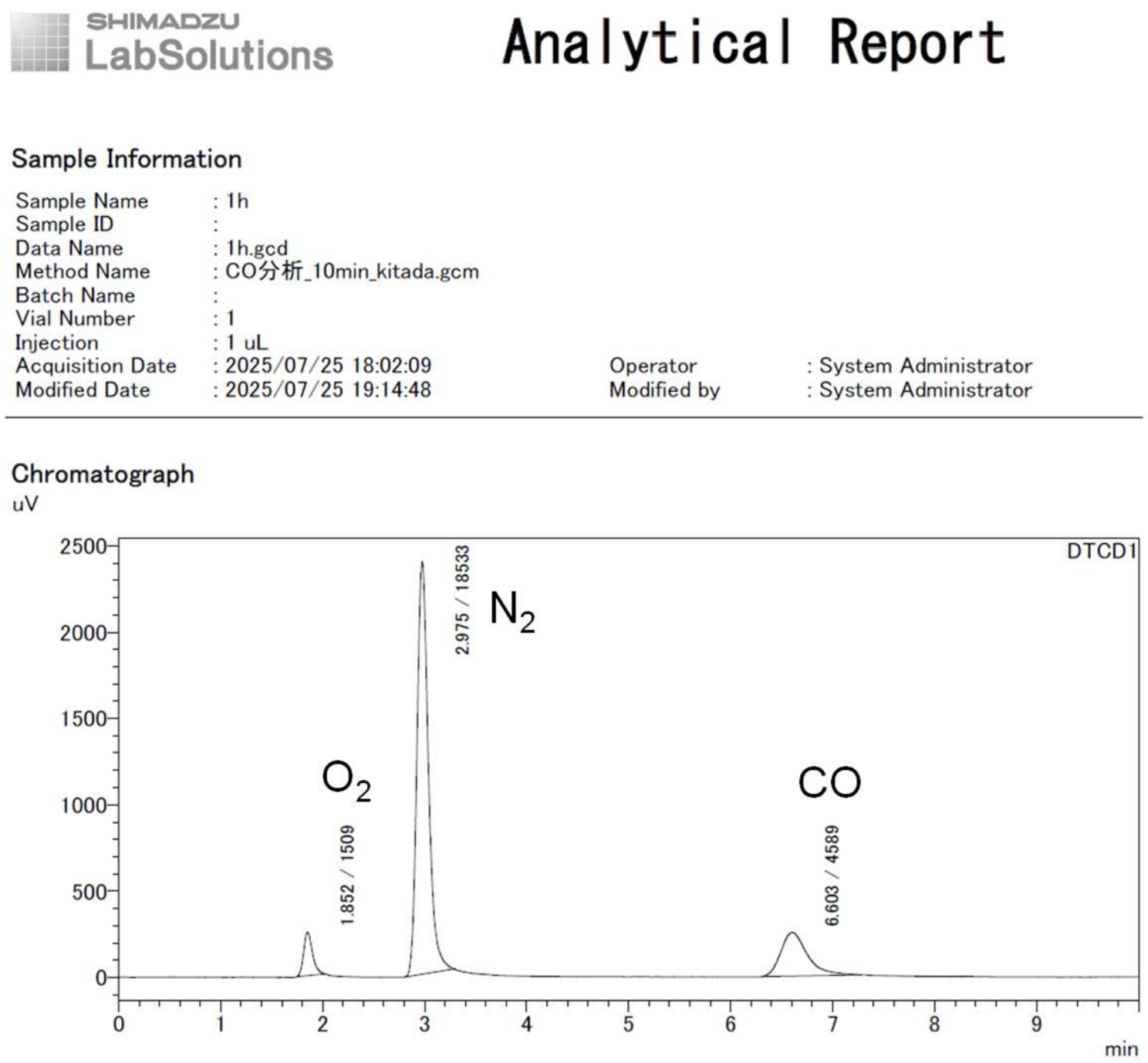

Chart S17. Chromatogram of CO in Fig. 2b when **[FeP]$_{\mathbf{cryst}}$** was used as a catalyst (1 h).

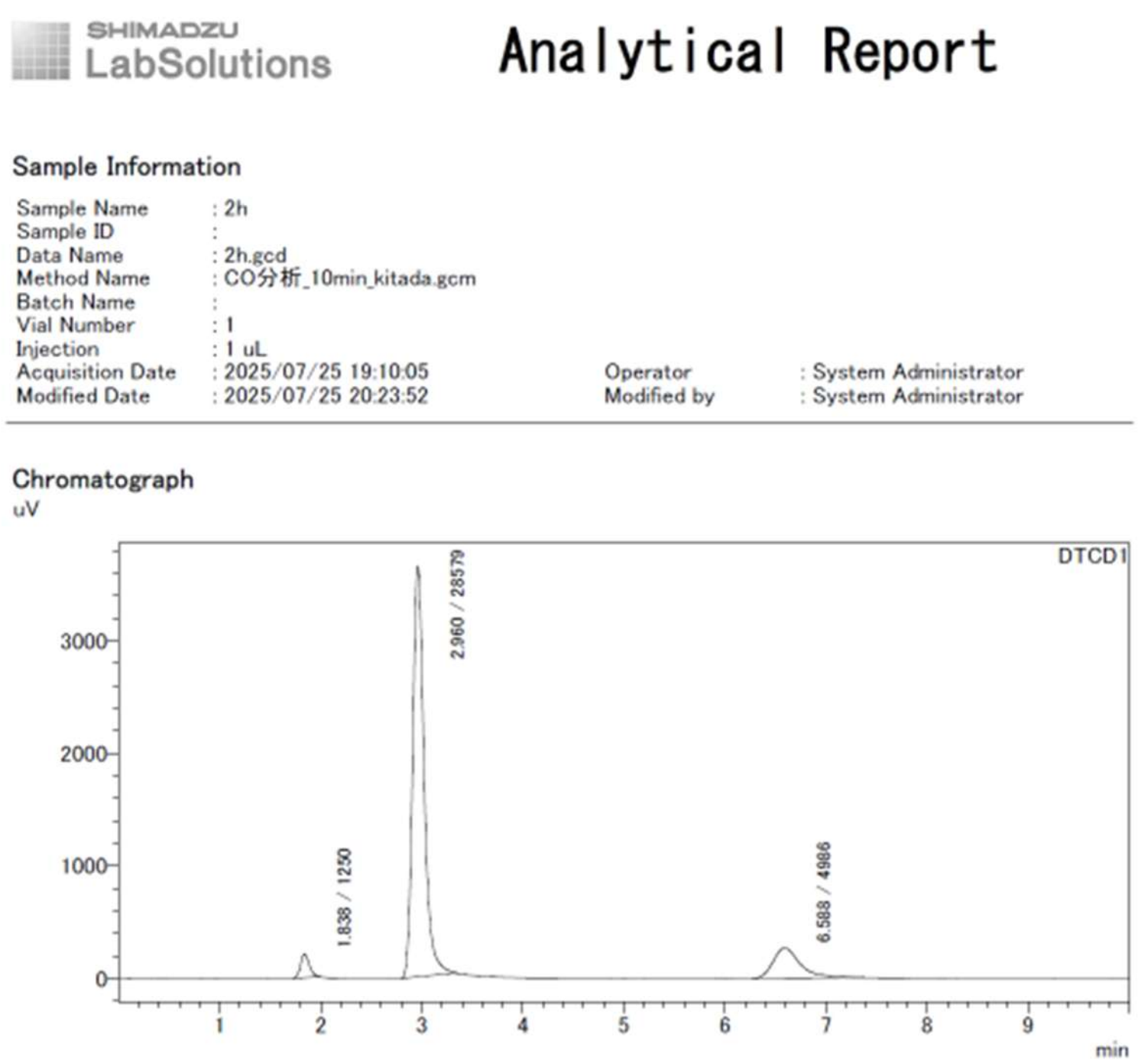

Chart S18. Chromatogram of CO in Fig. 2b when **[FeP]$_{\mathbf{cryst}}$** was used as a catalyst (2 h).

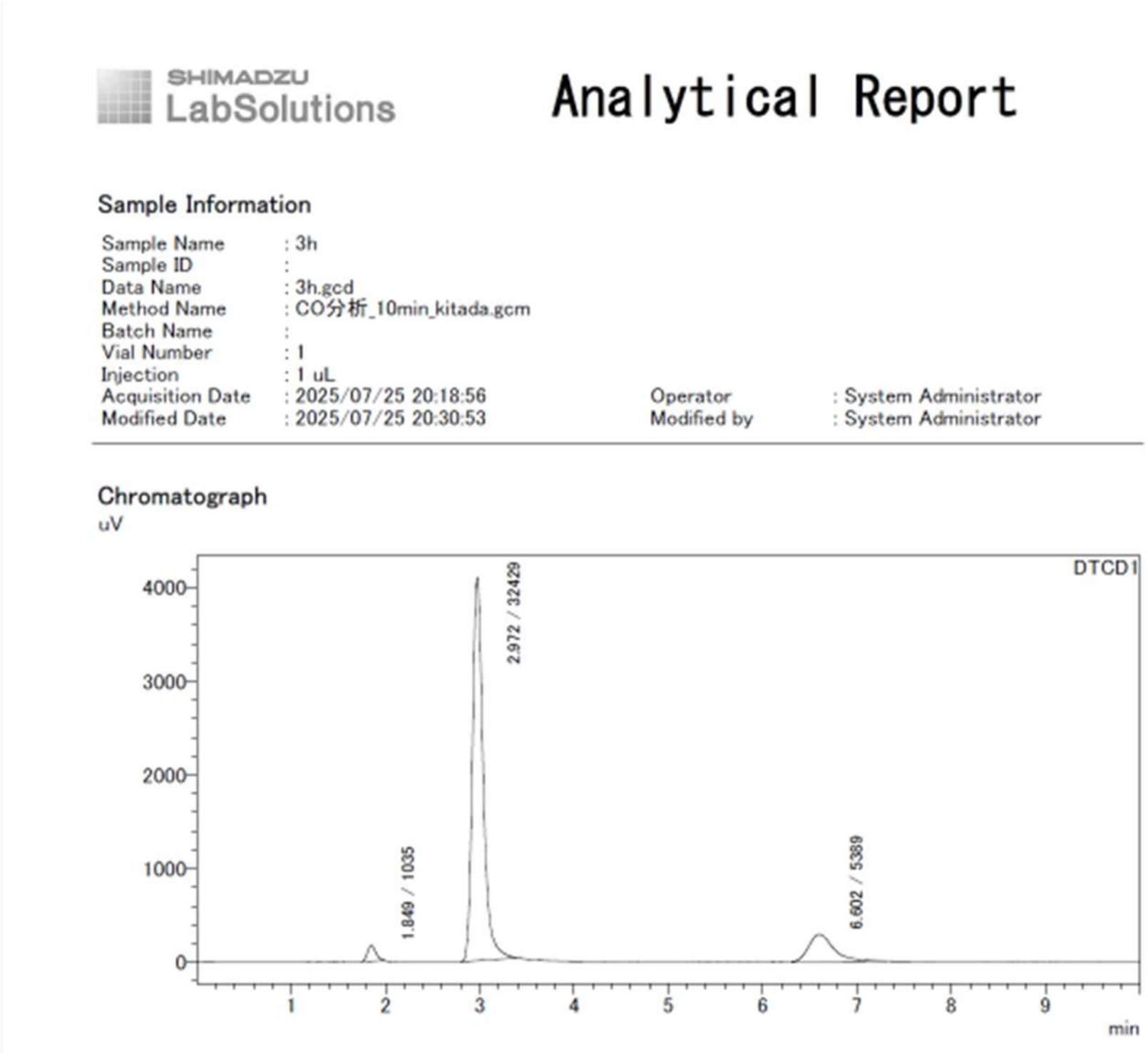

Chart S19. Chromatogram of CO in Fig. 2b when **[FeP]$_{\mathbf{cryst}}$** was used as a catalyst (3 h).

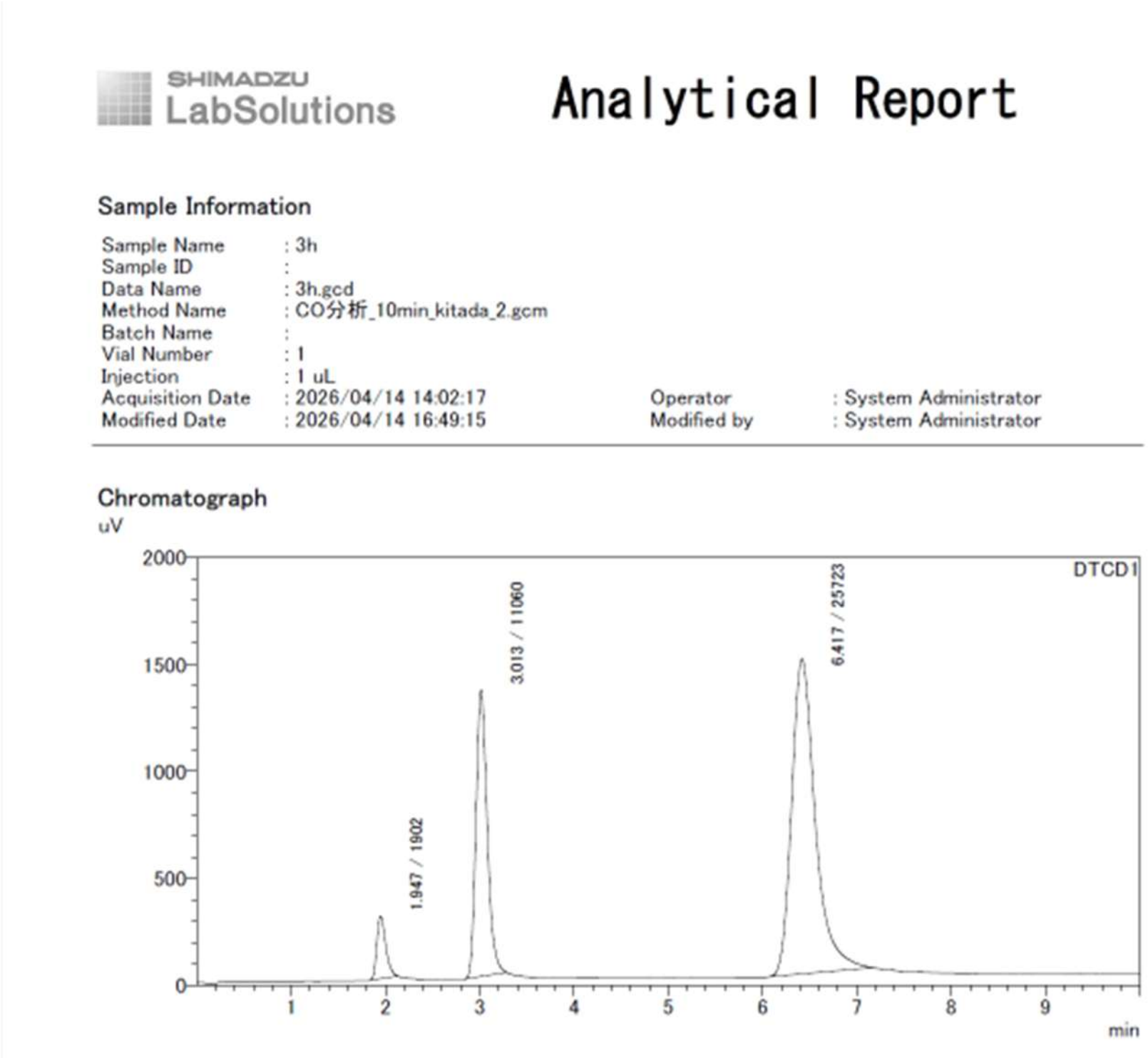

Chart S20. Chromatogram of CO for quantum yield determination (3 h).

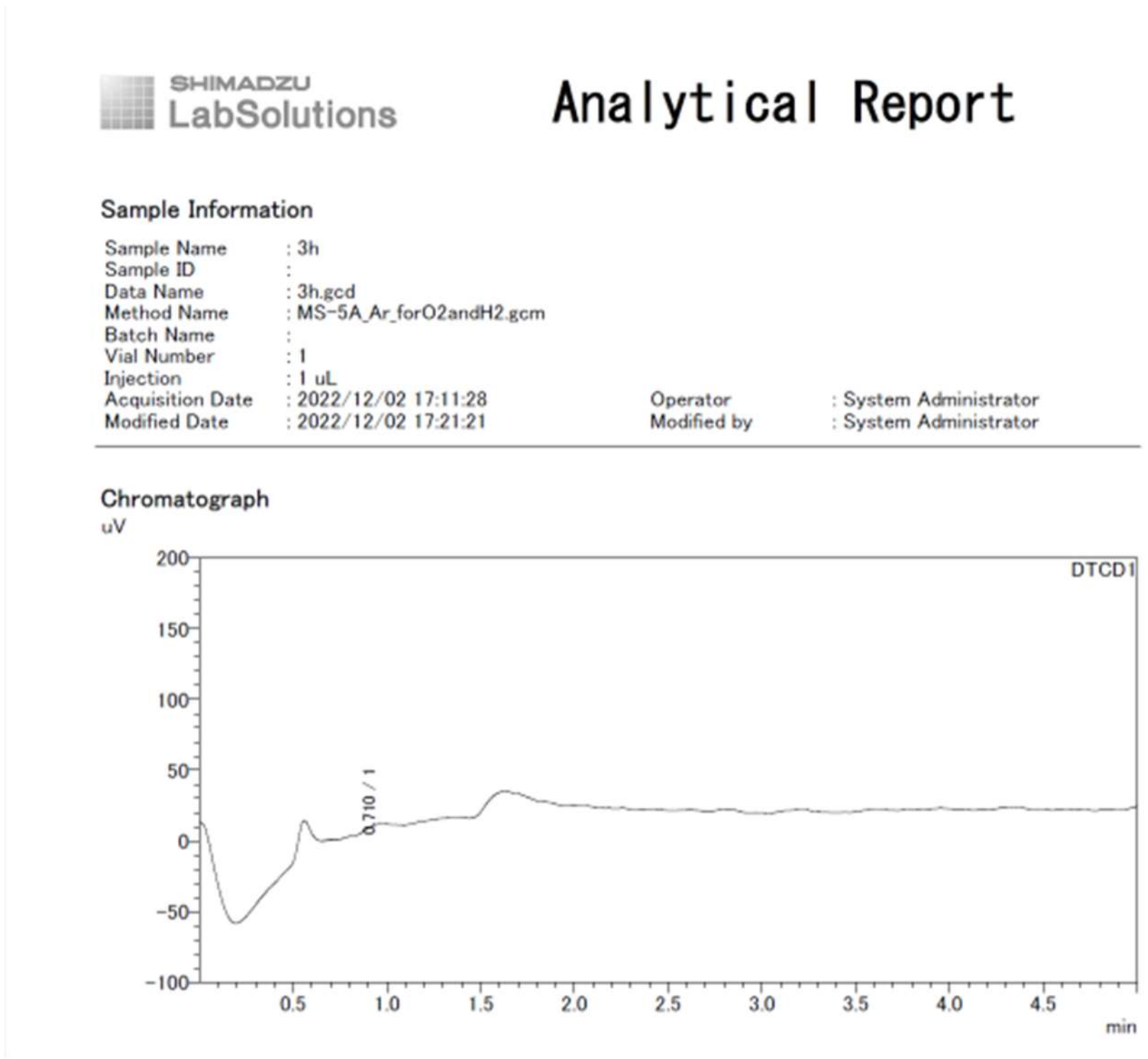

Chart S21. Chromatogram of H_2 in Table 1 (Entry 1).

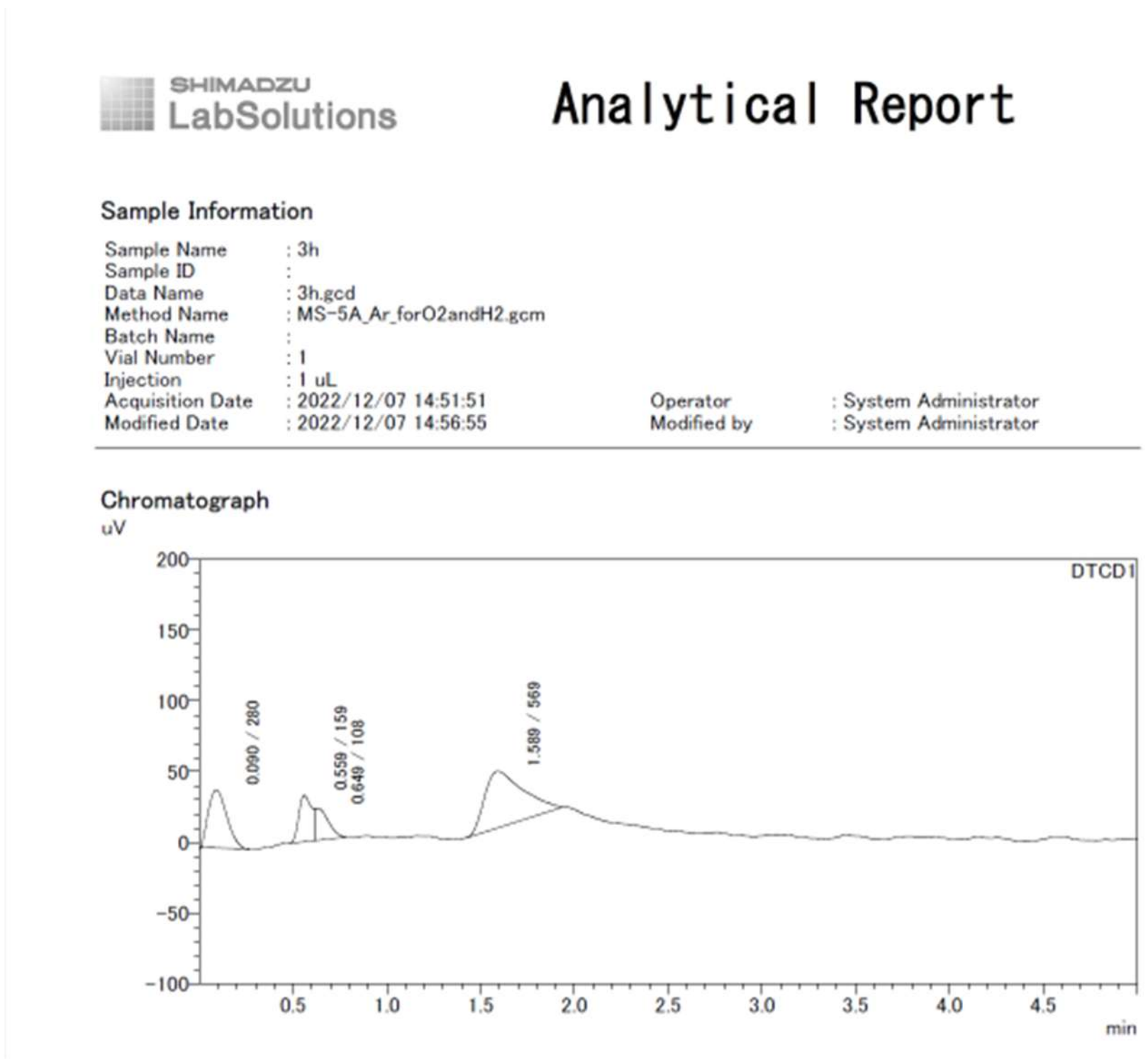

Chart S22. Chromatogram of H_2 in Table 1 (Entry 2).

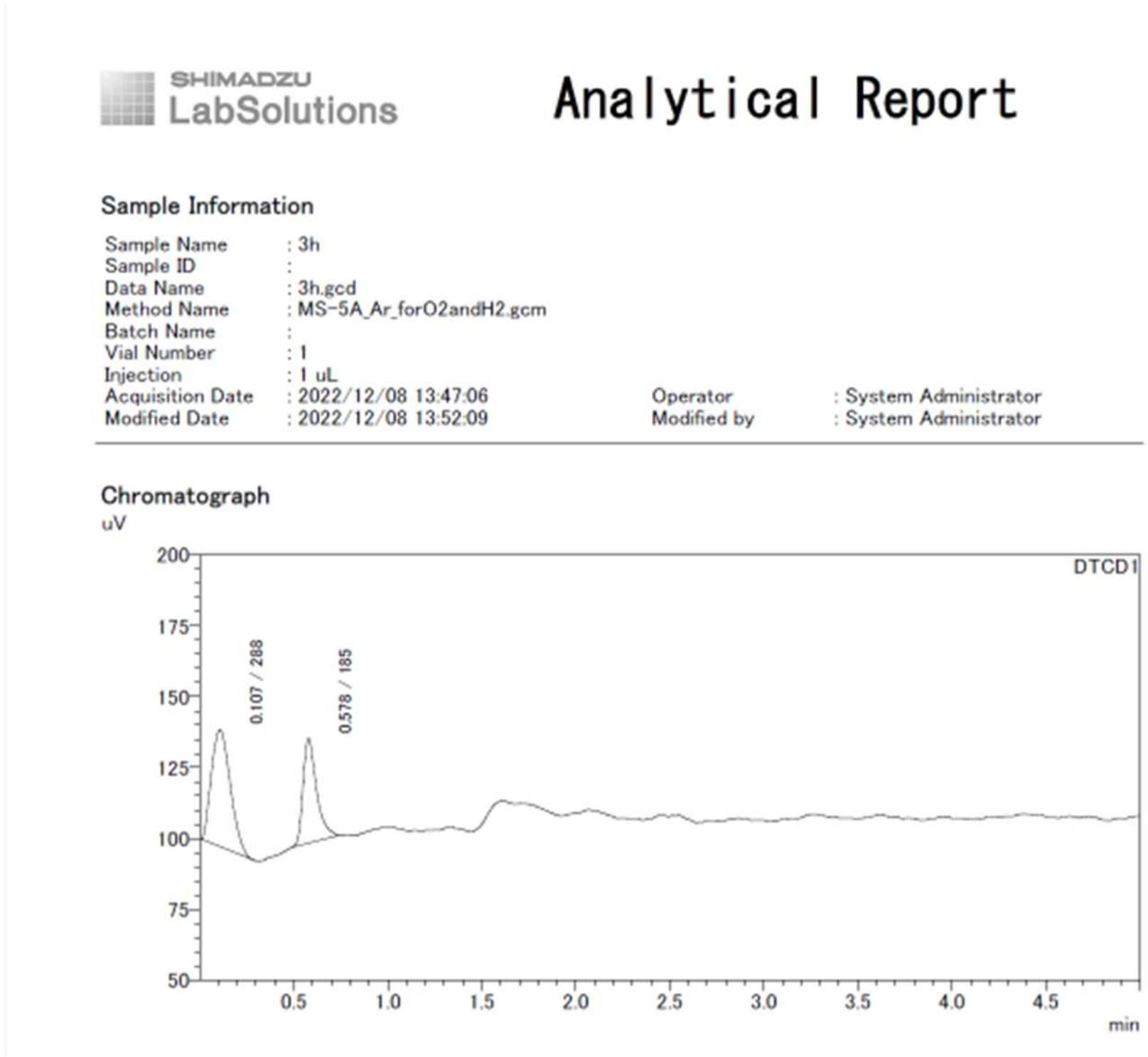

Chart S23. Chromatogram of H_2 in Table 1 (Entry 3).

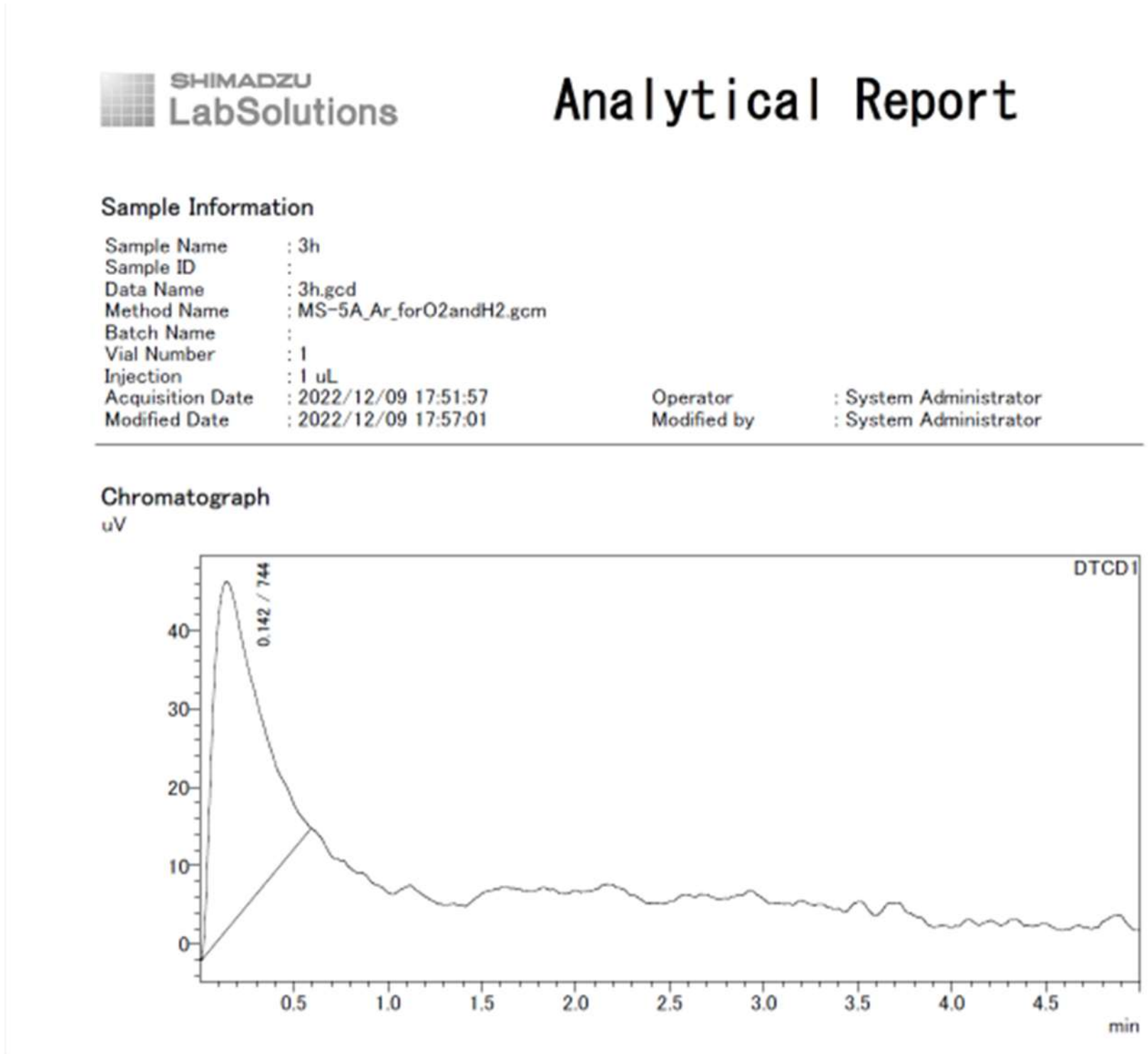

Chart S24. Chromatogram of H_2 in Table 1 (Entry 4).

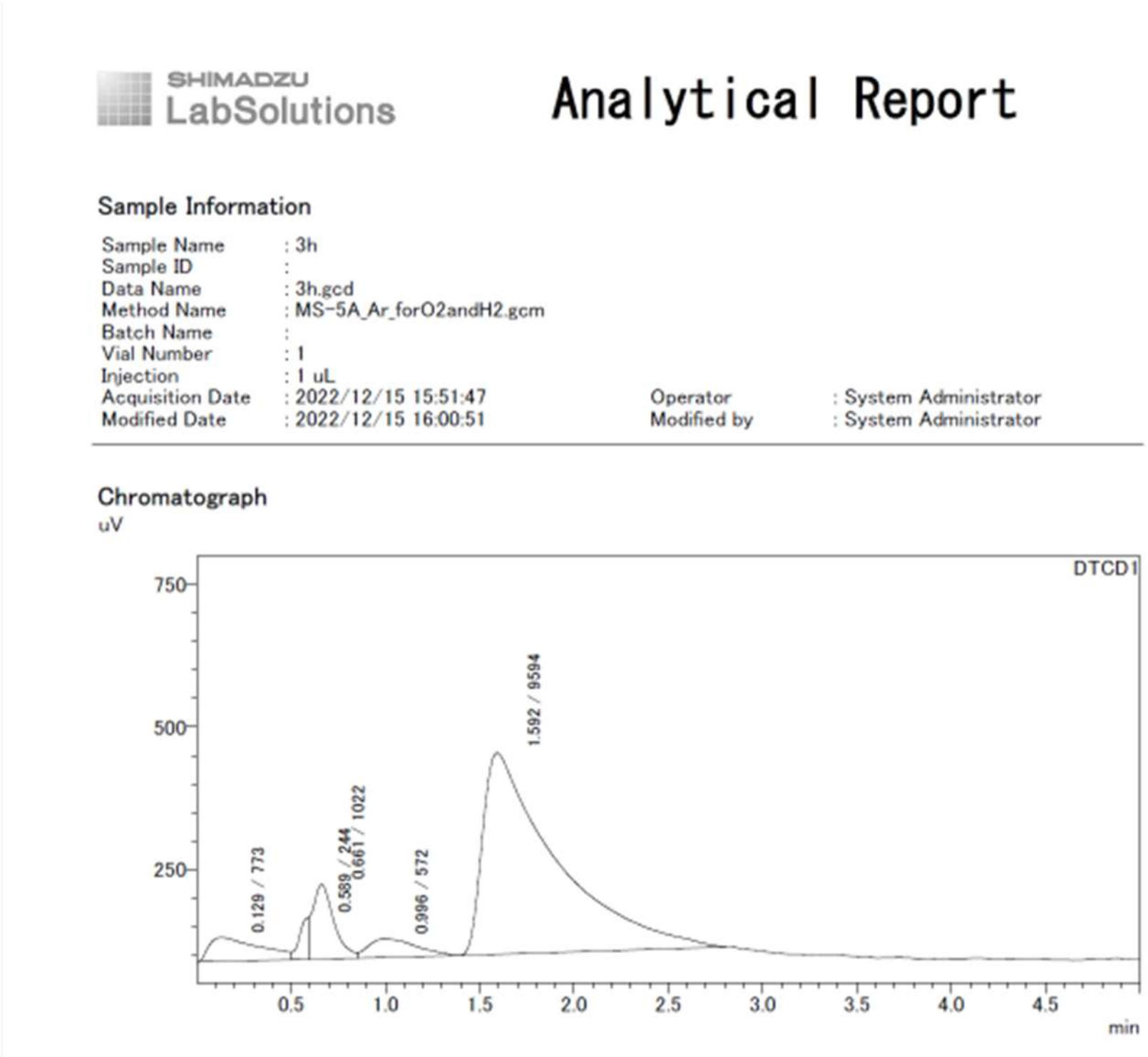

Chart S25. Chromatogram of H_2 in Table 1 (Entry 5).

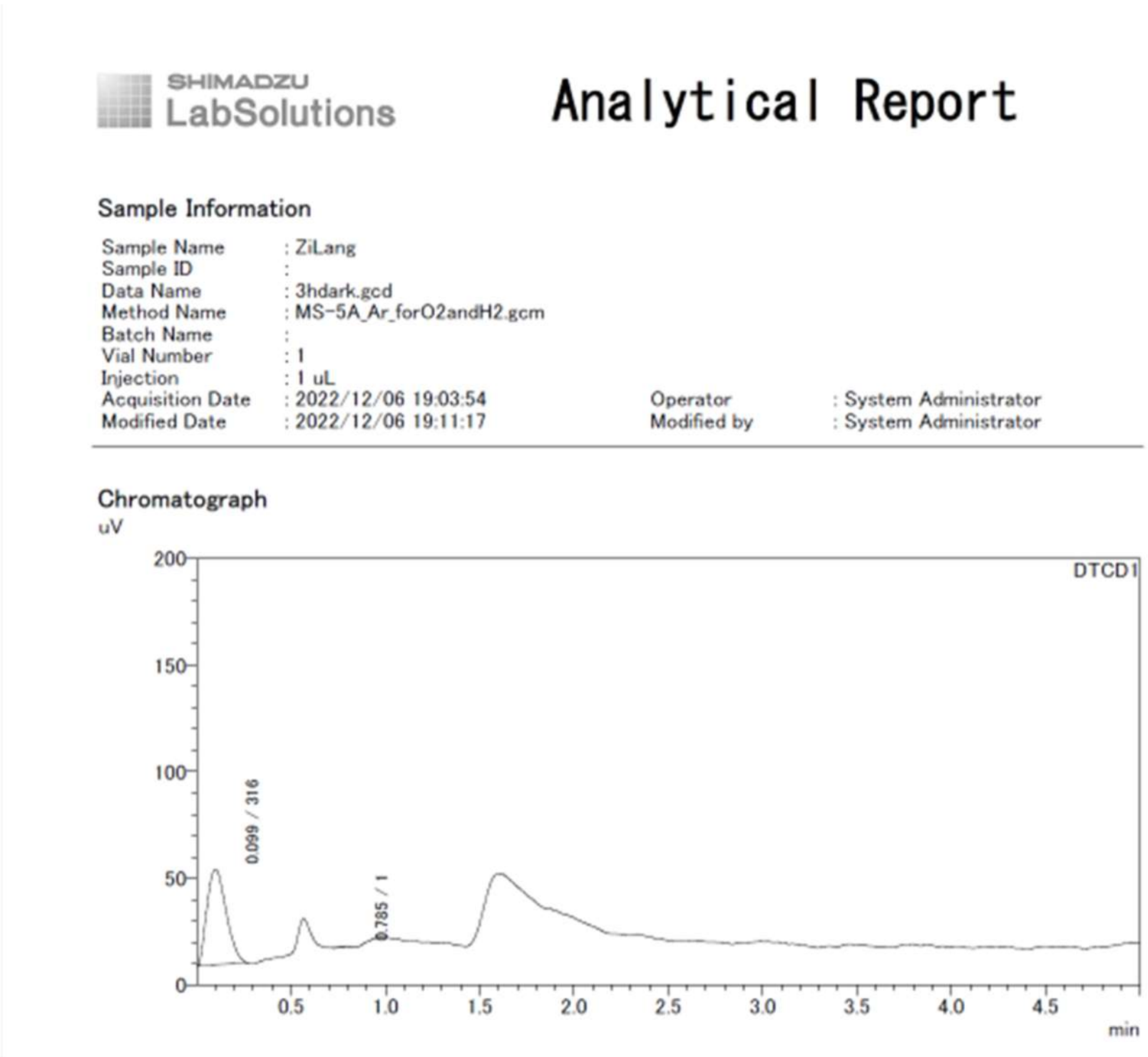

Chart S26. Chromatogram of H_2 in Table 1 (Entry 6).

SHIMADZU LabSolutions

Analytical Report

Sample Information

Sample Name	: 3h
Sample ID	:
Data Name	: 3h.gcd
Method Name	: MS-5A_Ar_forO2andH2.gcm
Batch Name	:
Vial Number	: 1
Injection	: 1 uL
Acquisition Date	: 2022/12/09 14:43:16
Modified Date	: 2023/02/28 12:17:19

Operator	: System Administrator
Modified by	: System Administrator

Chromatograph

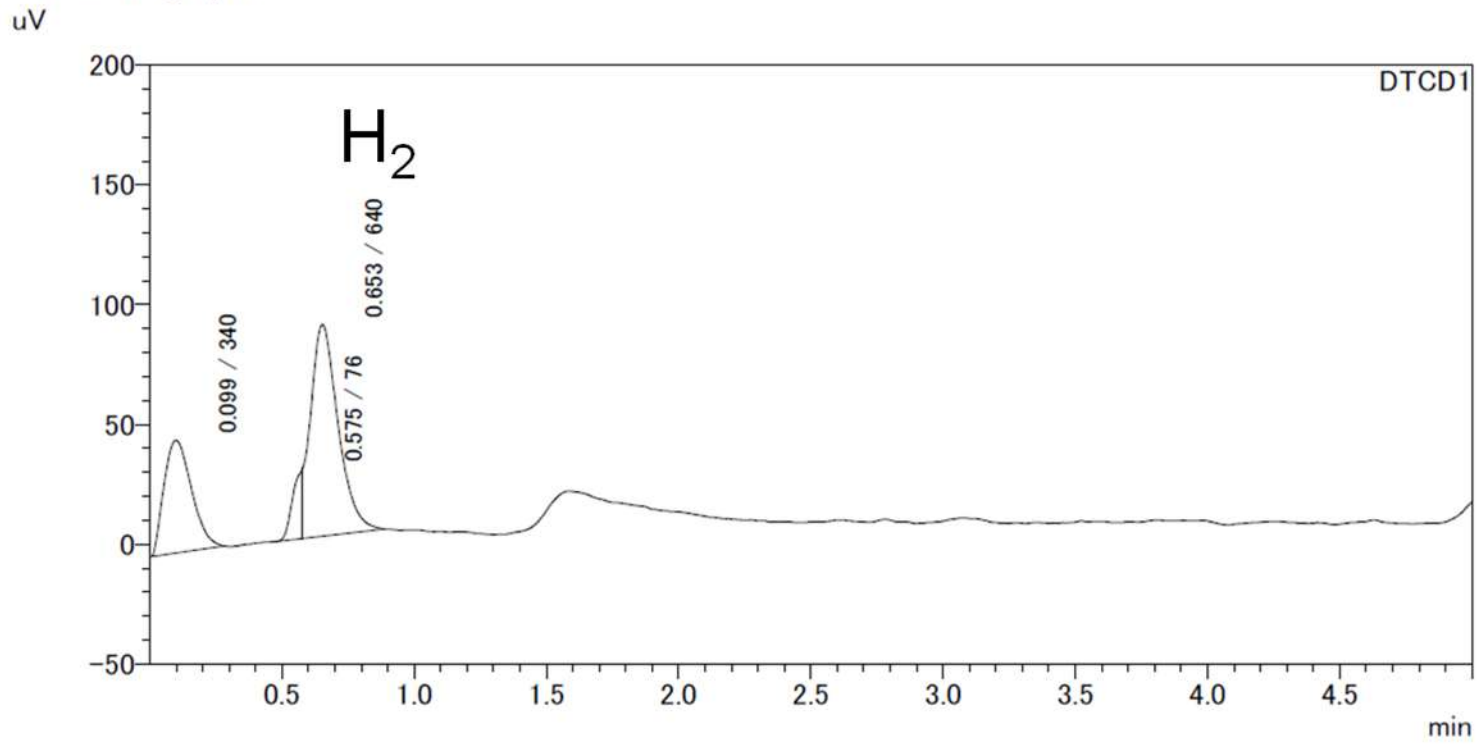

Chart S27. Chromatogram of H_2 in Table 1 (Entry 7).

15. MALDI-TOF-MS

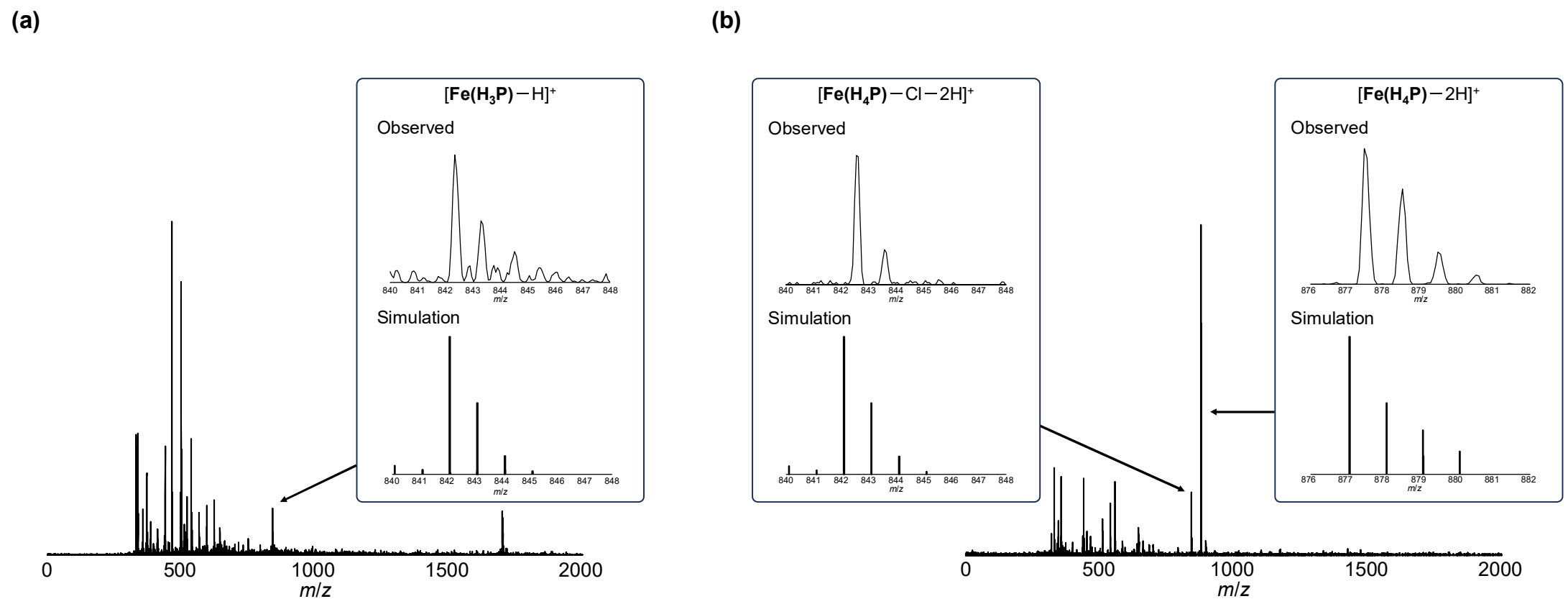

Chart S28. MALDI-TOF-MS spectra of (a) **Fe(H$_3$P)** and (b) **Fe(H$_4$P)**.

16. NMR chart

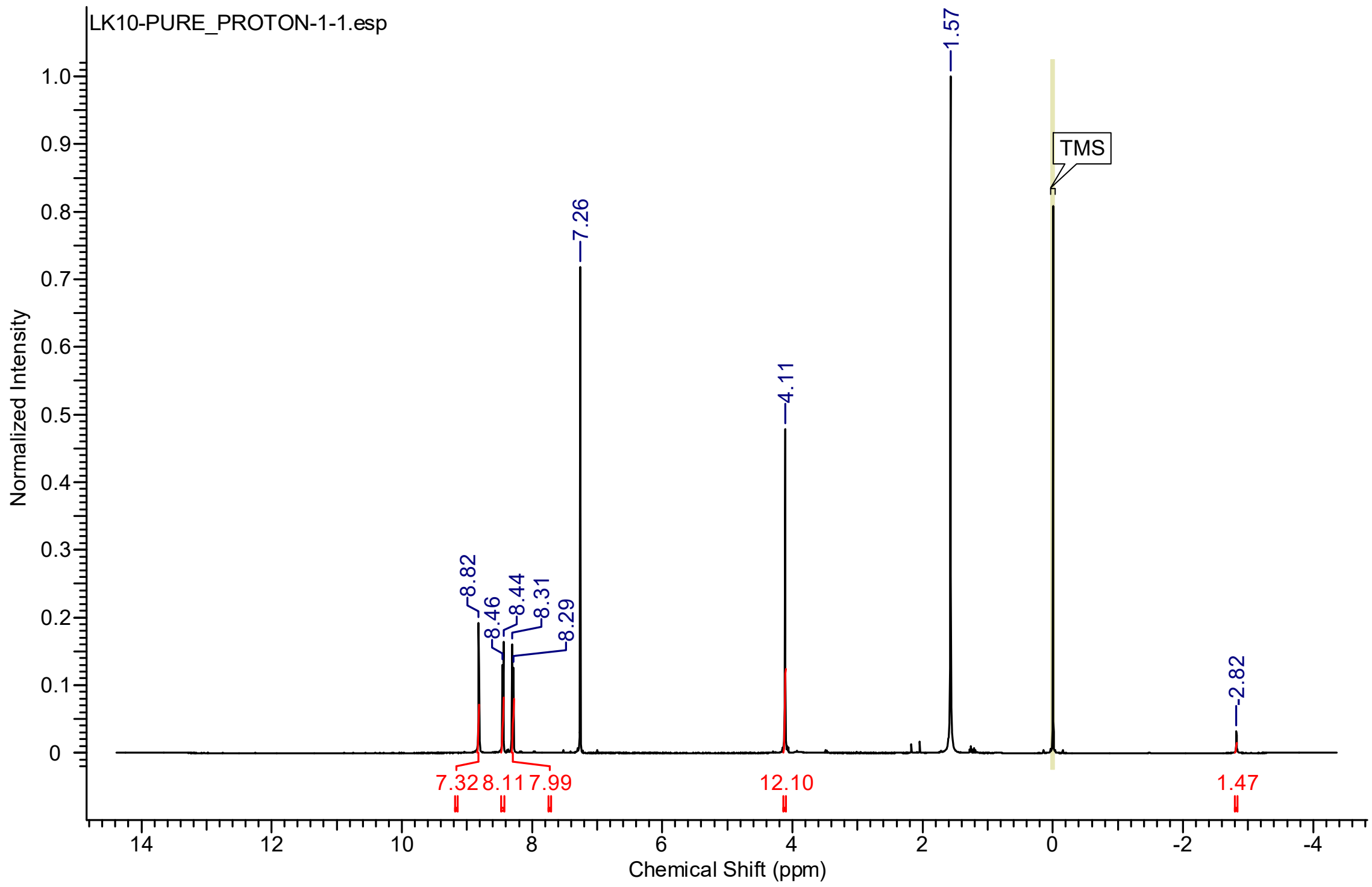

Chart S29. ^{1}H-NMR spectrum of **Me₄P** in chroloform-d_1.
